# Resolving the conflict between antibiotic production and rapid growth by recognition of peptidoglycan of susceptible competitors

**DOI:** 10.1038/s41467-021-27904-2

**Published:** 2022-01-20

**Authors:** Harsh Maan, Maxim Itkin, Sergey Malitsky, Jonathan Friedman, Ilana Kolodkin-Gal

**Affiliations:** 1 Department of Molecular Genetics, Weizmann Institute of Science, 234 Herzl Street, Rehovot, Israel; 2 Life Science Core Facilities Weizmann Institute of Science, 234 Herzl Street, Rehovot, Israel; 3 Department of Plant Pathology and Microbiology, The Robert H. Smith Faculty of Agriculture, Food & Environment, The Hebrew University of Jerusalem, Rehovot, Israel

**Keywords:** Bacteriology, Microbial ecology, Bacterial genetics

## Abstract

Microbial communities employ a variety of complex strategies to compete successfully against competitors sharing their niche, with antibiotic production being a common strategy of aggression. Here, by systematic evaluation of four non-ribosomal peptides/polyketide (NRPs/PKS) antibiotics produced by *Bacillus subtilis* clade, we revealed that they acted synergistically to effectively eliminate phylogenetically distinct competitors. The production of these antibiotics came with a fitness cost manifested in growth inhibition, rendering their synthesis uneconomical when growing in proximity to a phylogenetically close species, carrying resistance against the same antibiotics. To resolve this conflict and ease the fitness cost, antibiotic production was only induced by the presence of a peptidoglycan cue from a sensitive competitor, a response mediated by the global regulator of cellular competence, ComA. These results experimentally demonstrate a general ecological concept – closely related communities are favoured during competition, due to compatibility in attack and defence mechanisms.

## Abstract (web summary)

Microbial communities employ a variety of strategies to compete against competitors sharing their niche, for instance, by producing antibiotics. This study reveals that antibiotics produced by *Bacillus subtilis* act synergistically to eliminate phylogenetically distinct competitors and are regulated accordingly.

## Introduction

In terrestrial microenvironments, bacteria are present predominantly in architecturally complex and specialized multispecies communities^1^. In many instances, these communities provide beneficial effects to other organisms, e.g., biocontrol agents form biofilms on the surface of plant roots, thereby preventing the growth of bacterial and fungal pathogens^2^. Soil and plant-associated microbial populations survive in this extremely competitive niche by producing a broad arsenal of antibiotics and evolving complex antibiotic-resistance mechanisms^3,4^. Social structure was shown to mediate competition between populations, suggesting a role for antibiosis in shaping cohesive communities^5^. Antibiotic production was shown to be regulated as well as affected by the biological, chemical and physical features of the environment^6–8^.

In *Streptomycetes*, soil bacteria characterized by a complex secondary metabolism, antibiotic production is highly responsive to their social and resource environment, and it was suggested that the identity of the competitor, e.g., competition sensing, might affect antibiotic production^9^. An important step forward is to determine how these social interactions influence competitive outcomes, especially considering that antibiotic production requires resources from the cellular metabolism. Members of *Bacillus subtilis* clade contains several species of Gram-positive, soil dwelling, beneficial bacteria, that employ multiple strategies to compete with neighbor communities^2,10–16^. One major class of antibiotics produced by bacteria are the Non-Ribosomal Peptides (NRPs) and Polyketides (PKS), which are synthesized by large multi-enzyme complexes of non-ribosomal peptide synthetases (NRPSs) and polyketide synthases (PKSs)^2,17,18^. *B. subtilis* contains three different NRP biosynthetic clusters, which are responsible for the biosynthesis of surfactin, bacilysin and plipastatin and a NRP/PKS hybrid biosynthetic cluster responsible for biosynthesis of bacillaene^2,19–22^. In addition to NRPs/PKS antibiotics *B. subtilis* is also capable of producing potent lantibiotic subtilosin^23^ and sublancin, an antimicrobial peptide belonging to the glycocin family^24^.

The most well-characterized NRP is the cyclic lipopeptide surfactin^25^. Surfactin is a small cyclic lipopeptide induced during the development of genetic competence^26,27^ and was suggested to promote horizontal gene transfer^28^. The machinery for surfactin synthesis is encoded by the genes within the *srfAA–AB–AC–AD* operon^29^. Surfactin is a powerful surfactant with antibacterial^30,31^ and antifungal properties^32^ and is composed of an amphipathic, cyclic heptapeptide head group that is interlinked with a hydrophobic β-hydroxy fatty acid tail, comprising 12–16 carbon atoms^33–35^. These features enable the surfactin molecule to interact with, and disrupt the integrity of, cellular membranes^36^.

Bacillaene and dihydrobacillaene^19,37^ are linear antimicrobial macrolides with two amide bonds and are synthesized by the PksC-R clusters mega-complex, which is composed of 13 PKS and three NRPS modules^37^. The expression of *PKS* genes requires the master regulator for biofilm formation, Spo0A, and promotes the competitiveness of biofilm communities^38^.

Bacilysin is a non-ribosomal dipeptide, is composed of the amino acids L-alanine and L-anticapsin, and acts as an antibiotic with activities against a wide range of bacterial and fungal pathogens^39–41^. Its synthesis is controlled by the *bac* operon (*bacABCDE)* and is additionally regulated by other enzymes such as thymidylate synthase, homocysteine methyl transferase and the oligopeptide permeases^42^. This dipeptide undergoes peptidase-mediated proteolysis to release L-anticapsin, which is a competitive inhibitor of glucosamine synthase^39^. This dipeptide can also be produced as chlorotetain, with an unusual chlorine-containing amino acid^43,44^.

Lastly, plipastatin is synthesized non-ribosomally by five fengycin synthetases (*ppsA-E*), that are regulated by DegQ, a master regulator of the transition from a motile cell state to a biofilm-forming state^45^. DegQ is activated by catabolite repression and is under the control of the DegSU two-component system, that controls the formation of the extracellular matrix, sporulation, protease production and motility^46,47^. ComA is a response regulator acting in the two-component regulatory system ComP/ComA involved in genetic competence and surfactin production^48^. It was recently reported that ComA influences the transcription of more than 100 genes^49^, including the genes involved in the biosynthesis of bacillaene^38^ and bacilysin^50^. *B. subtilis* strains were previously shown to distinguish related (kin) and non-related species during swarming^13,14^. The activation of the cell wall stress response was demonstrated during non-kin interactions; however, no single deletion or combination of deletions had a detectable effect on non-kin boundaries^13^. Although the production of antimicrobials was suggested to play a role in the boundaries between non-kin swarms^13^ the role of NRPs/PKS clusters in competition sensing and non-kin recognition was not directly examined.

The production of several potent antibiotic clusters in *B. subtilis*, together with its capacity to distinguish non-kin *B. subtilis* strains allows us to utilize a genetic approach to investigate fundamental questions regarding antibiosis: is there a trade-off between antibiotic biosynthesis and growth? How can bacteria avoid producing costly antibiotics against members of the community that are resistant to their action? To resolve these open questions, we systematically explored NRPs/PKS antibiotics biosynthetic clusters during interactions between related *Bacillus* species.

We found that *Bacillus* species resolve the conflict between antibiotics production and growth by increasing the transcription of NRPs/PKS only when competing against phylogenetically distant species, which are sensitive to this class of antibiotics. In contrast, NRPs/PKS production was not enhanced by the presence of NRPs/PKS-resistant members of the genus. Purified peptidoglycan from competitors (PG), a polymer constituting the outmost layer of the cell wall, was sufficient to induce the expression of antibiotic biosynthesis promoters. Peptidoglycan from sensitive competitors was significantly more efficient in inducing antibiotic production compared with PG of resistant competitors, and their increased production came with a cost manifested in reduced growth. Furthermore, a single transcription factor, ComA, a global regulator that primarily regulates genes associated with genetic competence, was essential to respond to PG from sensitive competitors.

These results demonstrate how the formation of communities composed of closely related species is favored during competition, due to compatibility in antibiotic production and sensitivity. Furthermore, they suggest a mechanism by which microbial populations resolve the conflict between antibiotic production and growth. Our results lead to a fuller understanding of the role of antibiotic regulation in complex habitats and the factors that maintain microbial diversity.

## Results

### *B. subtilis* eliminates competitors by non-ribosomal peptides and polyketides

**Fig. 1:**
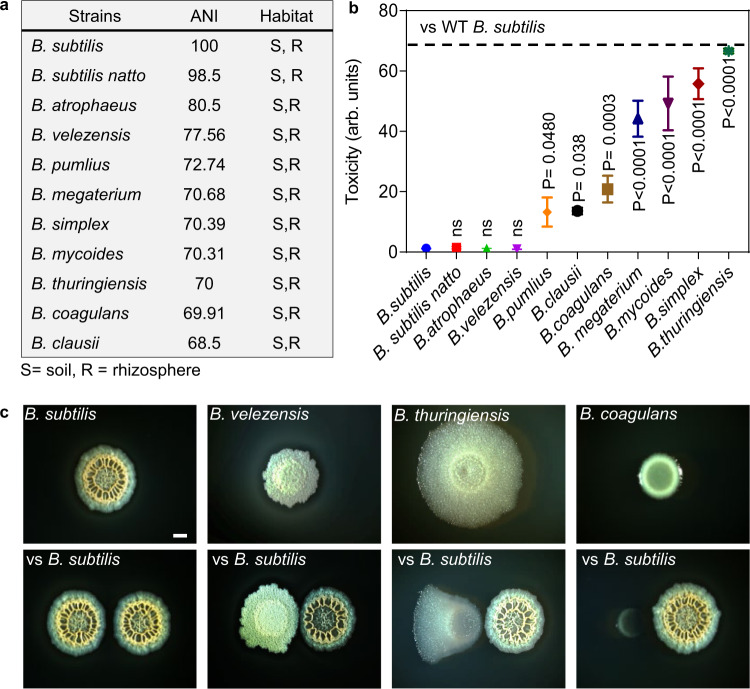
Context dependent toxicity of *B. subtilis* towards competing Bacillus species. **a** Average nucleotide identity (ANI) of indicated *Bacillus* species compared with WT *B. subtilis* and their terrestrial habitats^51,52,97,98^. **b** Toxicity of WT *B. subtilis* towards indicated *Bacilli* was evaluated. Biofilm cells were harvested at 48 h post inoculation and colony-forming units (CFU) were calculated alone, and during co-inoculation. Arbitrary Units (arb. units) for toxicity were determined as the ratio of *Bacilli* CFU in isolation /CFU in competition against WT *B. subtilis*: Colonies of each strain were easily distinguishable. Graphs represent mean ± SD from three independent experiments (*n* = 9). Statistical analysis was performed using one-way ANOVA followed by Tukey’s multiple comparison post hoc testing. *P* < 0.05 was considered statistically significant. Significant differences between the toxicity towards WT *B. subtilis* and toxicity towards indicated *Bacilli*, when competed against WT *B. subtilis* are shown by their respective *p* values. Dashed Line: The maximal toxicity exhibited by WT *B. subtilis*. **c** Shown is the indicated biofilm colony grown in isolation versus indicated biofilm colony grown in proximity to WT *B. subtilis* colony (below). Colony biofilms were inoculated at 0.4 cm apart and grown on B4 medium at 30 °C. Images are from representative experiment performed in triplicates out of three independent experiments. Biofilms colonies were imaged at 48 h post inoculation. Scale bar = 1 mm. Source data are provided as a Source Data file.

To determine how NRPs and PKS biosynthetic clusters interact during microbial competition, we first examined the competition between *B. subtilis* NCIB 3610 (Wild Type, WT) and ten representative soil dwelling *Bacilli*^51,52^ spread over various phylogenetic distances (Fig. 1a). The master regulator Spo0A is activated by phosphorylation via a multicomponent phosphorelay to induce biofilm formation^53^ and antibiotic production in *B. subtilis*^38,50,54^. We performed the experiment on a biofilm medium B4^55,56^ as this rich medium induces robust biofilm formation of *B. subtilis*^55,57^ and allows efficient growth of multiple soil isolates^31^.

Phylogenetic distance between WT *B. subtilis* and its competitors was quantified using the nucleotide level genomic similarity between the coding regions of two genomes, expressed as ANI (Average Nucleotide Identity)^58^. A competition assay was developed to test the potential interactions between WT *B. subtilis* and its competitors. The degree of toxicity exhibited by WT *B. subtilis* towards competing *Bacilli* was calculated by dividing the culturable cell counts (as judged by colony-forming units, CFU) of biofilms grown in isolation by CFU of biofilms competing against WT *B. subtilis* (Fig. 1b).

Our results showed that members of the *subtilis* clade (resistant members) – WT *B. subtilis*, *B. subtilis natto, B. velezensis* and *B. atrophaeus* – coexisted well with *B. subtilis* during competition. In contrast, phylogenetically distinct species (sensitive members) – *B. megaterium, B. mycoides, B. simplex, B. thuringiensis, B. coagulans and B. clausii* – were eliminated by WT *B. subtilis*. The maximum degree of toxicity exhibited by WT *B. subtilis* against sensitive *Bacilli* was ≃67 fold (Fig. 1b). Results were consistent with alterations in colony morphologies and growth during interactions: *B. megaterium, B. mycoides, B. simplex, B. thuringiensis, B. coagulans and B. clausii* struggled to grow in proximity to WT *B. subtilis*, while members of the same clade (resistant members) were largely unaffected by this interaction (Fig. 1b, c and Supplementary Fig. 1). For all interactions, WT *B*. subtilis itself was largely unaffected (Supplementary Fig. 2).

**Fig. 2:**
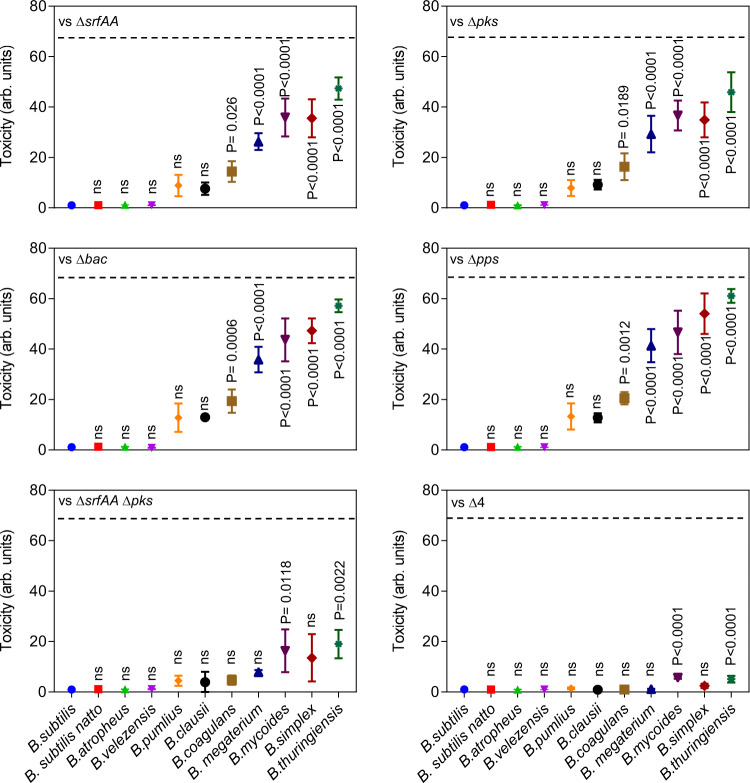
The contribution of non-ribosomal peptides and polyketides to within genus competition. The toxicity of WT *B. subtilis* strain harboring NRP single mutants ∆*srfAA* (surfactin), ∆*pks* (bacillaene), ∆*bac* (bacilysin), ∆*pps* (plipastatin), a double mutant for ∆*srfAA* and ∆*pks*, and a quadruple mutant for all NRPs/PKS operons ∆*srfAA*, ∆*pks*, ∆*bac and* ∆*pps* (∆4) towards indicated *Bacilli* was evaluated. Biofilm cells were harvested at 48 h post inoculation and colony-forming units (CFU) were calculated alone, and during co-inoculation. Arbitrary Units (arb. units) for toxicity were determined as the ratio of *Bacilli* CFU in isolation /CFU in competition against WT *B. subtilis*: Graphs represent mean ± SD from three independent experiments (*n* = 9). Statistical analysis was performed using one-way ANOVA followed by Tukey’s multiple comparison post hoc testing. *P* < 0.05 was considered statistically significant. Significant differences between the toxicity towards WT *B. subtilis* and toxicity towards indicated *Bacilli*, when competed against the indicated NRPs/PKS mutants are shown by their respective *p* values. Dashed Line: The toxicity exhibited by WT *B. subtilis*. Source data are provided as a Source Data file.

We studied the contribution of four different NRPs/PKS gene clusters to the antibiosis: *srfAA-srfAD, pksC-pksR, bacA-ywfG and ppsA-ppsE*; responsible for the biosynthesis of surfactin, bacillaene, bacilysin and plipastatin, respectively. The degree of toxicity exhibited by WT *B. subtilis* (maximum 67 fold) was used as a reference. When *Bacillus* species were competed against a mutant lacking either *srfAA (*Δ*srfAA)* or *pksC-pksR* (Δ*pks*), the maximal toxicity was reduced to 47 and 45-fold respectively (Fig. 2). In contrast, the toxicity of strains lacking *bacC* (Δ*bac*) *and ppsA* (Δ*pps*) was similar to that of the WT *B. subtilis* strain (Fig. 2). Next, we examined combinations of double deletions. Only a double mutant lacking surfactin and bacillaene biosynthesis (Δ*srfAA*, Δ*pks*) was clearly compromised in its toxicity compared to either of the single deletion mutants (Fig. 2 and Supplementary Fig. 3). Furthermore, a quadruple mutant lacking all NRPs/PKS biosynthetic clusters (Δ4) lost the vast majority of toxicity towards sensitive members (Fig. 2). The residual toxicity (7.5%, ≃5 folds) could be attributed to additional antibiotics and lantibiotics produced by WT *B. subtilis*. An overall effect of deletion of NRPs/PKS gene clusters on toxicity on individual *Bacilli* is provided in Supplementary Table 1.

Multiple agents synergize if they are more effective in combination than one would expect by adding their individual effects. To determine if NRPs/PKS biosynthetic clusters interact, their observed combined effect was compared with a reference that predicts how individual effects would add up independently. Relying on the genetic analysis of antibiosis described in (Fig. 1b, 2 and Supplementary Fig. 3), we compared the *observed* combined toxicity of multiple antibiotics to the one *expected* from the Bliss independence model^59^.

**Fig. 3:**
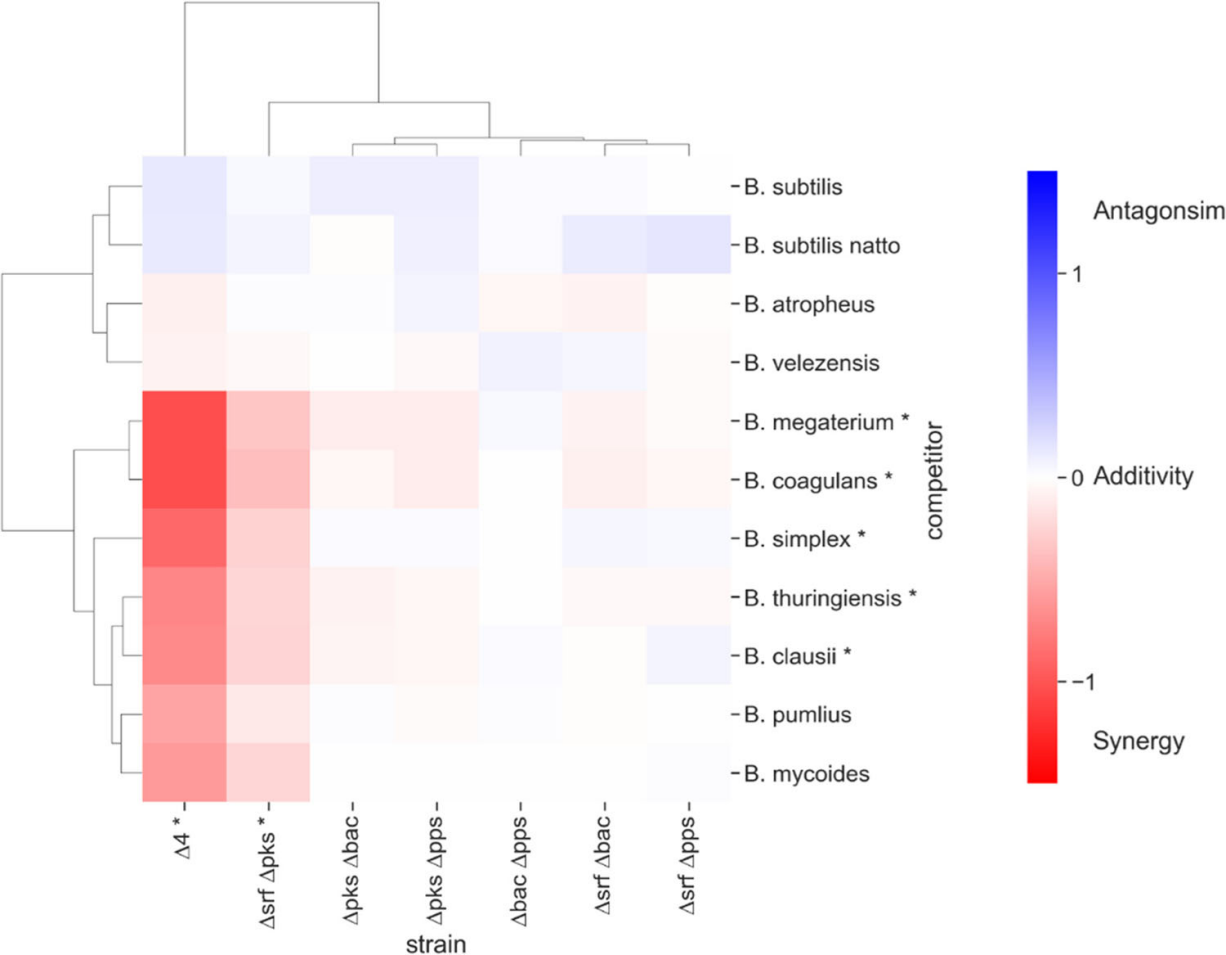
NRPs/PKS act synergistically to eliminate sensitive *Bacilli*. Clustered heatmap showing the calculated interaction scores between NRPs/PKS antibiotics. Interaction score =0 represents no interaction, >0 represents antagonistic interaction and <0 represents synergistic interactions. Each interaction score is the average of scores calculated from three independent experiments. Asterisks next to an antibiotic combination (competitor’s name) indicate that the interaction scores across the corresponding column (row) significantly deviated from 0 (*p* value < 0.05). *P* values were calculated for each combination of antibiotics and competitor using a two-tailed one-sample *t*-test, and the *p* values for all competitor (antibiotics combinations) were combined using Fisher’s methods (shown in Supplementary Data 1). Rows and columns of the heatmap were hierarchically clustered based on the interaction score values, using the clustermap function in Python’s seaborn library with default parameters. Source data are provided as a Source Data file.

Interaction scores of 0 indicated additivity (antibiotics that act independently), scores greater than 0 represented antagonism and scores less than 0 indicated synergy. No significant antagonism was identified in any of the antibiotic combinations studied. In addition, none of the pairs showed any synergy against “resistant” *Bacillus* species. In contrast, the pair bacillaene - surfactin acted synergistically against sensitive *Bacilli*. When the effects of all four antibiotics were combined, synergism against sensitive strains increased significantly (Fig. 3).

**Fig. 4:**
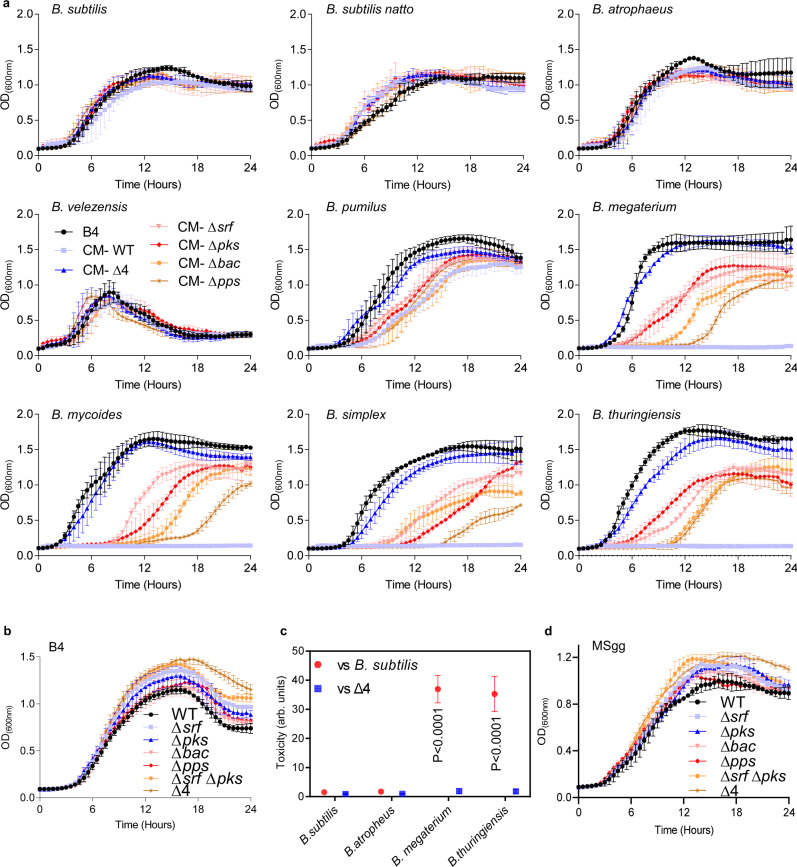
The effect of non-ribosomal peptides and polyketides on toxicity and growth. **a** Planktonic growth of the indicated species was monitored either in B4 medium (control) or B4 medium supplemented with CM (15% v/v) of WT *B. subtilis* or its NRP single mutants ∆*srfAA* (surfactin), ∆*pks* (bacillaene), ∆*bac* (bacilysin), ∆*pps* (plipastatin), a double mutant for ∆*srfAA* and ∆*pks*, and a quadruple mutant for all NRPs/PKS operons ∆*srfAA*, ∆*pks*, ∆*bac and* ∆*pps* (∆4). Graphs represent mean ± SD from three independent experiments (*n* = 9). Statistical analysis was performed using two-way ANOVA followed by Dunnett’s multiple comparison test. *P* < 0.05 was considered statistically significant. **b** Planktonic growth of WT *B. subtilis* and the indicated mutants: was monitored in B4 medium. Graphs represent mean ± SD from three independent experiments (*n* = 9). Statistical analysis was performed using two-way ANOVA followed by Dunnett’s multiple comparison test. *P* < 0.05 was considered statistically significant. **c** Toxicity of WT *B. subtilis* and its quadruple mutant (∆4) towards indicated *Bacilli* was evaluated in MSgg medium. Arbitrary Units [AU] for toxicity were determined as the ratio of *Bacilli* CFU in isolation /CFU in competition against WT *B. subtilis*: Biofilm cells were harvested at 48 h post inoculation and colony-forming units (CFU) were calculated alone, and during co-inoculation. Graphs represent mean ± SD from three independent experiments (*n* = 9). Statistical analysis was performed using one-way ANOVA followed by Tukey’s multiple comparison post hoc testing. *P* < 0.05 was considered statistically significant. Significant differences between the toxicity of WT *B. subtilis* and toxicity of indicated *Bacilli* are shown by their respective *p* values. **d** Planktonic growth of the WT *B. subtilis* and the indicated mutants was monitored in MSgg medium. Graphs represent mean ± SD from three independent experiments (*n* = 9). Statistical analysis was performed using two-way ANOVA followed by Dunnett’s multiple comparison test. *P* < 0.05 was considered statistically significant. *P* values at different time points in panels **a**, **b** and **d** are shown in the Supplementary Data 1. Source data are provided as a Source Data file.

When *Bacilli* were treated with conditioned medium from WT *B. subtilis*, sensitive members failed to grow. This was not the case when sensitive members were treated with conditioned media from NRPs/PKS deletion mutants. This suggesting that toxicity of the CM was due to the presence of secreted antibiotics (Fig. 4a). Similarly, to their contribution during interspecies interaction on plate, conditioned media containing surfactin and bacillaene contributed most of the toxicity towards sensitive community members, while maintaining minimal or no toxicity towards resistant strains (Fig. 4a and Supplementary Fig. 4).

### NRPs/PKS biosynthetic clusters expression is induced by sensitive competitors to reduce the burden on microbial growth

In order to understand whether production of NRPs and PKS clusters affects the growth of WT *B. subtilis*, we monitored growth rates of WT *B. subtilis* and its NRPs deletion mutants. Indeed, the growth of parental strain lower than ∆*srf* and ∆*pks* single mutants – and especially as compared to that of the quadruple mutant (Fig. 4b). To assess the role of nutrients availability on antibiotic production and its cost, we competed *Bacillus s*pecies capable of growing on a defined biofilm medium MSgg, where glycerol serves as a carbon source (Fig. 4c). The elimination of competitors again relied on the NRPs and PKS clusters. Fitness cost for antibiotic production was also evident in a defined biofilm medium MSgg (Fig. 4d), indicating that the cost for antibiotic production is robust, although the cost of a single antibiotic is reduced in a defined medium. NRPs and PKS gene clusters encode large complexes composed of many protein subunits. Considering the burden of their production (Fig. 4b, d), we wondered whether these operons are differentially transcribed during interaction with the resistant and the sensitive competitors.

**Fig. 5:**
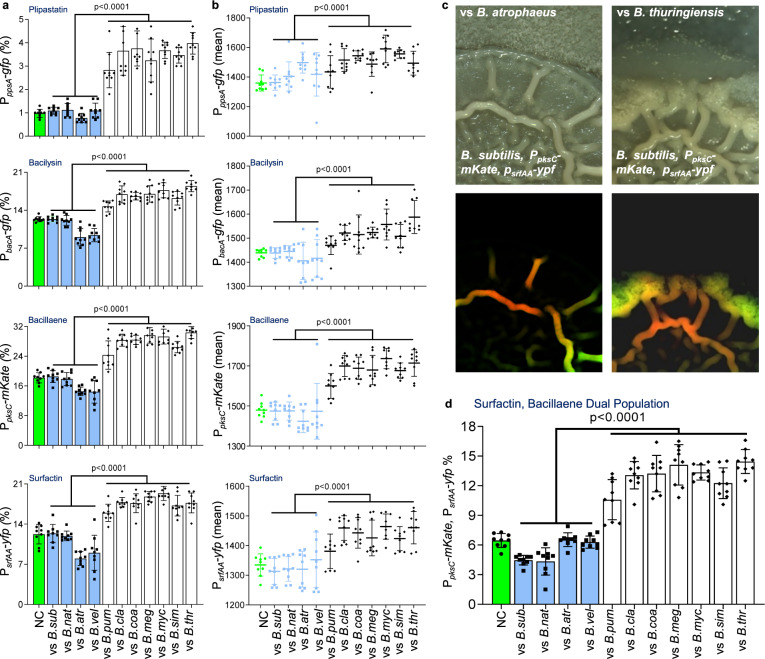
Sensitive and resistant *Bacillus* competitors regulate the transcription from the promoters of NRPs/PKS biosynthetic clusters. **a**, **b** A dual reporter of WT *B. subtilis* strain harboring P_*srfAA*_*-yfp* (surfactin), P_*pksC*_*-mKate* (bacillaene), and single reporters of P_*bacA*_*-gfp* (bacilysin) and P_*ppsA*_*-gfp* (plipastatin) were analyzed either alone (NC) or in competition against indicated *Bacilli* using flow cytometry. Colonies were grown on B4 medium and incubated at 30 °C. Data were collected from 24 h post inoculation, 100,000 cells were counted. *Y*-axis represents (**a**) the % of cells expressing the reporters, graphs represent mean ± SD from three independent experiments (*n* = 9). **b** Graphs showing intensity of the fluorescent population, data is presented as mean (solid line) ± SD from three independent experiments (*n* = 9). Statistical analysis was performed between resistant and sensitive members using a using unpaired two-tailed *t*-test with Welch’s correction. *P* < 0.05 was considered statistically significant. **c** A dual reporter pf WT *B. subtilis* strain harboring both P_*srfAA*_*-yfp* (surfactin) and P_*pks*_*-mKate* (bacillaene) was competed against indicated *Bacilli* and imaged using Zeiss stereomicroscope at 44-x magnification. Upper panel (Phase), Bottom panel (Fluorescence). An increase in the fluorescence of dual population was observed when competed against sensitive member– *B. thuringiensis*. **d** A dual reporter of WT *B. subtilis* strain harboring of P_*srfAA*_*-yfp* (surfactin), P_*pksC*_*-mKate* (bacillaene), was analyzed either alone (NC) or in competition against indicated *Bacilli* using flow cytometry. Colonies were grown on B4 medium and incubated at 30 °C. Data were collected from 24 h post inoculation; Y-axis represents the % of cells expressing both surfactin and bacillaene reporters, 100,000 cells were counted. Graphs represent mean ± SD from three independent experiments (*n* = 9). Statistical analysis was performed between resistant and sensitive members using a using unpaired two-tailed *t*-test with Welch’s correction. *P* < 0.05 was considered statistically significant. *P* values comparing the effect on each fluorescent reporter when competing against different *Bacilli* in panels **a**, **b** and **d** are shown in the Supplementary Data 1. Source data are provided as a Source Data file.

Using flow cytometry, we examined the expression of transcriptional reporters P_*srfAA*_*-yfp*, P_*pksC*_*-mKate*, P_*bacA*_*-gfp* and P_*ppsA*_*-gfp* in biofilm colonies to ask whether interspecies competition would have an impact on their expression. The number of cells expressing each antibiotic reporter and their respective mean intensity of fluorescence remained mostly constant or even decreased while interacting with resistant *subtilis* clade members. On the other hand, both increased during competition against sensitive *Bacillus* species (Fig. 5a, b), linking transcriptional activity of these promoters and the potential fitness benefits of antibiotics production.

An additional layer of regulation on NRPs/PKS production could be the presence within the cells expressing of one or more biosynthetic clusters. To further study the division of labor at single cell level, a dual transcriptional reporter, harboring P_*srfAA*_*-yfp*, P_*pksC*_*-mKate*, was constructed and the expression of these alleles was assessed simultaneously during growth in isolation, and during competition. A distinct population expressing from both surfactin and bacillaene promoters was detected (Fig. 5c, d). Furthermore, we observed a two-fold increase in the population expressing both surfactin and bacillaene reporters in the presence of sensitive *Bacillus* species, (Fig. 5c, d). Consistent with the notion that the induction of antibiotics is mediated by a secreted signal, the induction initiated in the area of the colony in proximity to the competitor (Supplementary Fig. 5).

### Peptidoglycan derived from sensitive competitors induces recognition and antibiotic production

We asked whether NRPs and PKS induction occurs by specifically sensing a secreted signal from sensitive competitors. Therefore, we used WT *B. subtilis* strains harboring the luciferase reporters P_*pksC*_*-lux* (bacillaene) and P_*srfAA*_*-lux* (surfactin) for real-time measurements of their expression. This analysis allowed us to accurately distinguish between signals and mutant strains that alter the dynamics of the activity from the promoter^60,61^.

**Fig. 6:**
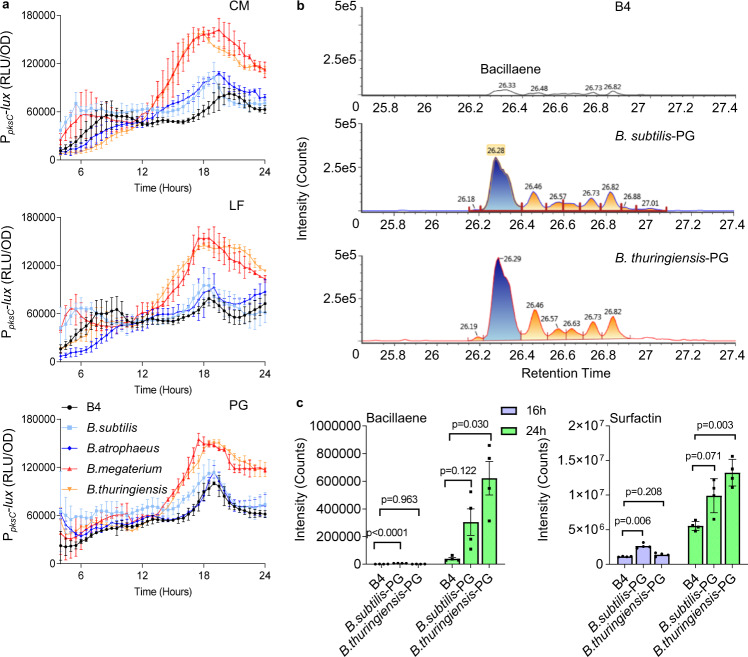
Sensing peptidoglycan regulates the transcription from the promoters of NRPs and PKS biosynthetic clusters. **a** Analysis of the luciferase activity in a WT *B. subtilis* strain harboring P_*pksC*_*-lux* (bacillaene) reporter. Luminescence was monitored in B4 medium (No Treatment), and B4 medium supplemented with 15% v/v of the conditioned medium (CM), or an equivalent amount of the conditioned medium fractionated to generate LF (>3 kDa) and peptidoglycan PG (100 ng/µl) from the indicated species. Graphs represent mean ± SD from three independent experiments (*n* = 9). Statistical analysis was performed using two-way ANOVA followed by Dunnett’s multiple comparison test. *P* < 0.05 was considered statistically significant. **b** Representative peaks from liquid chromatography-mass spectrometry analysis of bacillaene from the WT *B. subtilis* grown in untreated B4 medium (control) or in a B4 medium treated with purified PG (100 ng/µl) of indicated species at 24 h. **c** Liquid chromatography-mass spectrometry analysis of surfactin isoform 1 and bacillaene from WT *B. subtilis* grown in untreated B4 medium (control), and B4 medium supplemented with PG (100 ng/µl) from the indicated species. Supernatant was extracted from the samples at 16 h and 24 h using HCl treatment. Graphs represent mean ± SEM from four biological repeats (*n* = 4). Statistical analysis was performed using Brown–Forsthye and Welch’s ANOVA with Dunnett’s T3 multiple comparisons test. *P* < 0.05 was considered statistically significant. *P* values comparing the effect on each fluorescent reporter when competing against different *Bacilli* in panel a are shown in the Supplementary Data 1. Source data are provided as a Source Data file.

As shown (Fig. 6a and Supplementary Fig. 8), conditioned medium from sensitive competitors were sufficient to induce the transcription of both surfactin and bacillaene. In contrast, the conditioned medium of resistant competitors minimally induced surfactin and bacillaene expression. Consistent with the notion that enhanced production comes with a metabolic cost, the exposure to inducing conditioned medium also increased the growth defect in WT *B. subtilis*. In contrast, inducing conditioned medium did not have a significant effect on the growth of quadruple mutant (Δ4) incapable of NRPs/PKS production (Supplementary Fig. 6).

To identify the secreted factor which serves to activate the NRPs/PKS transcription, we first separated the conditioned medium of sensitive and resistant competitors by size. A fraction >3Kda was sufficient to activate the transcription from both *srfAA* and *pksC* promoters (Fig. 6a and Supplementary Fig. 7 and 8) while the smaller fraction was inert.

Therefore, the sensitive discrimination mechanism was not mediated by a small molecule but most likely by either a protein or an alternate polymer. To exclude the involvement of secreted proteins and peptides, we treated the conditioned medium with proteinase K, a potent protease. As shown, the treatment did not reduce the potency of the conditioned medium (Supplementary Fig. 9). Similarly, purified exopolysaccharides (EPS) (Supplementary Fig. 10) failed to induce the transcription from *srfAA* and *pks* promoters. Similarly, gDNA did not induce transcription from *srfAA* and *pks* promoters (Supplementary Fig. 11). These results indicated that neither a small molecule pheromone, EPS, proteins nor extracellular DNA mediated sensitive members’ recognition.

Peptidoglycan fragments have also been reported to function as signaling molecules that trigger adaptive responses. For instance, low concentration of muramopeptides released by actively growing WT *B. subtilis* cells act as a potent germinant of *B. subtilis* spores^62^. Furthermore, PG was detected in the agar surrounding an inducing competitor, *B. thuringiensis* (Supplementary Fig. 12). Therefore, we tested whether pure peptidoglycan is sufficient to trigger the recognition of competing *Bacillus* species. Indeed, purified peptidoglycan of *B. megaterium* and *B thuringiensis* was more significant in inducting the expression of both surfactin and bacillaene as compared to PG of WT *B. subtilis and B. atrophaeus*, in both rich medium (Fig. 6a and Supplementary Fig. 13) and defined medium (Supplementary Fig. 14). Furthermore, our results showed that PG was also sufficient to induce the secretion of both surfactin and bacillaene to the growth media, with PG from sensitive competitors being a significantly more potent inducer than PG from resistant competitors (Fig. 6b, c, Supplementary Figs. 15 and 16 and Supplementary Table 2).

### Induction of antibiotic production by PG is correlated with a fitness cost

**Fig. 7:**
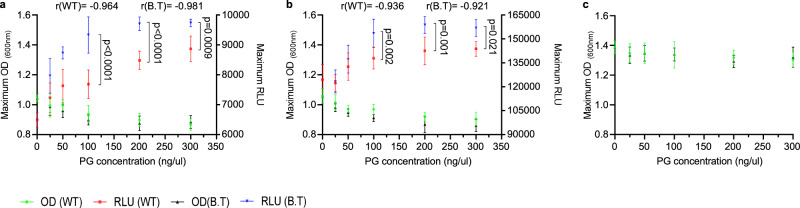
Peptidoglycan regulates the transcription from the promoters of NRPs/PKS biosynthetic clusters in the cost of reduced growth. **a** Correlation between maximal recorded growth (OD) and maximal recorded luciferase activity (RLU) in a WT *B. subtilis* strain harboring P_*srfAA*_*-lux* (surfactin) reporter. Growth and luminescence were monitored in B4 medium (No Treatment), and B4 medium supplemented with different PG concentrations (25–300 ng/µl) from indicated species (WT – *B. subtilis*, BT- *B. thuringiensis*). Graphs represent mean ± SD from three independent experiments (*n* = 9). Correlation between growth and luciferase activity of was calculated at different concentrations of PG using Pearson correlation coefficient (r). Statistical analysis was performed using two-way ANOVA followed by Tukey’s multiple comparison post hoc testing. *P* < 0.05 was considered statistically significant. Significant differences are shown by their respective *p* values. **b** Correlation between maximal recorded growth (OD) and maximal recorded luciferase activity (RLU) in a WT *B. subtilis* strain harboring P_*pksC*_*-lux* (bacillaene) reporter. Growth and luminescence were monitored in B4 medium (No Treatment), and B4 medium supplemented with different PG concentrations (25–300 ng/µl) from indicated species (WT – *B. subtilis*, BT- *B. thuringiensis*). Graphs represent mean ± SD from three independent experiments (*n* = 9). Correlation between growth and luciferase activity at different concentrations of PG was calculated using Pearson correlation coefficient (r). Statistical analysis was performed using two-way ANOVA followed by Tukey’s multiple comparison post hoc testing. *P* < 0.05 was considered statistically significant. Significant differences are shown by their respective *p* values. **c** Analysis of the maximal recorded growth (OD) of WT *B. subtilis* quadruple mutant (∆4) in B4 medium (No Treatment), and B4 medium supplemented with different PG concentrations (25- 300 ng/µl) from indicated species (WT – *B. subtilis*, BT- *B. thuringiensis*). Statistical analysis was performed using Two-way ANOVA followed by Tukey’s multiple comparison post hoc testing. *P* < 0.05 was considered statistically significant. No significant difference were observed. Source data are provided as a Source Data file.

To assess the response to PG from sensitive and resistant competitors, we performed a dose-response measurement of the expression from the biosynthetic clusters of surfactin and bacillaene. The advantage of using the cue for antibiotic production, rather than the un-purified conditioned medium was the absence of the secreted antibiotics from the inducing solution. As shown (Fig. 7a, b and Supplementary Figs. 17–20), expression from the *srf* and *pks* reporters exhibited dose-response relationships with PG from both sensitive and resistant competitors. This result was consistent with the LC-MS data, and suggests that PG acts as a signal for antibiotic production regardless of the identity of its producer. However, the source of the PG significantly alters the intensity of the expression (*P* < 0.05).

PG from resistant competitors was a more potent signal for the activation of transcription from the *pks* and *srf* promoters, when provided at the same concentrations. This quantitative effect was consistent with the LC-MS results for antibiotic production (Fig. 6b, c), collectively verifying that variations in the chemistry of PG also contributes to the intensity of the signal.

Using this quantitative simplified system, we continuously assessed the correlation between antibiotic production and growth. The induced expression of both surfactin and bacillaene was indeed correlated with a significant reduction in cell growth. This reduction was indeed due to the cost of antibiotic production, as we could not observe it in a quadruple mutant (Fig. 7c and Supplementary Fig. 21).

### Peptidoglycan acts upstream to ComA to induce sensitive competitors recognition by antibiotic production and competence

In *B. subtilis*, canonical response to secreted PG is mediated by the PG sensors PrkC kinase^63^, recognizing muramopeptides, MurP^62^ for Muramic acid and NagP^62^ for N-Acetylglucosamine. However, deleting those genes had no effect on the basal levels or the induction of the transcription from NRPs/PKS promoters in the presence of resistant competitors (Supplementary Fig. 22).

**Fig. 8:**
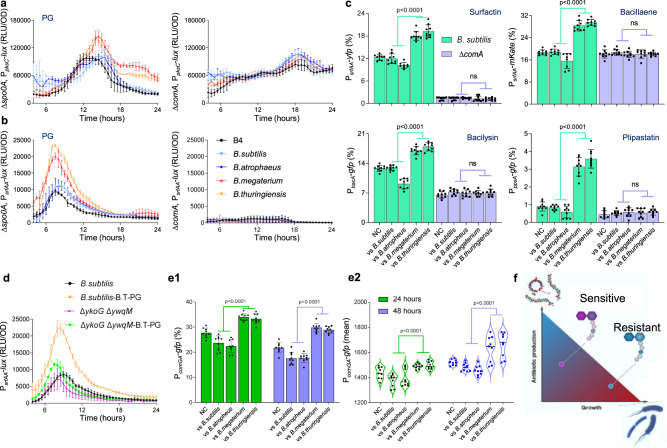
ComA and PG receptors mediate the recognition of sensitive competitors. **a**, **b** Analysis of the luciferase activity in WT *B. subtilis* strain harboring P_*pksC*_*-lux* (bacillaene) and P_*srfAA*_*-lux* (surfactin) reporters and their indicated mutants. Luminescence was monitored in B4 medium (No Treatment), and B4 medium supplemented with PG (100 ng/µl) from the indicated species. Graphs represent mean ± SD from three independent experiments (*n* = 9). **c** WT *B. subtilis* strains harboring P_*srfAA*_*-yfp* (surfactin), P_*pksC*_*-mKate* (bacillaene), P_*bacA*_*-gfp* (bacilysin) and P_*ppsA*_*-gfp* (plipastatin) reporters and their ∆*comA* mutants were grown either alone (NC) or in competition against indicated *Bacilli*, and their cells were analyzed using flow cytometry. Graphs represent mean ± SD from three independent experiments (*n* = 9). **d** Analysis of the luciferase activity in WT *B. subtilis* strain harboring P_*srfAA*_*-lux* (surfactin) reporter and its ∆*ykoG* ∆*ywqM* mutant. Luminescence was monitored in B4 medium (No Treatment), and B4 medium supplemented with PG (100 ng/µl) from *B.thurngiensis* (B.T-PG). Graphs represent mean ± SD from three independent experiments (*n* = 9). **e** A WT *B. subtilis* strain harboring P_*comGA*_*-gfp* reporter were analyzed either alone (NC) or in competition against indicated *Bacilli* using flow cytometry.; *Y*-axis represents (e1) the % of cells expressing the reporters (e2) Violin plots showing intensity of the fluorescent population, data is presented as median (solid line) with 25th and 75th percentile (dashed line) from three independent experiments (*n* = 9). **f** A conceptual model of the conflict between growth and antibiotic production, and how it is resolved by the PG mediated regulation of antibiotic production. For panels **a**, **b** and **d** statistical analysis was performed using two-way ANOVA followed by Dunnett’s multiple comparison test. For panels **c** and **e** statistical analysis was performed between resistant and sensitive members using Brown–Forsthye and Welch’s ANOVA with Dunnett’s T3 multiple comparisons test. *P* < 0.05 was considered statistically significant. *P* values at different time points in panels **a**, **b** and **d** are shown in the Supplementary Data 1. Source data are provided as a Source Data file.

Surfactin and bacilysin are both regulated by the two-component regulatory system ComP/ComA involved in a major quorum response pathway that regulates the development of genetic competence^2^. We found that deletion of ComA was sufficient to eliminate recognition of competitors by the surfactin and bacillaene promoters. Specifically, for surfactin expression of the deletion of ComA eliminated both basal expression and competitors’ recognition, and for bacillaene the deletion eliminated specifically resistance recognition (Fig. 8a–c and Supplementary Fig. 23). In contrast, deletions of Spo0A and DegU, the master regulators of sporulation and biofilm formation^64^ did not specifically eliminate the response to peptidoglycan. CodY regulated the basal expression levels of from a bacillaene biosynthesis promoter. However, a *codY* mutant was capable to a significant degree of sensitive competitors’ recognition as judged by the induction of surfactin promoter (Supplementary Fig. 23).

*B. subtilis* genome has several homologs for known PG receptors^65^. Putative PG sensors were screened for the activity from *pks* and *srf* promoters. Our results indicated that YkoGH (a two-component system regulating LTA glycosylation^66^), and YwqM (a transcription factor from the LysR family with some homology with AmpR^62^) are involved in antibiotics transcriptional regulation: Although a deletion of a single homolog was insufficient to eliminate PG recognition, a combination of both putative receptors was sufficient to significantly reduce competitors’ recognition in competing colonies (Fig. 8d and Supplementary Figs. 24 and 25). Collectively, these results suggest that the ComA pathway is acting jointly with the putative PG sensory machinery (YwqM and YkoGH) to fine-tune antibiotic production.

To assess directly whether PG is a regulator of genetic competence, we explored the expression of ComG, a main regulator of the competence translocon in the presence and absence of PG. Indeed our sensitive competitors also induced the expression of genetic competence as judged by the expression of ComGA (Fig. 8e).

## Discussion

Microbial populations in natural environments secrete various metabolites, such as antibiotics, digestive enzymes, quorum sensing inhibitors, low molecular weight compounds like hydrogen peroxide^10,20–23^ etc. The primary model of our study is the plant biocontrol agent and probiotic bacterium *B. subtilis*^2,67^. Various wild type strains of *B. subtilis* and related species isolated from the soil are known to defend their host from diverse fungal and bacterial pathogens^25,68^. These probiotic properties are largely mediated by the production of non-ribosomal peptides^69,70^.

In *B. subtilis*, a boundary is formed between kin and non-kin strains during swarming^15^ and this self-recognition was shown to involve contact-dependent inhibition^13^ while in *Proteus mirabilis* self-recognition involved type VI secretion systems^71,72^. It was previously hypothesized that direct cell damage (interference competition) activates bacteriocins and antibiotics^8^. Consistently, antimicrobials were also suggested to play a role in stabilizing communities composed of kin strains in *B. subtilis*^13^. However, no specific antibiotic was sufficient for boundary formation between related and unrelated strains and the mechanism for kin recognition remained to be determined.

Previous studies demonstrated that during microbial competition, numerous soil bacteria utilize NRPs/PKS antibiotics to ward off the invading microbes and thus protect their niche^9,10,24,25^. Although molecular mechanisms of synthesis of the surfactin, bacillaene, bacilysin and plipastatin are largely resolved^73^ it remains unknown whether these antibiotics exert their action in an additive or synergistic manner. Furthermore, bacteria that produce these antibiotics are subject to a fitness cost lowering their growth rate. Therefore, we used a genetically manipulatable antibiotic producer to study how different antibiotics interact, and to understand their regulation in light of microbial competition and the conflict between antibiotic production and growth.

We found that the production of NRPs/PKS antibiotics was specifically activated during competition with sensitive species. Our results demonstrated that NRPs/PKS antibiotics act as synergistic pairs (surfactin and bacillaene)with plipastatin and bacilysin) during interspecies competition. All antibiotics toxicity was specific towards competing species phylogenetically distant from the *Bacillus subtilis* clade, but sharing the same terrestrial habitats: soil and plant microbiomes.

We therefore asked whether the expression of these biosynthetic clusters results in a deleterious effect on their producers’ growth in isolation. Indeed, production and further induced production of antibiotics came with a clear cost on the bacterial growth. This trade-off generates selective pressure on the regulation of antibiotics promoters (Figs. 7 and 8f) as antibiotic production becomes deleterious when encountering resistant community members.

Interestingly, our results indicate that CodY, the master regulator of branched-chain amino acids metabolism^74^, is a contributor to the basal level of antibiotic production. This regulation is consistent with a nutrient sensing or catabolite repression on antibiotic production, also observed in *S. coelicolor*^75^.

Using transcriptional reporters, we showed that *B. subtilis* increased the transcription of NRPs/PKS antibiotics only when sensing a specific secreted signal from sensitive species. This suggests that *B. subtilis* can discriminate sensitive from resistant, and reduce the burden of producing antibiotics when encountering resistant clade members. Cell communication and metabolic exchange are essential parts of interspecies competition and lead to the regulation of various molecular elements^26,27^.

An essential feature of *B. subtilis* and Gram-positive bacteria in general is their cell wall. It counteracts the high intracellular osmotic pressure, determines the cell morphology, and serves as both a diffusion barrier and a first layer of defence against environmental threats^76,77^. Fragments of peptidoglycan, a mesh-like structure in the envelope, were shown to be sensed to induce the germination of spores^63^, virulence genes and pyocyanin production of the human pathogen *P. aeruginosa*^78,79^, repress extracellular matrix genes in pathogenic *E. coli*^80^ and induce antibiotic-resistance genes^81^. Furthermore, the soil bacterium *Streptomyces coelicolor* induces the production of antimicrobials following perception of NAG from similar species^82^ Notably, peptidoglycan inducing fragments are quite diverse, and include the stem peptides, N-acetylglucosamine and several other peptidoglycan components^62^. Here, we report on a complementary role for PG recognition: distinguishing sensitive and resistant phylum members competing for the same niche. The normal, unmodified glycan strands of bacterial peptidoglycan consist of alternating residues of β-1,4-linked N-acetylmuramic acid and N-acetylglucosamine. Glycan strands become differentially modified in different species by enzymes responsible for the N-deacetylation, N-glycolylation and O-acetylation of the glycan strands^83^. The composition of the interpeptide bridge also differs between different species^83,84^ making PG fragments an appealing source of information regarding potential competitors’ identity. We cannot exclude that the disintegration of sensitive competitors during competition also serves as a non-specific cue, as PG from resistant competitors also induced NRPs and PKS expression. However, PG from sensitive competitors induced antibiotics production significantly more than PG from resistant competitors at the same concentration, indicating some specificity for the signal. Monitoring the dose-response to PG revealed that the increased induction of NRPs and PKS (Fig. 7) came with an increased growth defect, we suggest that the PG cue acts to minimize the cost for antibiotic production.

In *S. coelicolor* induction of antibiotic production was strongly predicted by phylogenetic distance, with closely related strains more likely to inhibit each other. Previous studies also identified a correlation between phylogenetic distance and inhibition in *Streptomyces*^5,9,85^. In our work, *Bacillus* species which were phylogenetically remote were more likely to be inhibited by *B. subtillis*. This result could be due to conserved resistance genes, frequently adhering or co-evolving with the adjacent antibiotic production genes. Consistently, an analysis of the bacillaene and surfactin production genes revealed their presence in all resistant *Bacillus species* with the exception of *B. subtilis natto* (Supplementary Fig. 26). While the PKS operon was mostly absent from *B. subtilis natto*, gene *PksA* was conserved in its genome (98% conservation, 97% coverage), raising the possibility that it may function as a PKS resistance gene. This hypothesis is consistent with the fact that PksA is a TetR repressor^38^,and putatively belong to a family of proteins that are known to be associated with antibiotic resistance^86^.

Interestingly, *Streptomycetes* increase antibiotic production when grown with susceptible competitors, without assessing the impact of whether or not the competitor could reciprocally inhibit the focal strain. Furthermore, the sensing of sensitivity is not driven by cellular damage caused by the second strain, as specifically predicted by the competition sensing hypothesis^8^. Instead, and consistently with our results, these results suggest that soil bacteria are more likely to induce antibiotic production in response to cues that are correlated with phylogenetic distance, rather than direct harm itself^9^. However, our dose-response analysis may suggest that this specificity is complimentary to the non-specific recognition of cell envelope cleavage and therefore cell damage.

As in Gram-positive bacteria, PG can serve as both an indirect indicator of cell lysis and as a specific indicator of the competitors’ identity due to variations in the stem-peptides^87^, teichoic acids^88^, and PG crosslinking^89^. For instance, PG of *B. subtilis* contained 19% deacetylated N-acetylglucosamine and 33% N-acetylmuramic acid residues, as compared to *B. thuringiensis* PG, where N-acetylglucosamine was deacetylated by 88% and N-acetylmuramic acid by 26%.^83^, besides these variations additional variations were also reported^76^.

Therefore, it seems to provide a cue that fits the competition sensing hypothesis as well as sensitivity sensing. Nevertheless, as PG from sensitive competitors was superior to PG from resistant competitors, the requirement for a specific cue independent of cell damage seems to dominate the sensory machinery for antibiotic production.

Our observation that ComA acts downstream to PG sensing is especially intriguing. ComA is the transcription factor regulating genetic competence, a process in which *B. subtilis* takes up foreign DNA^90^. It is not required for basal expression from bacillaene biosynthesis promoter (Fig. 8a, c). However, it was required to regulate antibiotic production genes’ expression in response to sensitive competitors. These results indicate that genetic competence is co-regulated with a peak of antibiotic production, in the presence of similar but not identical competitors. Furthermore, a recent observation revealed that kin discrimination promotes horizontal gene transfer between unrelated strains in *Bacillus subtilis*^91^ a response mediated by an unknown cue, which could be PG. The link between antibiotic production and competence and their co-regulation may be universal, as competent *Streptococcus pneumoniae* cells produce CibAB, a two-peptide bacteriocin that acts on the membrane of non-competent cells and CibC to confer protection from the bacteriocin activity. Competent cells are then exposed to DNA released from the target cells^92^.

Strongly supporting the hypothesis that PG is linking antibiotic production and competence, ComG and ComA, the master regulators of cell competence are induced by sensitive competitors. Collectively these results suggest a co-evolution between the regulation of antibiotic production and horizontal gene transfer: competitors are sensed by PG receptors, lyzed by the induced antibiotics, and the DNA of these competitors is used to increase genetic diversity^91^. Distinguishing members of related species and unrelated species is of high importance, as members of the same clade are often resistant to their own self-produced antibiotics, and therefore the production of antibiotics under these conditions is more hazardous than beneficial for the producers (Fig. 8f).

Overall, our findings indicate how multispecies communities of similar genotypes are favored during competition, due to compatibility in antibiotic production and sensitivity. Coupling antibiotic production and the identification of sensitive competitors by specific cue and cellular damage (both represented by PG sensing) provides a simple principle that shapes bacterial communities. This principle may be further used for engineering microbiomes for ecological, medical and agricultural applications.

## Methods

### Strains and media

All strains used in this study are listed in Supplementary Table 3.

Selective media for cloning purposes were prepared with LB broth or LB-agar using antibiotics at the following final concentrations: 10 μg/ml chloramphenicol (Amersco), 10 µg/ml, 10 μg/ml spectinomycin (Tivan biotech), 10 µg/ml tetracycline (Amresco), 1 µg/ml erythromycin (Amresco) + 25 µg/ml lincomycin (Sigma). Starter cultures of all strains was prepared using LB (Luria- Bertani) broth (Difco). B4 (0.4% yeast extract, (Difco), 0.5% D-glucose and 0.25% calcium acetate (Sigma Aldrich)) was prepared as described previously^55^.

### Interaction assay

A single colony of each strain was isolated on a solid LB plate, inoculated into 2 ml of LB broth and grown to a mid-logarithmic stage at 37 °C with shaking. Mid-logarithmic cultures of WT *B. subtilis* or its mutants and of each competitor were spotted on B4 plates either alone or at 0.4 cm apart. The plates were then incubated at 30 °C for 48 h and biofilms colonies were photographed using stereomicroscope (Zeiss), using Objective Plain 1.0 × FWD 60 mm lens at 44× magnification. Captured images were processed using Zen software (Zeiss). Images shown for dual reporter WT *B. subtilis* strain harboring of P_*srfAA*_*-yfp* (surfactin), P_*pksC*_*-mKate* (bacillaene) in Fig. 5c were merged using ImageJ.

For CFU analysis, biofilms were harvested at 48 h by scrapping the entire colony of the *Bacilli* and suspending the colony in PBS. This was followed by sonication using a BRANSON digital sonicator, at an amplitude of 10% and a pulse of 5 s to separate the cells without compromising their viability. This protocol was calibrated to separate the aggregates into single cells without compromising their viability. 100 µl of sonicated cells’ suspension was transferred to a Griener 90 well plate (Sigma) and a serial dilution ranging from 10^−1^ to 10^−7^ was performed in PBS (Phosphate-buffered saline). 20 µl of sample was transferred from the dilution series on an LB plate, incubated at 30 °C overnight and then counted. For competition on MSgg medium WT *B. subtilis or* its mutants and each of the competitors were spotted 0.7 cm apart, while the same conditions and setup was used for the growth and CFU count.

### Conditioned medium (CM) preparation

Conditioned medium (CM) refers to filtered supernatant of the culture media: Cells were grown to a mid-logarithmic phase of growth (OD = 0.6–0.8). Cells were diluted 1:100 in 300 ml of B4 medium and grown at 30 °C, in dark for 24 h in a shaker incubator (Brunswick™ Innova® 42). Cells were removed by centrifugation at 8000 × *g* and the growth media was filtered by 0.22 μm filter (Corning). CM was separated into fractions; small fractions (<3 KDa) and large fractions (>3 KDa) using centrifugal filters of size 3 kDa (Amicon Ultra 15; Centrifugal Filer Units, Merck Millipore, Ireland). CM and its fractions were stored at 4 °C for further use.

### Growth measurements

Cells were grown from a single colony isolated over LB plates to a mid-logarithmic phase of growth. Cells were grown in 300 μl of B4 medium in a 96-well microplate (Thermo Scientific), with agitation, at 30 °C for 24 h, in a microplate reader (Synergy 2; BioTek, Winooski, VT, USA), and the optical density at 600 nm was measured every 30 min. Cells were either grown in the presence or absence of CM or its fractions as indicated in each corresponding figures legends.

### Model to estimate interactions between NRPs/PKS antibiotics

Interactions between antibiotics were quantified using the Bliss independence model. The effect of each single antibiotic *i* on a given species $({E}_{i})$ was calculated as:

1 $${E}_{i}={{\log }}_{2}\frac{{N}_{{wt}}}{{N}_{\triangle i}}$$

where ${N}_{{wt}}$ is the CFU of the species when competing with the WT *B. subtilis* strain, and ${N}_{\triangle i}\,$is its CFU when competing with a mutant unable to produce antibiotic *i*. Similarly, the effect of a combination of two antibiotics, *i* and *j*, is calculated as:

2 $${E}_{{ij}}={{\log }}_{2}\frac{{N}_{{wt}}}{{N}_{\triangle {ij}}},$$

where ${N}_{\triangle {ij}}\,$is the CFU of a species competing with a WT *B. subtilis* mutant unable to produce either antibiotic *i* or *j*.

Given the effects of two antibiotics, *i* and *j*, the expected effect of their combination is given by:

3 $$\,{E}_{{ij},{\exp }}=\,{E}_{i}+\,{E}_{j}.$$

The Interaction Score between a pair of antibiotics is then given by:

4 $${{IS}}_{{ij}}=\,{{E}_{{ij}}-E}_{{ij},{\exp }}.$$

An Interaction Score of 0 indicates antibiotics that act independently, whereas a scores smaller (larger) than 0 indicates synergism (antagonism) between the antibiotics.

For the combination of all four antibiotics, the observed effect is calculated as:

5 $$\,{E}_{{ij}}={{\log }}_{2}\frac{{N}_{{wt}}}{{N}_{\triangle 4}},$$

and the expected one is given by:

6 $$\,{E}_{4,{\exp }}=\mathop{\sum }\limits_{i=1}^{4}{E}_{i}.$$

The Interaction score of each of the combination of antibiotics acting on each competitor was calculated based on three independent experiments. An Interaction Score was calculated for each experiment, and the final Interaction Score is given by the average of these three values. To test whether each Interaction Score was significantly different from 0, we used a two-tailed one-sample *t*-test. Subsequently, Fisher’s method^93^ was used to combine all the *p* values calculated for the Interaction Scores of a particular combination of antibiotics and each of the competitor species. The combined *p* value assesses the overall significance of deviation of Interaction Scores from 0 each combination of antibiotics. Similarly, the *p* values calculated for a given competitor across all antibiotics combinations were combined to assess the overall significance of the Interaction Scores for each competitor.

### Flow cytometry analysis

Starter cultures of the WT *B. subtilis* (harboring no fluorescent reporter), indicated fluorescent reporters were spotted on B4 plates. For competition, assay fluorescent reporters were spotted 0.4 cm apart from their competitors. The plates were then incubated at 30 °C and biofilms colonies were harvested at 24 h post-inoculation by scrapping the entire colony of the reporters and suspending the colony in PBS. This was followed by sonication using a BRANSON digital sonicator, at an amplitude of 10% and a pulse of 5 s to separate cells without compromising their viability. Harvesting cells at 24 h allowed us to harvest cells prior to direct contact with their competitors, thus avoiding any potential mixing of reporters and their competitors. Directly after sonication, samples were diluted in PBS and measured using an LSR‐II cytometer (Becton Dickinson, San Jose, CA, USA). The GFP and YFP fluorescence was measured using laser excitation of 488 nm, coupled with 505 LP and 525/50 sequential filters, while *mKate* fluorescence was measured using laser excitation of 561 nm, coupled with 600 LP and 610/20 sequential filters. To distinguish background fluorescence from the reporters’ specific fluorescence, the WT *B. subtilis* was used as a negative control, and its background fluorescence was gated to separate true fluorescent population (population outside the background gate) from the reporters (Supplementary Fig. 27). A total of 100,000 cells were counted for each sample and flow cytometry analyses was performed using FACS Diva (BD biosciences) and FCS express 7 Research Edition.

### Luminescence analysis

Strains carrying the indicated luminescence reporters were grown in either B4 medium (NT) or B4 medium supplemented with the relevant indicated fractions of the indicated strains. A 96-well plate with white opaque walls and clear tissue culture treated flat bottoms (Corning) was used for the measurements. Measurements were performed every 30 min at 30 °C, using a microplate reader (Synergy 2; BioTek, Winooski, VT, USA). Luciferase activity was calculated as RLU/OD. Growth was monitored and was comparable between the different treatments to avoid artefacts related to the normalization of luminescence intensity to the population size.

### Semipolar metabolite extraction and sample preparation

The dried extract was prepared as described in^94^ with minor alterations. Briefly, WT *B. subtilis* cells were grown to a mid-logarithmic phase of growth (OD = 0.6–0.8). Cells were diluted 1:100 in 300 ml of in B4 medium (control), and B4 medium supplemented with 100 ng/µl of the PG from the indicated *Bacilli*. Cells were grown at 30 °C in dark for 24 h in a shaker incubator (Brunswick™ Innova® 42). 100 ml of the cell cultures were collected and conditioned medium was obtained as described previously. The pH of conditioned medium was then adjusted to 2.0 by addition of 6 mol/L HCl, and was kept overnight at 4 °C. The precipitated from HCl treated conditioned medium were obtained by centrifugation at 10,000 × *g* for 20 min at 4 °C. The precipitates were dissolved in 0.5 mL methanol. The tubes were vortexed for half a minute, then sonicated for 30 min in an ice-cold sonication bath (taken for a brief vortex every 10 min), vortexed again, and then centrifuged at 10,000 × *g* at 4 ˚C for 10 min. 200 µL supernatant was transferred into the HPLC vials for analysis.

### LC-MS for semipolar metabolites analysis

Metabolic analysis of semipolar phase was performed using Waters ACQUITY UPLC system coupled to a Vion IMS QTof mass spectrometer (Waters Corp., MA, USA). The chromatographic separation was performed on an ACQUITY UPLC BEH C18 column (2.1 × 100 mm, i.d., 1.7 μm) (Waters Corp., MA, USA). The mobile phase A consisted of 95% water (UPLC grade) and 5% acetonitrile, with 0.1% formic acid; mobile phase B consisted of 100% acetonitrile with 0.1% formic acid. The column was maintained at 45˚C, and the flow rate of mobile phase was 0.4 mL*min^−1^. Mobile phase A was initially run at 100%, and it was gradually reduced to 72% at 22 min, following a decrease to 0% at 36 min. Then, mobile phase B was run at 100% until 38 min; then, mobile phase A was set to 100% at 38.5 min. Finally, column was equilibrated at 100% mobile phase A until 40 min. MS parameters were as follows: the source and de-solvation temperatures were maintained at 120 °C and 350 °C, respectively. The capillary voltage was set to 1 kV; cone voltage was set for 40 V. Nitrogen was used as de-solvation gas and cone gas at the flow rate of 700 L*h^−1^ and 50 L*h^−1^, respectively. The mass spectrometer was operated in full scan HDMS^E^ positive ionization, over a mass range of 50–2000 Da. For the high energy scan function, a collision energy ramp of 20–70 eV was applied, for the low energy scan function 5 eV was applied. Leucine-enkephalin was used as lock-mass reference standard.

### Semipolar compounds identification and data analysis

LC-MS data were analyzed and processed with UNIFI (Version 1.9.4, Waters Corp., MA, USA). The putative identification of the Surfactin was performed by comparison of standard’s and sample’s accurate masses, fragmentation pattern (Supplementary Fig. 15), retention time, and ion mobility (CCS) values. The putative identification of the Bacillaene was performed by studying sample’s peak accurate masses, theoretical fragmentation pattern, retention time, and ion mobility (CCS) values (Supplementary Table 2).

### Statistical analysis

Phylogenetic distances (ANI) between WT *B. subtilis* and competitors were calculated using Integrated Microbial Genomes (IMG) database. Whole genome sequences were used to calculate ANI between *Bacilli*. For *Bacilli* where full genome sequences were not available, strain from the same species with full genome sequences were used for the analysis. The analysis of growth curves was performed as in^95,96^, to allow identical comparison for different growth patterns. All studies were performed in triplicates at least three separate and independent times unless mentioned otherwise. Data were expressed as ±standard deviations of the means unless mentioned otherwise. All graphs were generated and statistical analyses was performed with GraphPad Prism 9.0 (GraphPad Software, Inc., San Diego, CA). The analysis varied for different datasets and details on the statistical methods are reported in the figures legend. Additional methods are available in the supporting information. Additional statistical analysis is attached in Supplementary data 1.

### Reporting summary

Further information on research design is available in the Nature Research Reporting Summary linked to this article.

## Supplementary information

Supplementary Information

Description of Additional Supplementary Files

Supplementary Data 1

Reporting Summary

## Data Availability

There are no applicable accession codes, unique identifiers, or datasets that could be publicly available. All data are available in the article and supplementary information. Source data of all data presented in graphs within the figures are provided with this paper. Source data are provided with this paper.

## Source data

Source Data
