# Supplementary material for: Resolving the conflict between antibiotic production and rapid growth by recognition of peptidoglycan of susceptible competitors
Source: Nat Commun. 2022 Jan 20;13:431. doi: 10.1038/s41467-021-27904-2 (PMC8776889; doi:10.1038/s41467-021-27904-2)
Supplement: Supplementary file 1 — Supplementary Information [file 41467_2021_27904_MOESM1_ESM.pdf]

Resolving the conflict between antibiotic production and rapid growth by recognition of peptidoglycan of susceptible competitors

Harsh Maan[1], Maxim Itkin[2], Sergey Malirsky[2], Jonathan Friedman[3] and Ilana Kolodkin-Gal[1*]

[1]Department of Molecular Genetics, Weizmann Institute of Science, 234 Herzl Street, Rehovot, Israel

[2]Life Science Core Facilities, Weizmann Institute of Science, 234 Herzl Street, Rehovot, Israel

[3]Department of Plant Pathology and Microbiology, Faculty of Agriculture, Food and Environment, The Hebrew University of Jerusalem, Rehovot, Israel.

[*] Correspondence: ilana.kolodkin@mail.huji.ac.il

Supporting Information

Supplementary Figures (1-27)

Supplementary Tables (1- 5)

Supplementary Methods

Supplementary References

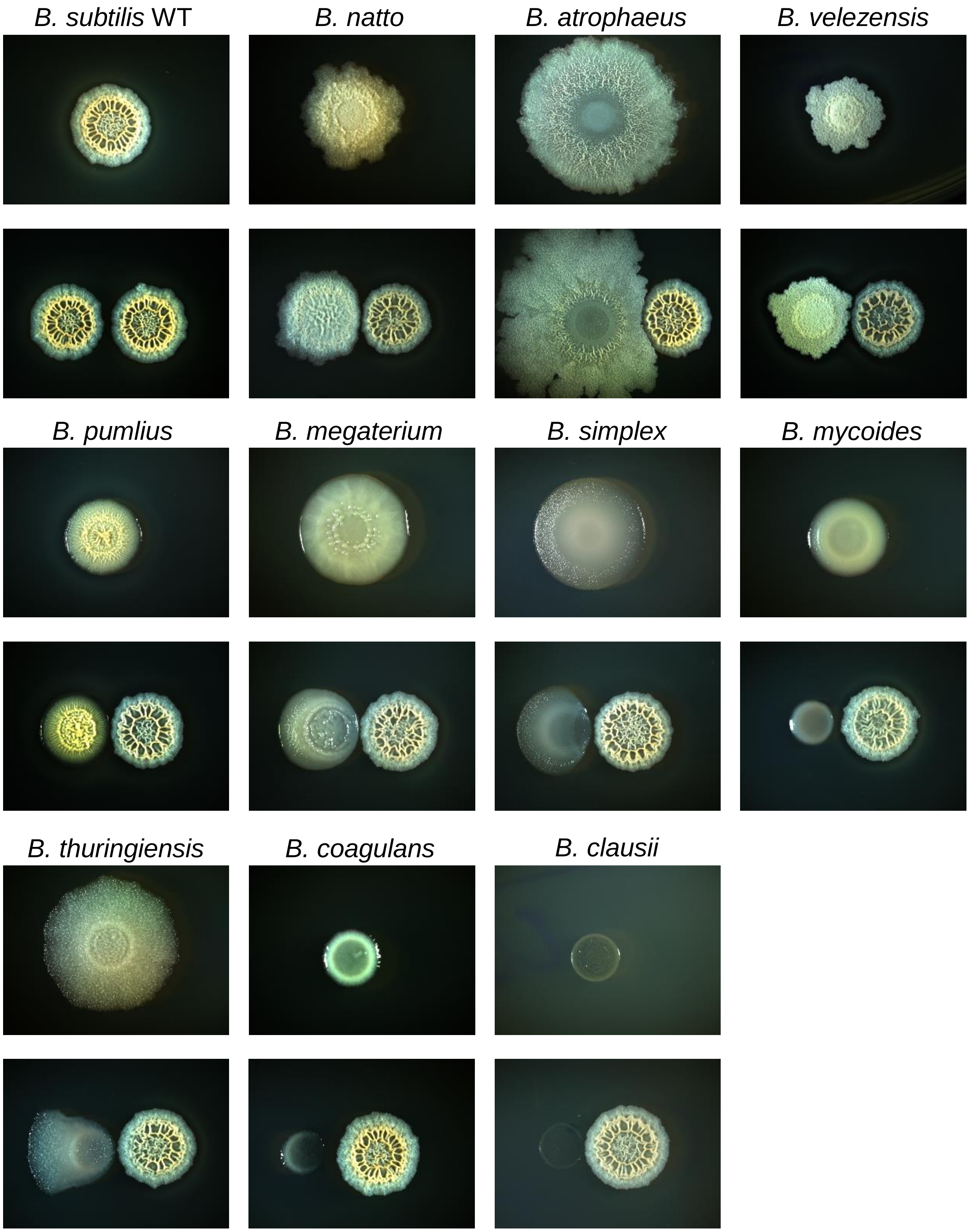

Supplementary Figure 1. Growth in isolation and during competition of indicated *Bacillus* species. For each species, shown is the biofilm growth in isolation versus the biofilm growth during interspecies competition against WT *B. subtilis* colony (below). Images are from representative experiment performed in triplicates out of three independent experiments. Colony biofilms were inoculated at 0.4 cm apart and grown on B4 medium at 30° C. Biofilms colonies were imaged at 48 hours post inoculation. Scale bar = 1mm.

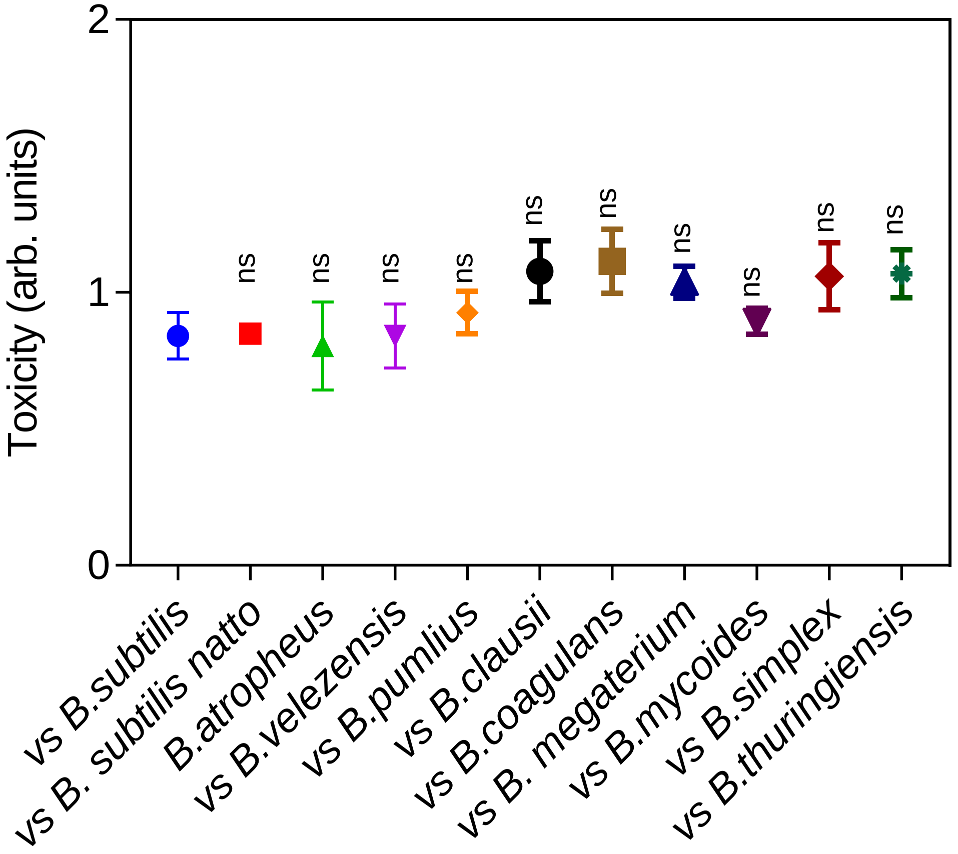

Supplementary Figure 2. Toxicity of *Bacilli* against WT *B. subtilis* was evaluated. Arbitrary units (arb. units) for toxicity were determined as the ratio of WT *B. subtilis* growth alone/growth in competition against *Bacilli*: Biofilm cells were harvested 48 hours post inoculation and colony forming units (CFU) were calculated alone, and during co-inoculation. Graph represent mean ± SD from three independent experiments (n = 9). Statistical analysis was performed using one-way ANOVA followed by Tukey's multiple comparison post hoc testing. $P < 0.05$ was considered statistically significant. No significant differences were observed between the toxicity exhibited on WT *B.* subtilis when competed against the indicated *Bacilli*. Source data are provided as a Source Data file.

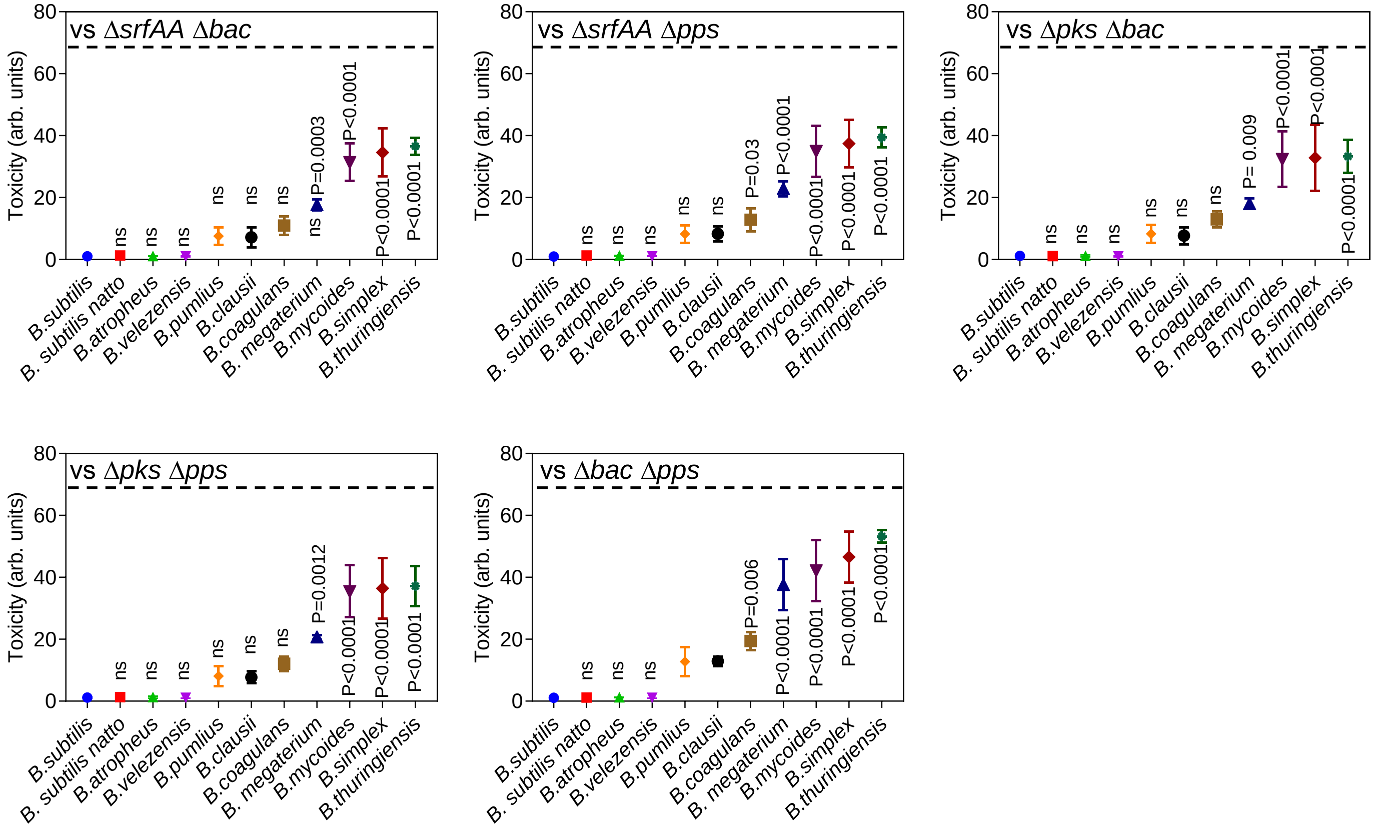

Supplementary Figure 3. Toxicity of indicated NRPs/PKS mutants towards *Bacilli* was evaluated. Arbitrary units (arb. units) for toxicity were determined as the ratio of *Bacilli* growth alone/growth in competition against indicated mutants: Biofilm cells were harvested 48 hours post inoculation and colony forming units (CFU) were calculated alone, and during co-inoculation. Graphs represent mean ± SD from three independent experiments (n = 9). Statistical analysis was performed using one-way ANOVA followed by Tukey's multiple comparison post hoc testing. $P < 0.05$ was considered statistically significant. Significant differences between the toxicity towards WT *B. subtilis* and toxicity towards indicated *Bacilli*, when competed against the indicated NRPs/PKS mutants are shown by their respective p values. Dashed Line: The maximal toxicity exhibited by WT *B. subtilis.* Source data are provided as a Source Data file.

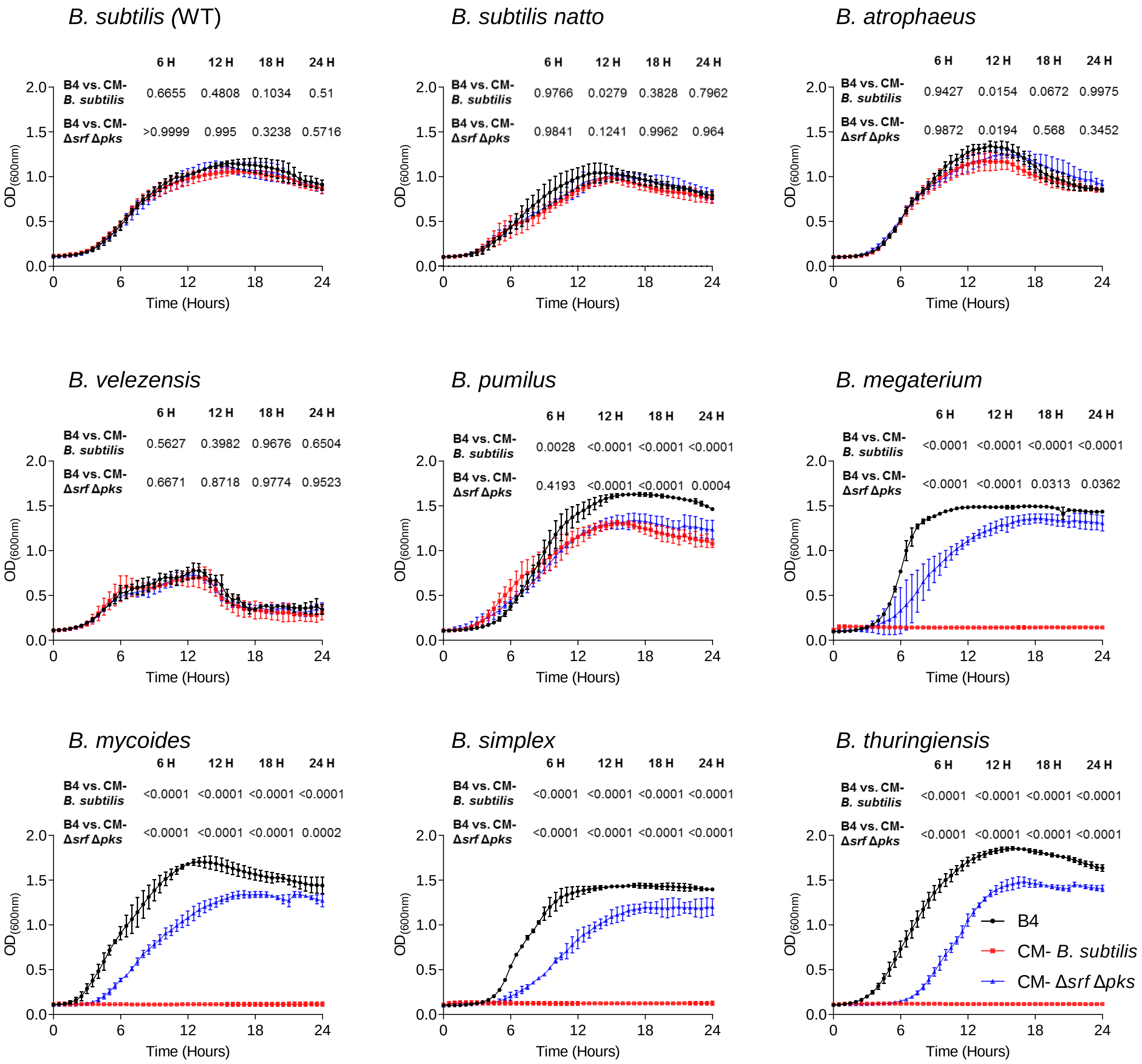

Supplementary Figure 4. Planktonic growth of the indicated species was monitored either in B4 medium (control) or B4 medium supplemented with CM (15% v/v) of WT *B. subtilis* or its NRP/PKS double mutant ΔsrfAA, Δpks (surfactin, bacillaene). Graphs represent mean ± SD from three independent experiments (n = 9). Statistical analysis was performed using two-way ANOVA followed by Dunnett's multiple comparison test. P < 0.05 was considered statistically significant. P values at different time points are shown above each graph. Source data are provided as a Source Data file.

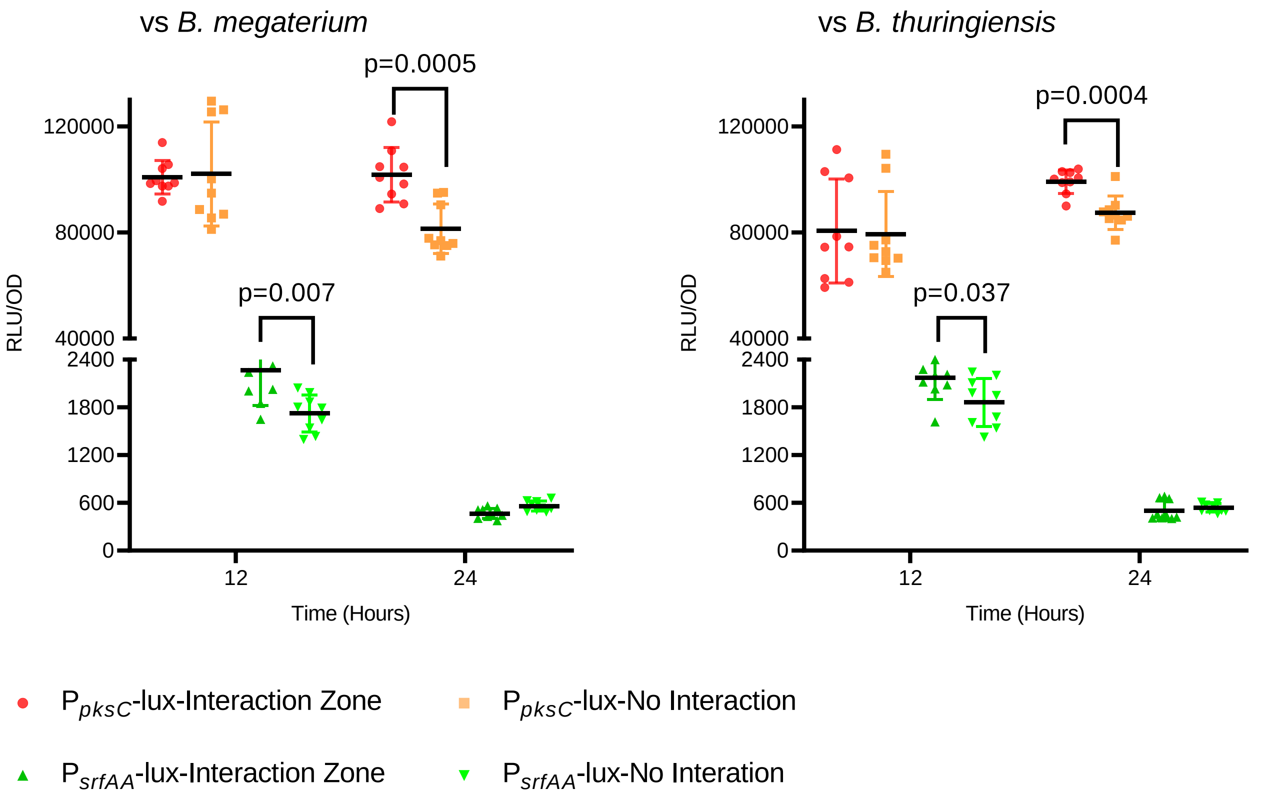

Supplementary Figure 5. Analysis of polarization of surfactin and bacillaene during interspecies competition. WT *B. subtilis* strain harboring P_{srfAA}-*lux* (surfactin) and P_{pksC}-*lux* (bacillaene) reporters were competed against indicated *Bacilli*. Colonies were were inoculated at 0.4 cm and grown on on B4 medium at 30° C, and cells were harvested at 12 hours and 24 hours post inoculation. Interaction zone represent the side of luciferase reporters colonies facing *Bacilli,* while no- interaction is the side of the luciferase reporters colonies not facing the *Bacilli.* Statistical analysis was performed between interaction zone and no interaction, using two tail-unpaired *t*-test with Welch correction. $p < 0.05$ was considered statistically significant. Graphs represent mean (black line) ± SD from three independent experiments (n = 9). Source data are provided as a Source Data file.

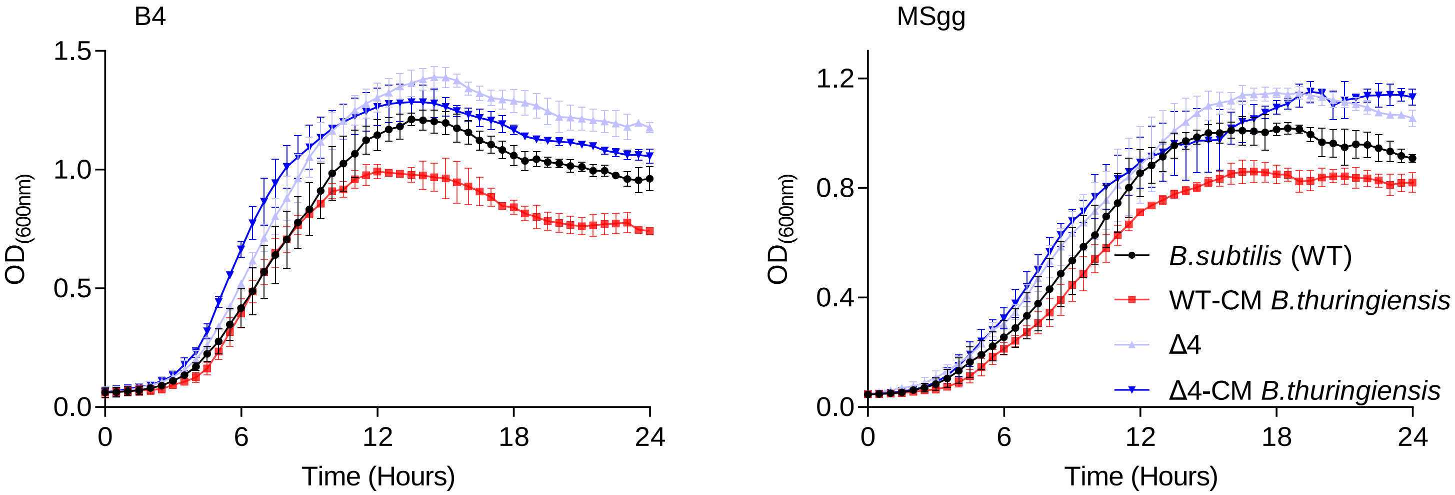

	6 Hours	12 Hours	18 Hours	24 Hours
B.subtilis - B4 vs. *B.subtilis* -CM *B.thuringiensis*	0.9551	0.0021	<0.0001	0.0002
B.subtilis - B4 vs. Δ4-B4	0.0774	0.0015	<0.0001	0.0003
B.subtilis - B4 vs. Δ4-CM *B.thuringiensis*	<0.0001	0.0316	0.0594	0.2719
B.subtilis -CM *B.thuringiensis* vs. Δ4-B4	0.0191	<0.0001	<0.0001	<0.0001
B. subtilis -CM *B.thuringiensis* vs. Δ4-CM *B.thuringiensis*	<0.0001	<0.0001	<0.0001	<0.0001
Δ4-B4 vs. Δ4-CM *B.thuringiensis*	0.0044	0.7904	0.0237	0.1029

	6 Hours	12 Hours	18 Hours	24 Hours
B.subtilis - MSgg vs. *B.subtilis* -CM *B.thuringiensis*	0.7619	0.0056	0.001	0.1789
B.subtilis - MSgg vs. Δ4-MSgg	0.6983	0.9074	0.0115	0.0043
B.subtilis - MSgg vs. Δ4-CM *B.thuringiensis*	0.3821	0.8098	0.2603	<0.0001
B.subtilis -CM *B.thuringiensis* vs. Δ4-Msgg	0.1664	0.0005	<0.0001	<0.0001
B. subtilis -CM *B.thuringiensis* vs. Δ4-CM *B.thuringiensis*	0.0505	0.0002	<0.0001	<0.0001
Δ4-Msgg vs. Δ4-CM *B.thuringiensis*	0.9568	0.9964	0.5921	0.2892

Supplementary Figure 6. Induction of NRP synthesis has deleterious effects on the producers. Growth of indicated strains (*B. subtilis* and Δ4) when treated with the conditioned medium from *B. thuringiensis* (BT), in B4 (15% v/v) and MSgg (12% v/v). Reduction of growth by the *B. thuringiensis* supernatant was dependent on the presence of NRP biosynthetic clusters. The inducing conditioned medium significantly increased the growth inhibition of the *B.subtilis*, but not of the quadruple mutant. Graphs represent mean ± SD from three independent experiments (n = 9). Statistical analysis was performed using two-way ANOVA followed by Tukey's multiple comparison post hoc testing. $P < 0.05$ was considered statistically significant. P values at different time points are shown in the table. Source data are provided as a Source Data file.

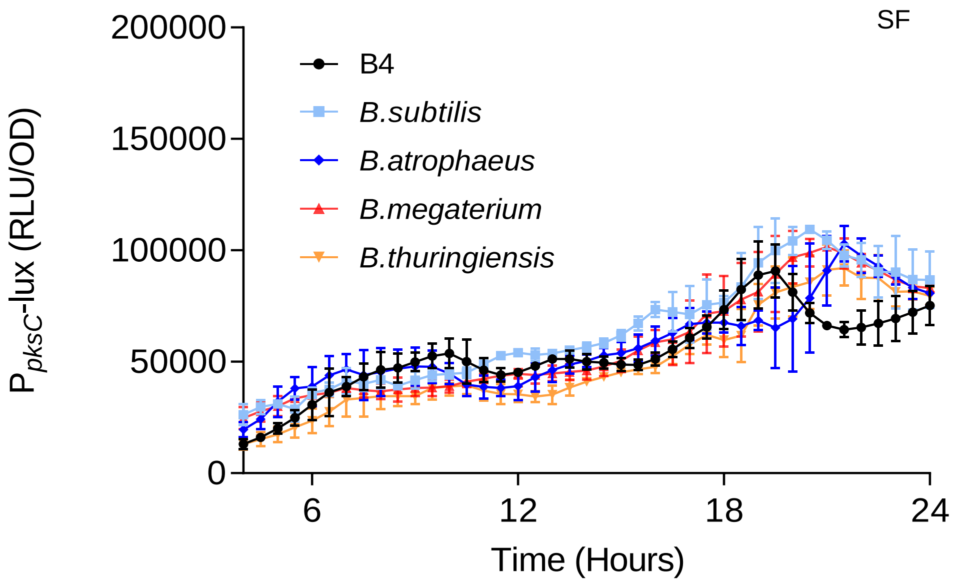

	6 Hours	12 Hours	18 Hours	24 Hours
B4 vs. *B.subtilis* -SF	0.999	0.5309	0.9783	0.2803
B4 vs. *B.atrophaeus* -SF	0.645	0.7905	0.8159	0.8447
B4 vs. *B.megaterium* -SF	>0.9999	0.9999	0.9998	0.6277
B4 vs. *B.thuringiensis* -SF	0.5376	0.4248	0.154	0.9197

Supplementary Figure 7. Analysis of the luciferase activity in a WT *B. subtilis* strain harboring P_{pksC}-*lux* (bacillaene) reporter. Luminescence was monitored in B4 medium (No Treatment), and B4 medium supplemented with 15% v/v of the SF (<3kDa) of the conditioned medium from the indicated species. Graphs represent mean ± SD from three independent experiments (n = 9). Statistical analysis was performed using two-way ANOVA followed by Dunnett's multiple comparison test. $P < 0.05$ was considered statistically significant. P values at different time points are shown in the table. Source data are provided as a Source Data file.

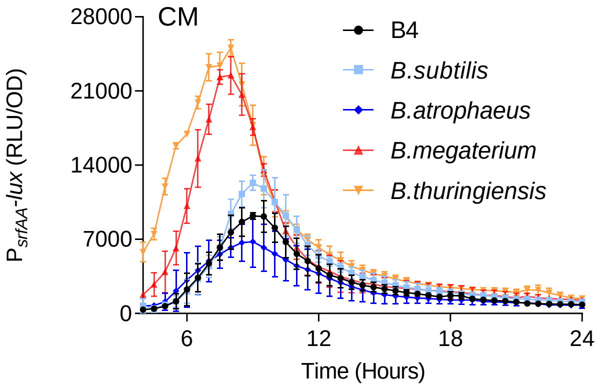

	6 Hours	8 Hours	12 Hours	18 Hours	24 Hours
B4 vs. *B.subtilis* -CM	0.9988	0.1887	0.5361	0.9935	0.9974
B4 vs. *B.atrophaeus* -CM	0.729	0.3377	0.951	0.9756	>0.9999
B4 vs. *B.megaterium* -CM	<0.0001	<0.0001	0.9997	0.9856	0.9866
B4 vs. *B.thuringiensis* -CM	<0.0001	<0.0001	0.1082	0.8127	0.9593

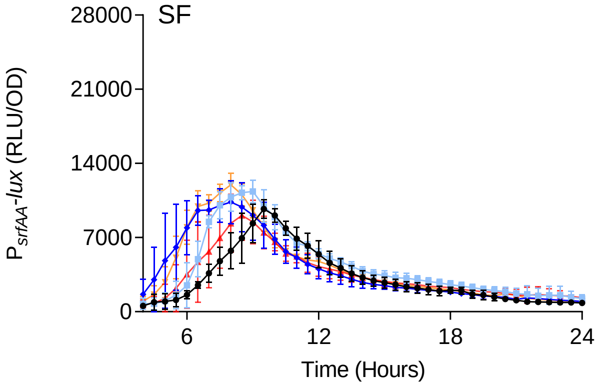

	6 Hours	8 Hours	12 Hours	18 Hours	24 Hours
B4 vs. *B.subtilis* -SF	0.7786	<0.0001	>0.9999	0.9565	0.9911
B4 vs. *B.atrophaeus* -SF	<0.0001	<0.0001	0.4284	0.999	0.9998
B4 vs. *B.megaterium* -SF	0.1602	0.0324	0.5686	0.9969	0.9835
B4 vs. *B.thuringiensis* -SF	<0.0001	<0.0001	0.8957	>0.9999	0.9998

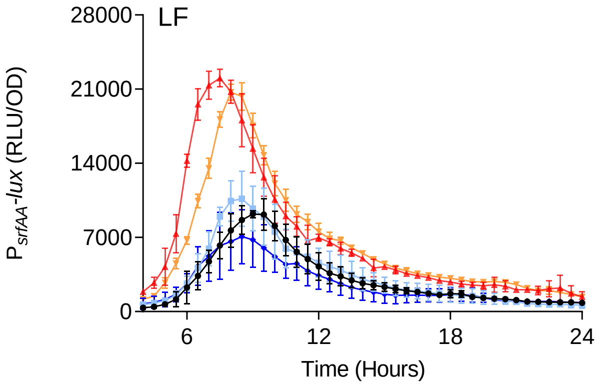

	6 Hours	8 Hours	12 Hours	18 Hours	24 Hours
B4 vs. *B.subtilis* -LF	0.9814	0.0078	0.9886	>0.9999	0.9989
B4 vs. *B.atrophaeus* -LF	0.9783	0.6051	0.7412	0.9999	>0.9999
B4 vs. *B.megaterium* -LF	<0.0001	<0.0001	0.0112	0.5596	0.9564
B4 vs. *B.thuringiensis* -LF	<0.0001	<0.0001	0.0012	0.3017	0.9622

Supplementary Figure 8. Analysis of the luciferase activity in a WT *B. subtilis* strain harboring P_{srfAA}-*lux* (surfactin) reporter. Luminescence was monitored in B4 medium (No Treatment), and B4 medium supplemented with 15% v/v of the conditioned medium (CM), or an equivalent amount of the conditioned medium fractionated to generate LF (>3kDa) and SF (<3kDa) from the indicated species. Graphs represent mean ± SD from three independent experiments (n = 9). Statistical analysis was performed using two-way ANOVA followed by Dunnett's multiple comparison test. *P* < 0.05 was considered statistically significant. P values at different time points are shown in the table. Source data are provided as a Source Data file.

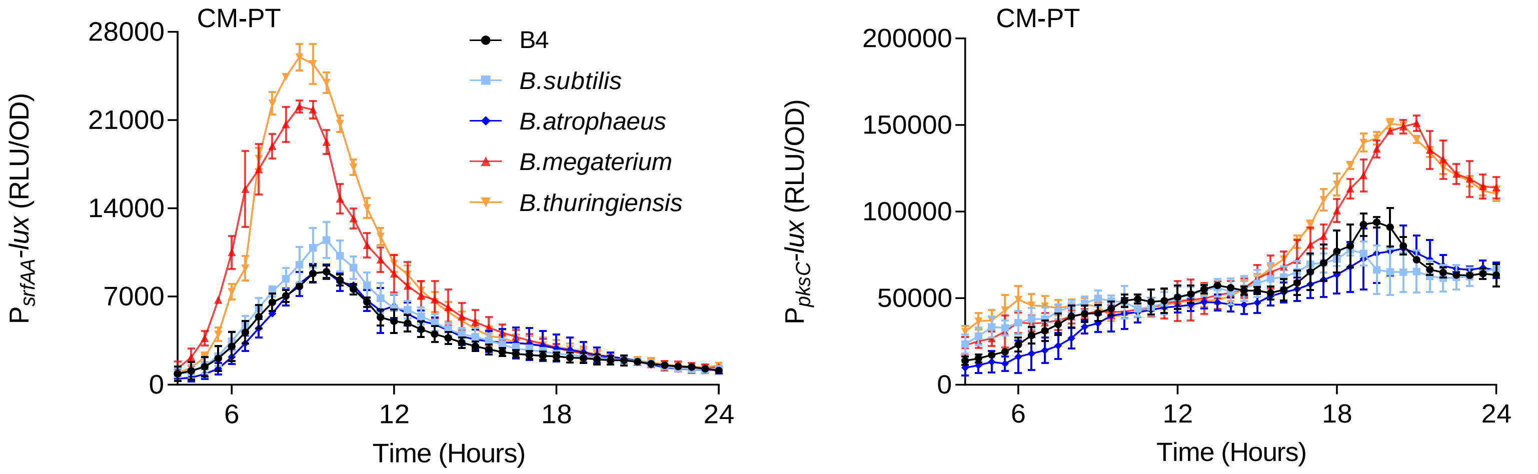

	6 Hours	8 Hours	12 Hours	18 Hours	24 Hours
B4 vs. *B.subtilis* -CM-PT	0.9289	0.1646	0.369	0.9902	0.9993
B4 vs. *B.atrophaeus* -CM-PT	0.5692	0.9997	0.2536	0.7437	0.9999
B4 vs. *B.megaterium* -CM-PT	<0.0001	<0.0001	<0.0001	0.7343	0.9921
B4 vs. *B.thuringiensis* -CM-PT	<0.0001	<0.0001	<0.0001	0.626	0.9709

	6 Hours	12 Hours	18 Hours	24 Hours
B4 vs. *B.subtilis* -CM-PT	0.0997	>0.9999	0.8525	0.8961
B4 vs. *B.atrophaeus* -CM-PT	0.5572	0.7703	0.0695	0.9684
B4 vs. *B.megaterium* -CM-PT	0.0911	0.9889	0.0002	<0.0001
B4 vs. *B.thuringiensis* -CM-PT	<0.0001	0.9686	<0.0001	<0.0001

Supplementary Figure 9. Analysis of the luciferase activity in a WT *B. subtilis* strain harboring P_{srfAA}-*lux* (surfactin) and P_{pksC}-*lux* (bacillaene) reporter. Luminescence was monitored in B4 medium (No Treatment), and B4 medium supplemented with 15% v/v of the protease treated conditioned medium (CM-PT) from the indicated species. Graphs represent mean ± SD from three independent experiments (n = 9). Statistical analysis was performed using two-way ANOVA followed by Dunnett's multiple comparison test. *P* < 0.05 was considered statistically significant. P values at different time points are shown in the table. Source data are provided as a Source Data file.

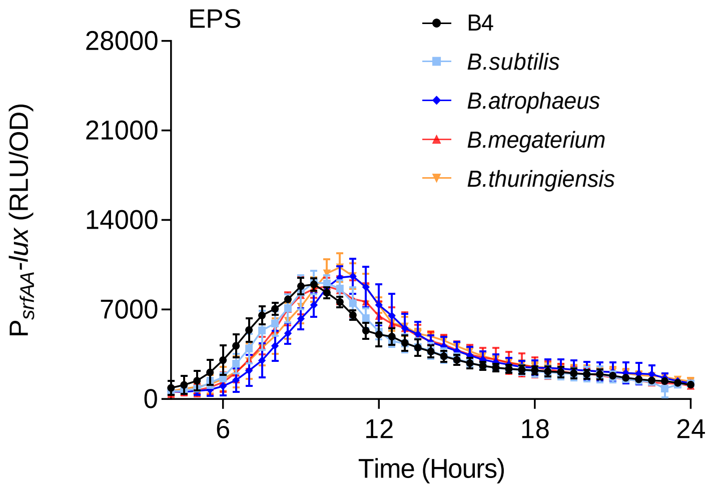

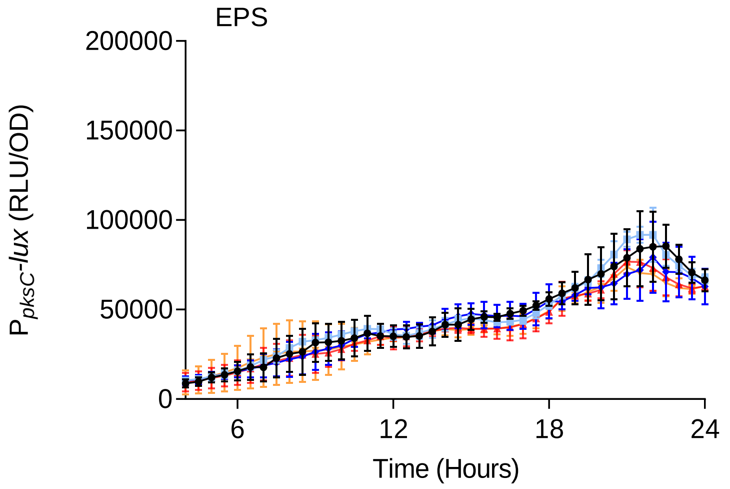

	6 Hours	8 Hours	12 Hours	18 Hours	24 Hours
B4 vs. *B.subtilis* -EPS	0.1938	0.9916	0.9998	0.9998	0.9999
B4 vs. *B.atrophaeus* -EPS	0.0167	0.0046	0.9936	0.9936	0.9998
B4 vs. *B.megaterium* -EPS	0.0528	0.129	0.9916	0.9916	0.9998
B4 vs. *B.thuringiensis* -EPS	0.1251	0.0045	0.9819	0.9819	0.9941

	6 Hours	12 Hours	18 Hours	24 Hours
B4 vs. *B.subtilis* -EPS	0.9999	0.9992	0.9634	0.9998
B4 vs. *B.atrophaeus* -EPS	0.9997	0.9323	0.9988	0.9554
B4 vs. *B.megaterium* -EPS	0.9998	0.9998	0.624	0.955
B4 vs. *B.thuringiensis* -EPS	0.978	0.9998	0.6746	0.9668

Supplementary Figure 10. Analysis of the luciferase activity in a WT *B. subtilis* strain harboring P_{srfAA}-*lux* (surfactin) and P_{pksC}-*lux* (bacillaene) reporter. Luminescence was monitored in B4 medium (No Treatment), and B4 medium supplemented with ESP (15% v/v) from the indicated species. Graphs represent mean ± SD from three independent experiments (n = 9). Statistical analysis was performed using two-way ANOVA followed by Dunnett's multiple comparison test. *P* < 0.05 was considered statistically significant. P values at different time points are shown in the table. Source data are provided as a Source Data file.

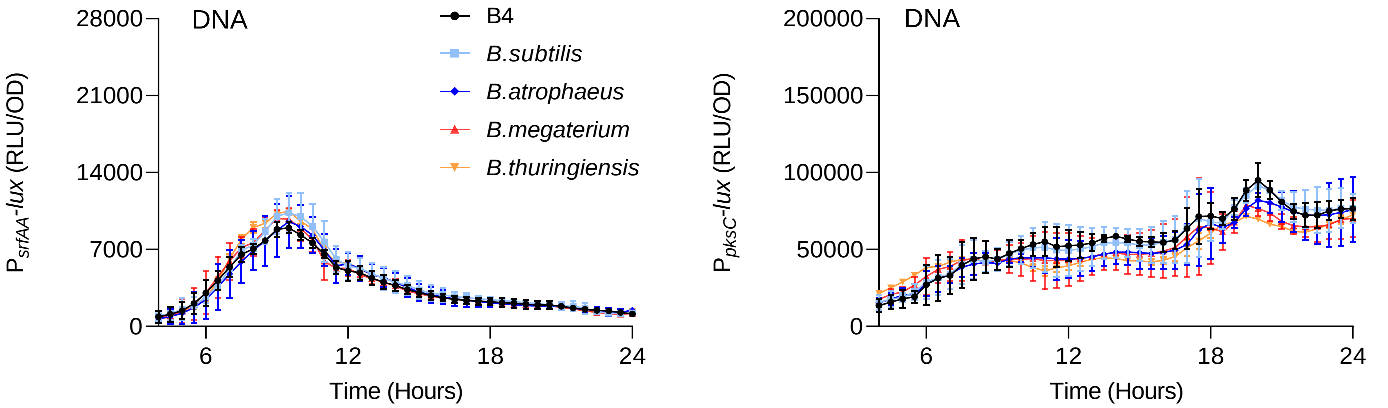

	6 Hours	8 Hours	12 Hours	18 Hours	24 Hours
B4 vs. *B.subtilis* -DNA	0.8892	0.9654	0.9085	0.9955	0.9998
B4 vs. *B.atrophaeus* -DNA	0.806	0.9969	0.7486	0.9997	0.9787
B4 vs. *B.megaterium* -DNA	0.9671	0.8412	0.9857	>0.9999	0.9999
B4 vs. *B.thuringiensis* -DNA	0.9999	0.0167	0.6425	>0.9999	0.997

	6 Hours	12 Hours	18 Hours	24 Hours
B4 vs. *B.subtilis* -DNA	0.9988	0.99	0.9711	>0.9999
B4 vs. *B.atrophaeus* -DNA	0.9998	0.6853	0.9474	0.9999
B4 vs. *B.megaterium* -DNA	0.9195	0.659	0.7766	0.8699
B4 vs. *B.thuringiensis* -DNA	0.4677	0.3398	0.4876	0.9643

Supplementary Figure 11. Analysis of the luciferase activity in a WT *B. subtilis* strain harboring P_{srfAA}-*lux* (surfactin) and P_{pksC}-*lux* (bacillaene) reporter. Luminescence was monitored in B4 medium (No Treatment), and B4 medium supplemented with DNA (≈100 ng/µl) from the indicated species. Graphs represent mean ± SD from three independent experiments (n = 9). Statistical analysis was performed using two-way ANOVA followed by Dunnett's multiple comparison test. *P* < 0.05 was considered statistically significant. P values at different time points are shown in the table. Source data are provided as a Source Data file.

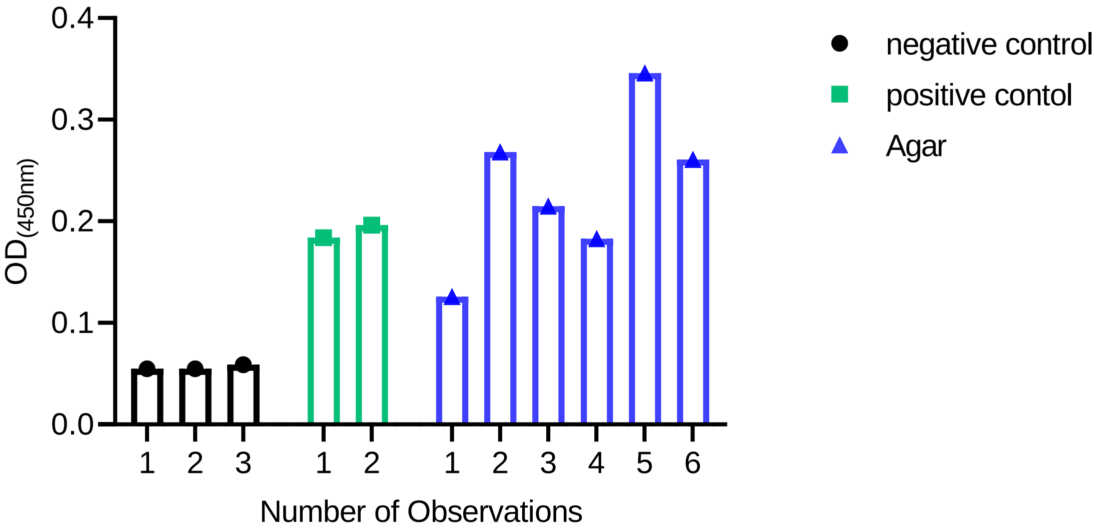

Supplementary Figure 12. Confirming the presence of PG in B4 agar medium using ELISA test. *B. thuringiensis* was grown for 48 hours at 30° C, and 1.5 mm rings of agar surrounding the *B. thuringiensis* biofilm colony were cut and suspended in PBS. Positive controls were prepared by dissolving purified peptidoglycan provided by the manufacturer in standard diluent buffer, and for negative control, standard diluted buffer was used. Successive incubations and treatments were performed as indicated in the manufacturer protocol. Y- axis represents the recorded OD at 450 nm. Source data are provided as a Source Data file.

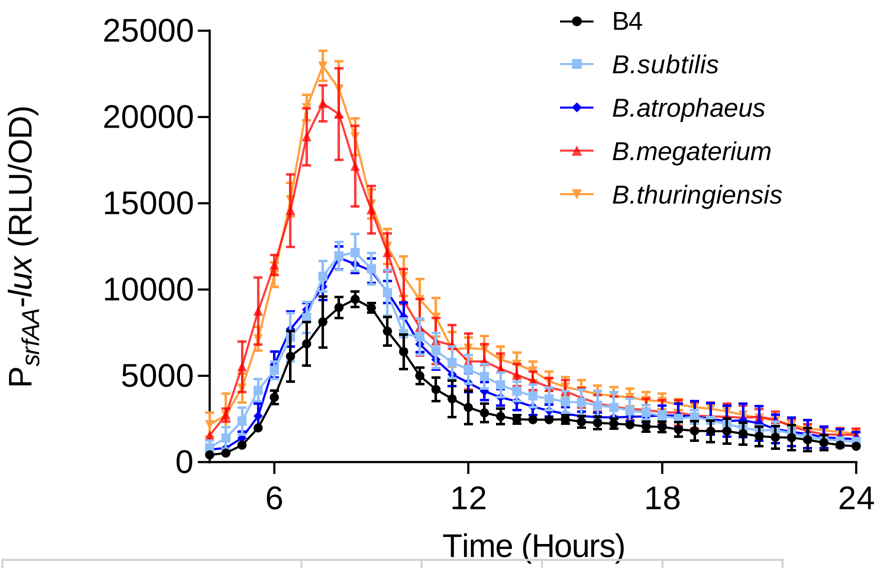

	6 hours	8 hours	12 hours	18 hours
B4 vs. *B. subtilis* -PG	0.1201	0.0003	0.0113	0.8011
B4 vs. *B. atrophaeus* -PG	0.0384	0.0006	0.1645	0.7716
B4 vs. *B.megaterium* -PG	<0.0001	<0.0001	0.0013	0.5981
B4 vs. *B.thuringiensis* -PG	<0.0001	<0.0001	<0.0001	0.145

Supplementary Figure 13. Analysis of the luciferase activity in a WT *B. subtilis* strain harboring P_{srfAA}-*lux* (surfactin) reporter. Luminescence was monitored in B4 medium (No Treatment), and B4 medium supplemented with PG (100 ng/µl) from the indicated species. Graphs represent mean ± SD from three independent experiments (n = 9). Statistical analysis was performed using two-way ANOVA followed by Dunnett's multiple comparison test. *P* < 0.05 was considered statistically significant. P values at different time points are shown in the table. Source data are provided as a Source Data file.

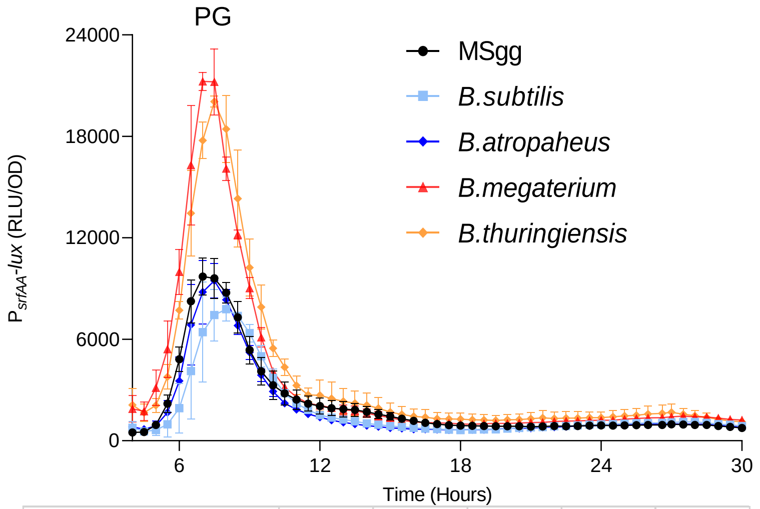

	6 Hours	8 Hours	12 Hours	18 Hours	24 Hours
MSgg vs. *B.subtilis* -PG	<0.0001	0.1961	0.7021	0.9628	0.9998
MSgg vs. *B.atrophaeus* -PG	0.0528	0.8443	0.5264	0.9682	>0.9999
MSgg vs. *B.megaterium* -PG	<0.0001	<0.0001	>0.9999	0.999	0.9383
MSgg vs. *B.thuringiensis* -PG	<0.0001	<0.0001	0.5704	0.8889	0.791

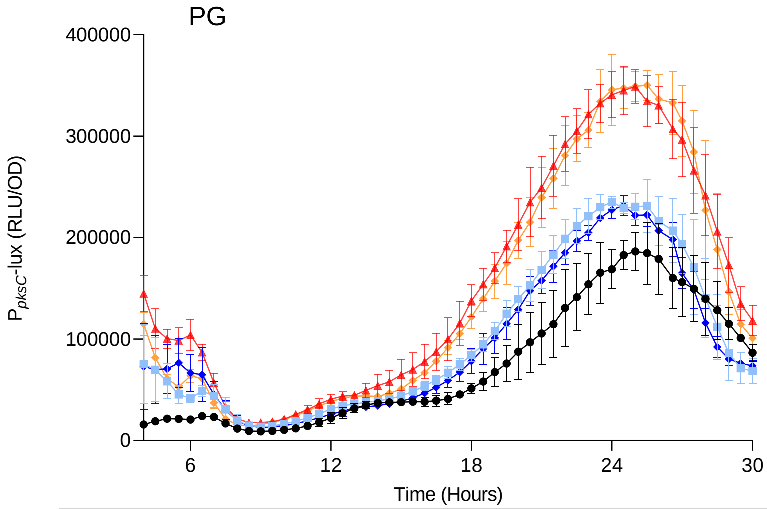

	6 Hours	12 Hours	18 Hours	24 Hours	30 Hours
MSgg vs. *B.subtilis* -PG	0.4144	0.9347	0.0871	<0.0001	0.5415
MSgg vs. *B.atrophaeus* -PG	0.0067	0.9972	0.2071	0.0003	0.7891
MSgg vs. *B.megaterium* -PG	<0.0001	0.5225	<0.0001	<0.0001	0.1025
MSgg vs. *B.thuringiensis* -PG	0.0124	0.6382	<0.0001	<0.0001	0.7242

Supplementary Figure 14. Analysis of the luciferase activity in a WT *B. subtilis* strain harboring P_{srfAA}-*lux* (surfactin) and P_{pksC}-*lux* (bacillaene) reporter. Luminescence was monitored in MSgg medium (No Treatment), and MSgg medium supplemented with PG (100 ng/µl) from the indicated species. Graphs represent mean ± SD from three independent experiments (n = 9). Statistical analysis was performed using two-way ANOVA followed by Dunnett's multiple comparison test. $P < 0.05$ was considered statistically significant. P values at different time points are shown in the table. Source data are provided as a Source Data file.

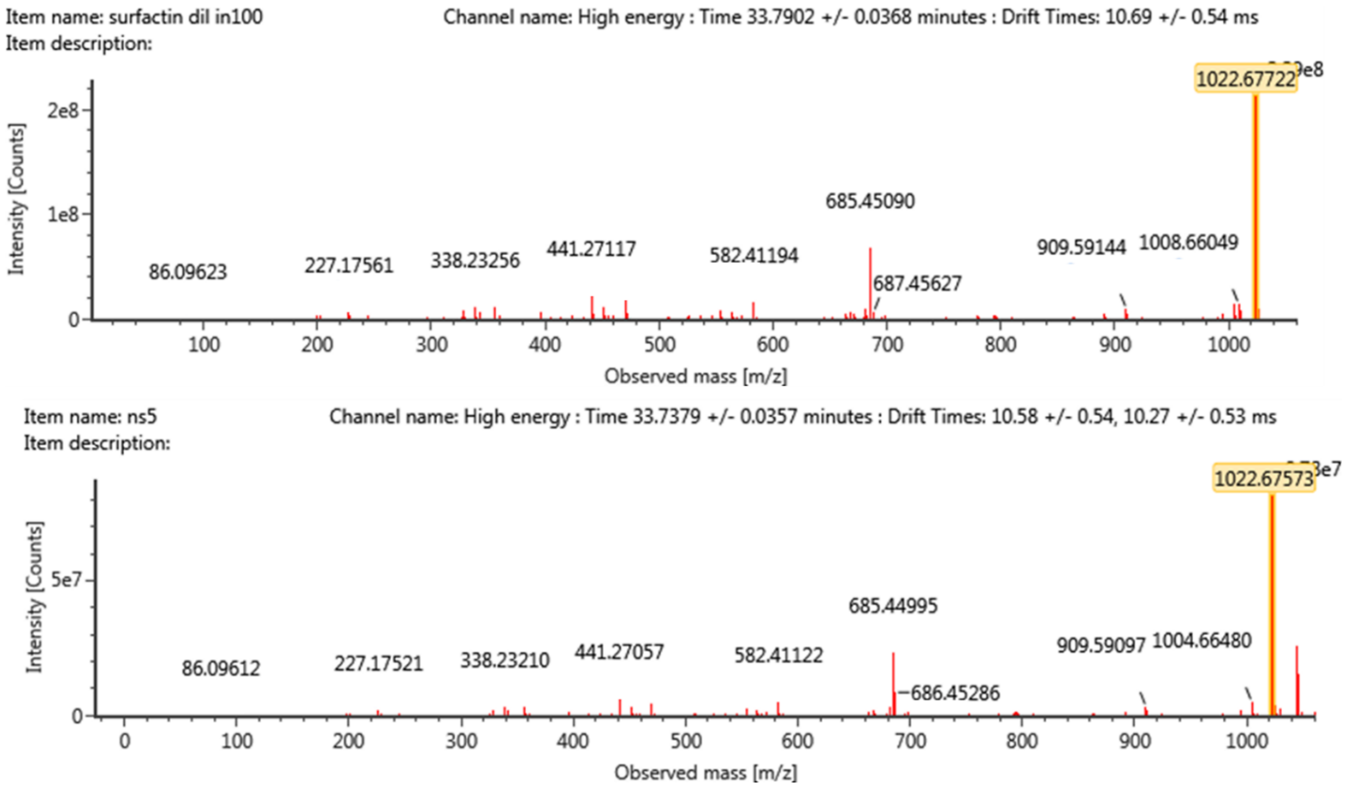

Supplementary Figure 15. Liquid chromatography-mass spectrometry chromatogram comparing between surfactin standard and representative sample's accurate masses, fragmentation pattern, retention time, and ion mobility (CCS) values.

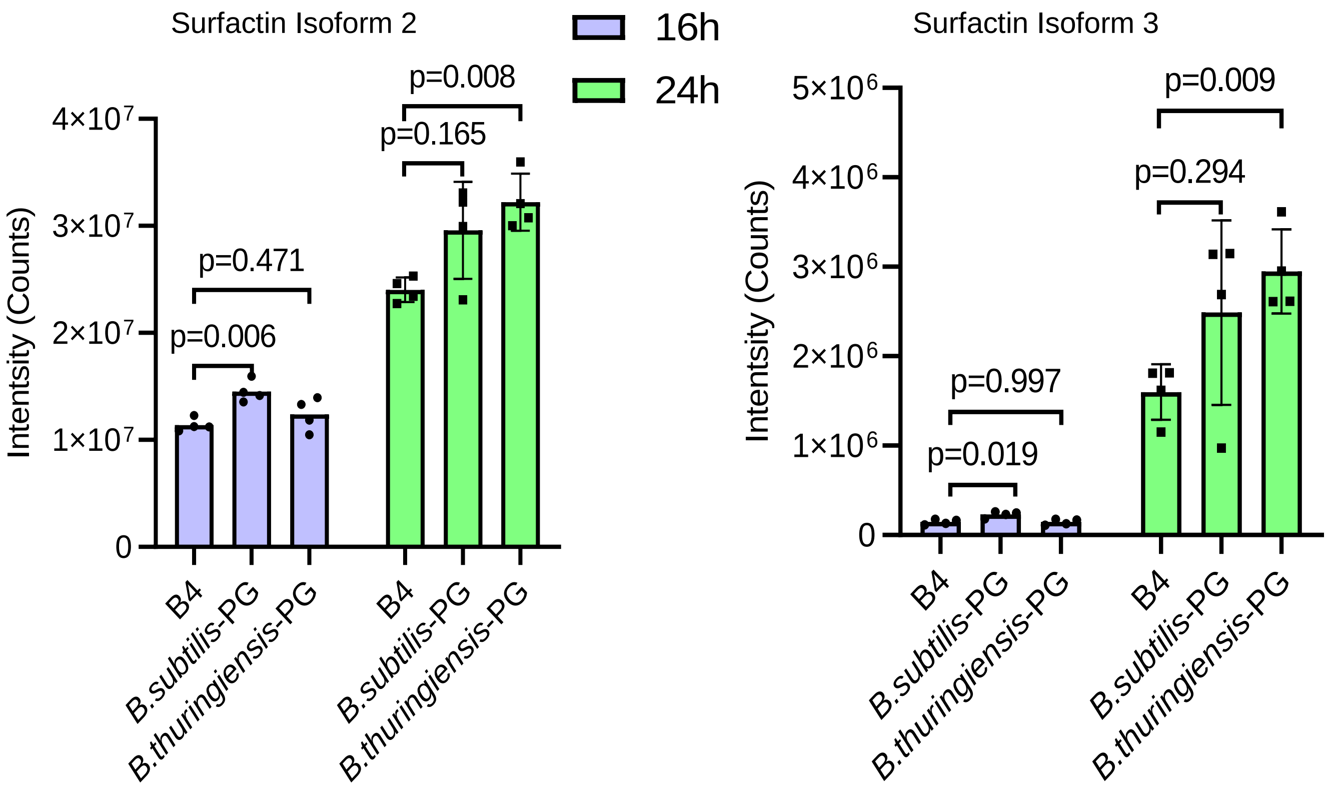

Supplementary Figure 16. Liquid chromatography-mass spectrometry analysis of surfactin isoform 2 and surfactin isoform 3 from the parental strain *B.subtilis* grown in untreated B4 medium (control), and B4 medium supplemented with PG (100 ng/µl) from the indicated species. Supernatant was extracted from the samples at 16h and 24h using HCl treatment. Graphs represent mean ± SEM from four biological repeats (n = 4). Statistical analysis was performed using Brown-Forsthye and Welch's ANOVA with Dunnett's T3 multiple comparisons test. $P < 0.05$ was considered statistically significant. Source data are provided as a Source Data file.

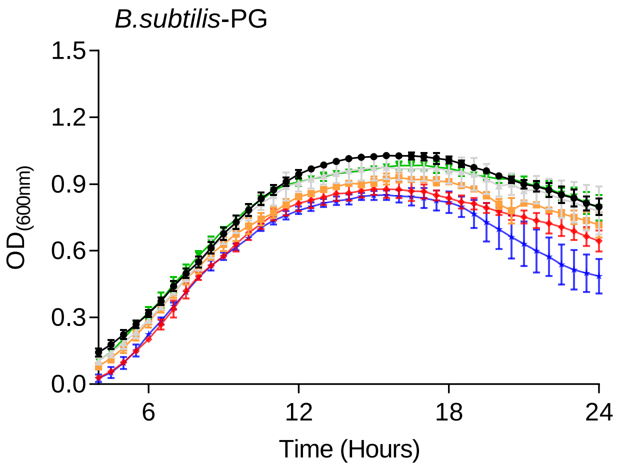

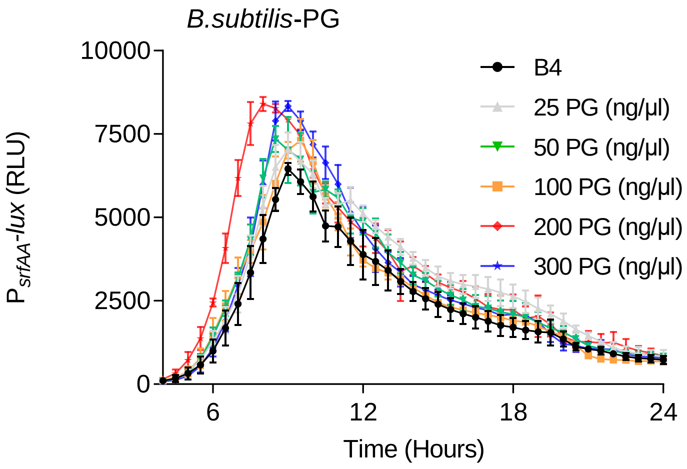

	6 Hours	8 Hours	12 Hours	18 Hours	24 Hours
B4 vs. 25 PG (ng/µl)	0.7831	0.9982	0.5554	0.3372	0.9637
B4 vs. 50 PG (ng/µl)	0.9998	0.8126	0.6645	0.6025	0.9998
B4 vs. 100 PG (ng/µl)	0.5998	0.8818	0.0032	0.0053	0.0229
B4 vs. 200 PG (ng/µl)	0.0005	0.1324	<0.0001	<0.0001	<0.0001
B4 vs. 300 PG (ng/µl)	0.0074	0.0686	<0.0001	<0.0001	<0.0001

	6 Hours	8 Hours	12 Hours	18 Hours	24 Hours
B4 vs. 25 PG (ng/µl)	0.7737	0.0035	0.0002	0.0068	0.9961
B4 vs. 50 PG (ng/µl)	0.4314	<0.0001	0.0032	0.5334	0.9957
B4 vs. 100 PG (ng/µl)	0.305	0.2876	0.9716	0.9414	0.9997
B4 vs. 200 PG (ng/µl)	0.998	<0.0001	0.0898	0.5575	0.9997
B4 vs. 300 PG (ng/µl)	<0.0001	<0.0001	0.0891	0.2553	0.9943

Supplementary Figure 17. Analysis of the growth (OD) and luciferase activity (RLU) in a WT *B. subtilis* strain harboring P_{srfAA}-*lux* (surfactin) reporter. Growth and luminescence were monitored in B4 medium (No Treatment), and B4 medium supplemented with different PG concentrations (25- 300 ng/µl) from WT *B. subtilis*. Graphs represent mean ± SD from three independent experiments (n = 9). Statistical analysis was performed using two-way ANOVA followed by Dunnett's multiple comparison test. $P < 0.05$ was considered statistically significant. P values at different time points are shown in the table. Source data are provided as a Source Data file.

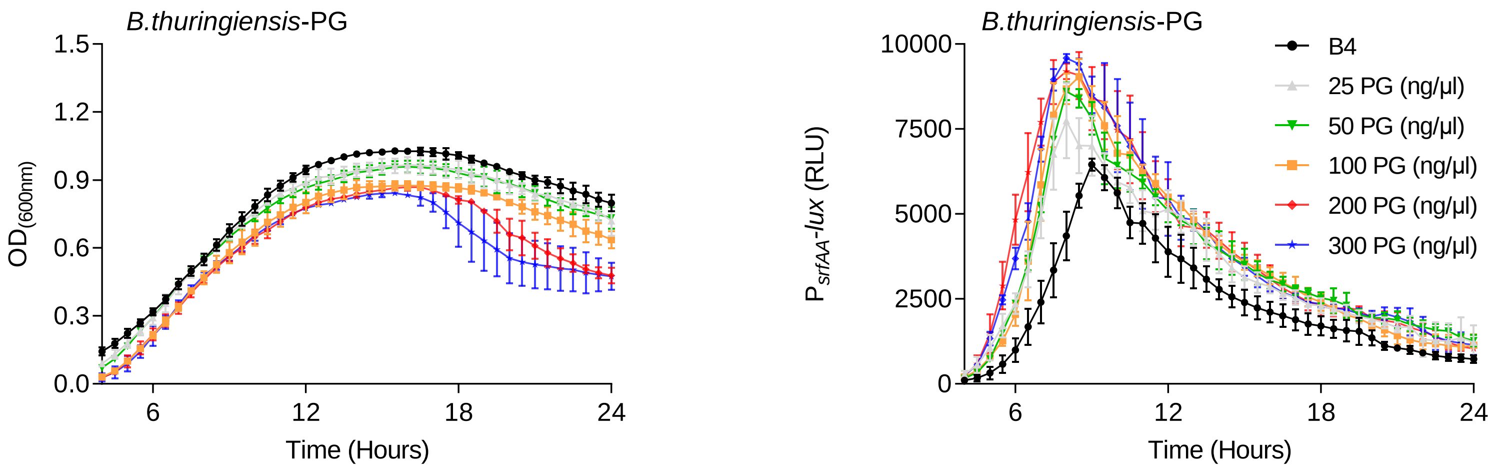

	6 Hours	8 Hours	12 Hours	18 Hours	24 Hours
B4 vs. 25 PG (ng/µl)	0.862	0.9998	0.2306	0.1403	0.0387
B4 vs. 50 PG (ng/µl)	0.8813	>0.9999	0.0373	0.0452	0.1136
B4 vs. 100 PG (ng/µl)	0.0034	0.0275	<0.0001	<0.0001	<0.0001
B4 vs. 200 PG (ng/µl)	0.0047	0.0126	<0.0001	<0.0001	<0.0001
B4 vs. 300 PG (ng/µl)	0.002	0.0699	<0.0001	<0.0001	<0.0001

	6 Hours	8 Hours	12 Hours	18 Hours	24 Hours
B4 vs. 25 PG (ng/µl)	0.0021	<0.0001	0.0058	0.4047	0.5888
B4 vs. 50 PG (ng/µl)	0.0019	<0.0001	0.0086	0.1273	0.4954
B4 vs. 100 PG (ng/µl)	0.0289	<0.0001	0.0002	0.2427	0.7454
B4 vs. 200 PG (ng/µl)	<0.0001	<0.0001	0.0003	0.3164	0.719
B4 vs. 300 PG (ng/µl)	<0.0001	<0.0001	0.0013	0.4151	0.8927

Supplementary Figure 18. Analysis of the growth (OD) and luciferase activity (RLU) in a WT *B. subtilis* strain harboring P_{srfAA}-*lux* (surfactin) reporter. Growth and luminescence were monitored in B4 medium (No Treatment), and B4 medium supplemented with different PG concentrations (25- 300 ng/µl) from *B. thuringiensis.* Graphs represent mean ± SD from three independent experiments (n = 9). Statistical analysis was performed using two-way ANOVA followed by Dunnett's multiple comparison test. *P* < 0.05 was considered statistically significant. P values at different time points are shown in the table. Source data are provided as a Source Data file.

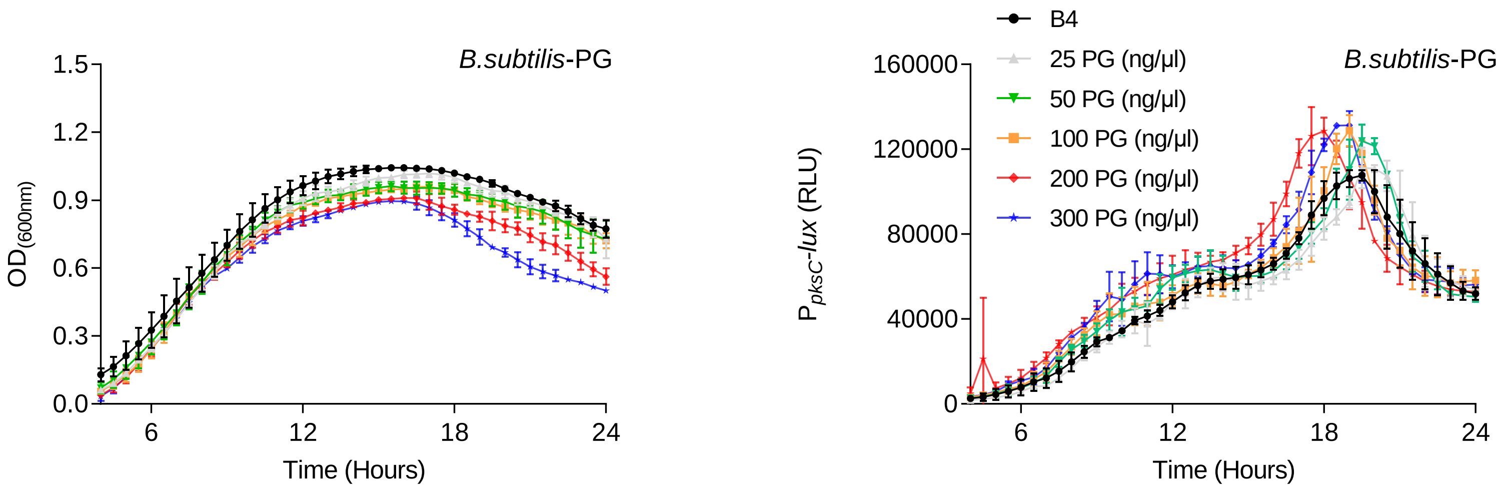

	6 Hours	12 Hours	18 Hours	24 Hours
B4 vs. 25 PG (ng/µl)	0.0299	0.1564	0.9482	0.3977
B4 vs. 50 PG (ng/µl)	0.2127	0.031	0.0324	0.3394
B4 vs. 100 PG (ng/µl)	0.0092	0.0067	0.0262	0.2132
B4 vs. 200 PG (ng/µl)	0.0138	<0.0001	<0.0001	<0.0001
B4 vs. 300 PG (ng/µl)	0.0115	<0.0001	<0.0001	<0.0001

	6 Hours	12 Hours	18 Hours	24 Hours
B4 vs. 25 PG (ng/µl)	0.9954	>0.9999	0.0304	0.9956
B4 vs. 50 PG (ng/µl)	>0.9999	0.1209	0.2436	0.9985
B4 vs. 100 PG (ng/µl)	0.9999	0.9723	0.9457	0.6487
B4 vs. 200 PG (ng/µl)	0.9698	0.0968	<0.0001	0.8858
B4 vs. 300 PG (ng/µl)	0.8731	0.0632	<0.0001	0.9997

Supplementary Figure 19. Analysis of the growth (OD) and luciferase activity (RLU) in a WT *B. subtilis* strain harboring P_{pksC}-*lux* (bacillaene) reporter. Growth and luminescence were monitored in B4 medium (No Treatment), and B4 medium supplemented with different PG concentrations (25- 300 ng/µl) from WT *B. subtilis*. Graphs represent mean ± SD from three independent experiments (n = 9). Statistical analysis was performed using two-way ANOVA followed by Dunnett's multiple comparison test. $P < 0.05$ was considered statistically significant. P values at different time points are shown in the table. Source data are provided as a Source Data file.

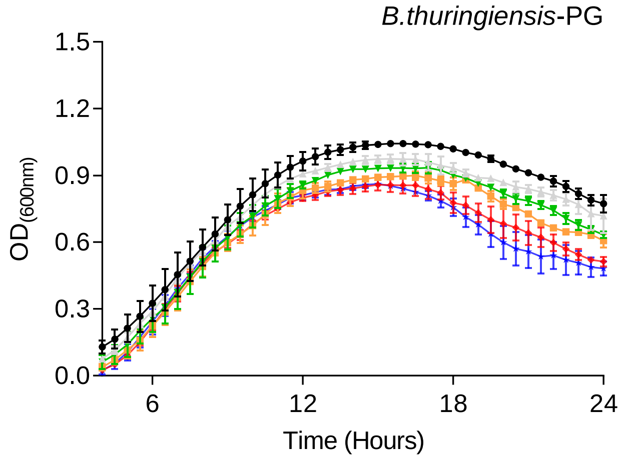

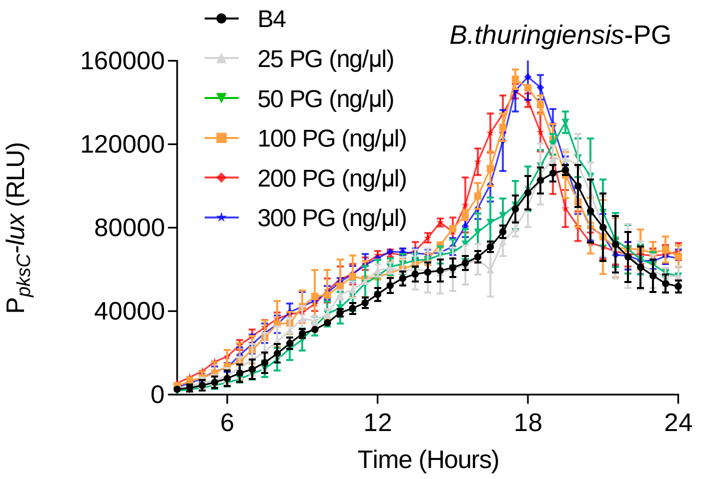

	6 Hours	12 Hours	18 Hours	24 Hours
B4 vs. 25 PG (ng/µl)	0.6041	0.1888	0.0183	0.2369
B4 vs. 50 PG (ng/µl)	0.0908	0.0017	0.0005	<0.0001
B4 vs. 100 PG (ng/µl)	0.0033	<0.0001	<0.0001	<0.0001
B4 vs. 200 PG (ng/µl)	0.0024	<0.0001	<0.0001	<0.0001
B4 vs. 300 PG (ng/µl)	0.025	<0.0001	<0.0001	<0.0001

	6 Hours	12 Hours	18 Hours	24 Hours
B4 vs. 25 PG (ng/µl)	0.9997	0.2574	0.7438	0.9112
B4 vs. 50 PG (ng/µl)	0.9982	0.5074	0.9984	0.9028
B4 vs. 100 PG (ng/µl)	0.8235	0.5439	<0.0001	0.0996
B4 vs. 200 PG (ng/µl)	0.8815	0.0298	<0.0001	0.1383
B4 vs. 300 PG (ng/µl)	0.323	0.0151	<0.0001	0.0404

Supplementary Figure 20. Analysis of the growth (OD) and luciferase activity (RLU) in a WT *B. subtilis* strain harboring P_{pksC}-*lux* (bacillaene) reporter. Growth and luminescence were monitored in B4 medium (No Treatment), and B4 medium supplemented with different PG concentrations (25- 300 ng/µl) from *B. thuringiensis*. Graphs represent mean ± SD from three independent experiments (n = 9). Statistical analysis was performed using two-way ANOVA followed by Dunnett's multiple comparison test. *P* < 0.05 was considered statistically significant. P values at different time points are shown in the table. Source data are provided as a Source Data file.

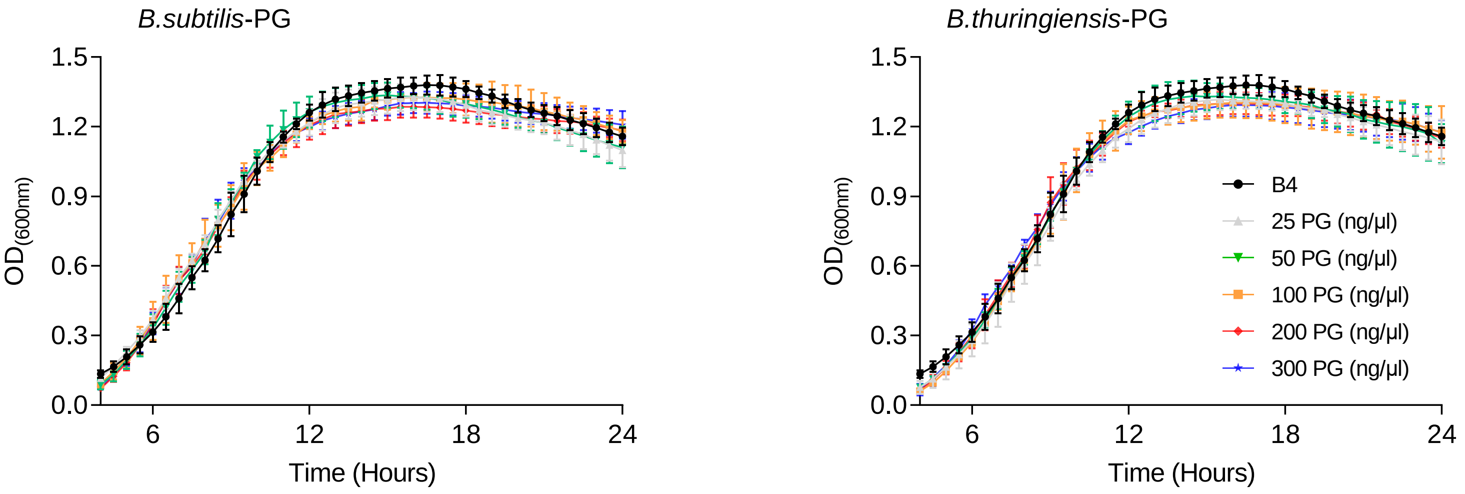

	6 Hours	12 Hours	18 Hours	24 Hours
B4 vs. PG-25 (ng/µl)	0.5078	0.7224	0.2968	0.4436
B4 vs. PG-50 (ng/µl)	0.9918	>0.9999	0.3784	0.6432
B4 vs. PG-100 (ng/µl)	0.6452	0.7088	0.6892	0.9753
B4 vs. PG-200 (ng/µl)	0.9036	0.5292	0.0874	0.9551
B4 vs. PG-300 (ng/µl)	0.826	0.3853	0.2632	0.5969

	6 Hours	12 Hours	18 Hours	24 Hours
B4 vs. PG-25 (ng/µl)	0.8797	0.3626	0.4694	0.9764
B4 vs. PG-50 (ng/µl)	0.9631	0.9932	0.6097	0.9332
B4 vs. PG-100 (ng/µl)	0.7902	0.9028	0.3657	0.9939
B4 vs. PG-200 (ng/µl)	0.9957	0.8301	0.3642	0.9998
B4 vs. PG-300 (ng/µl)	0.9988	0.1724	0.2482	0.9932

Supplementary Figure 21. Analysis of the growth (OD) in WT *B. subtilis* strain harboring quadruple deltion for all NRP Δ*srfAA,* Δ*pksC,* Δ*bacA and* Δ*ppsA* (Δ4). Growth was monitored in B4 medium (No Treatment), and B4 medium supplemented with different PG concentrations (25- 300 ng/µl) from the indicated *Bacilli*. Graphs represent mean ± SD from three independent experiments (n = 9). Statistical analysis was performed using two-way ANOVA followed by Dunnett's multiple comparison test. $P < 0.05$ was considered statistically significant. P values at different time points are shown in the table. Source data are provided as a Source Data file.

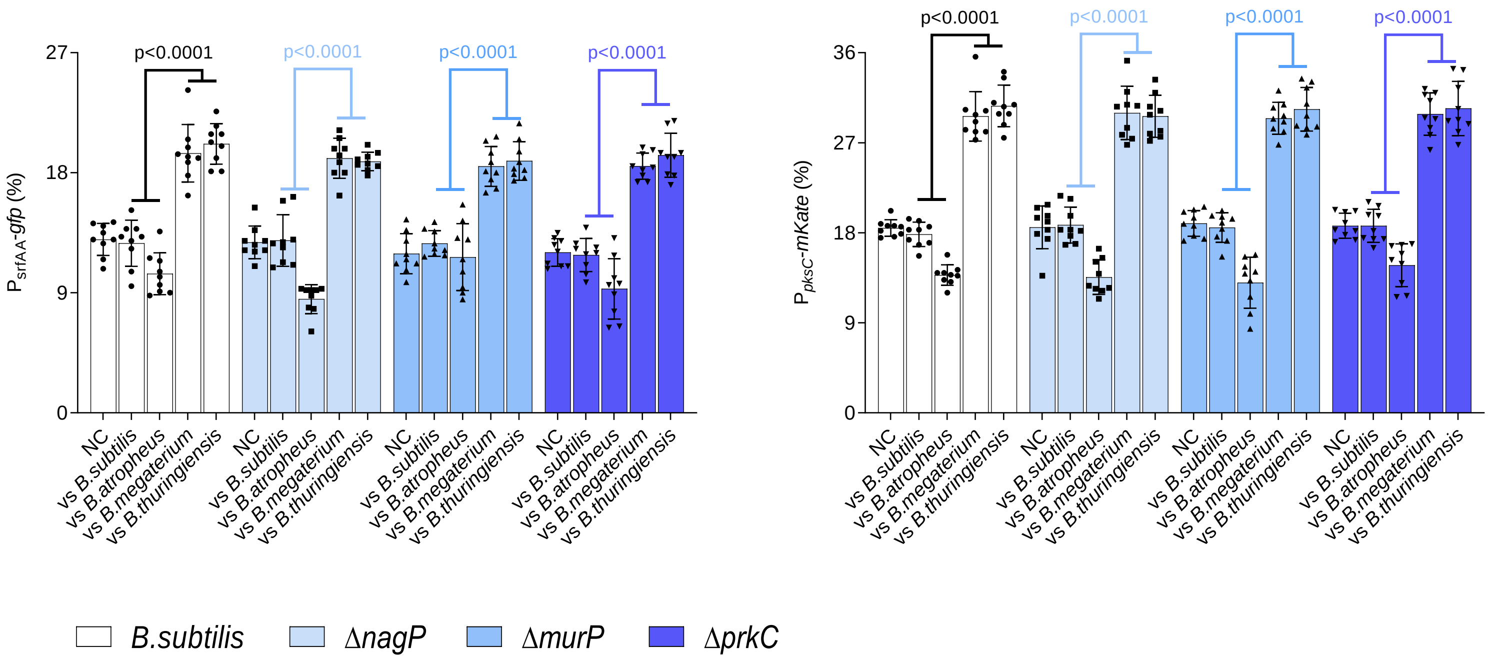

Supplementary Figure 22. A dual reporter WT *B. subtilis* strain harbouring P$_{srfAA}$-*yfp* (surfactin) and P$_{pksC}$-*mKate* (bacillaene) reporters and their indicated mutants were analyzed either alone (NC) or in competition against *Bacilli* using flow cytometry. Colonies were grown on B4 medium and incubated at 30°C. Data were collected from 24 h post inoculation; Y-axis represents the % of cells expressing the reporters, 100,000 cells were counted. Graphs represent mean ± SD from three independent experiments (n = 9). Statistical analysis was performed between resistant and sensitive members using Brown-Forsthye and Welch's ANOVA with Dunnett's T3 multiple comparisons test. $P < 0.05$ was considered statistically significant. Source data are provided as a Source Data file.

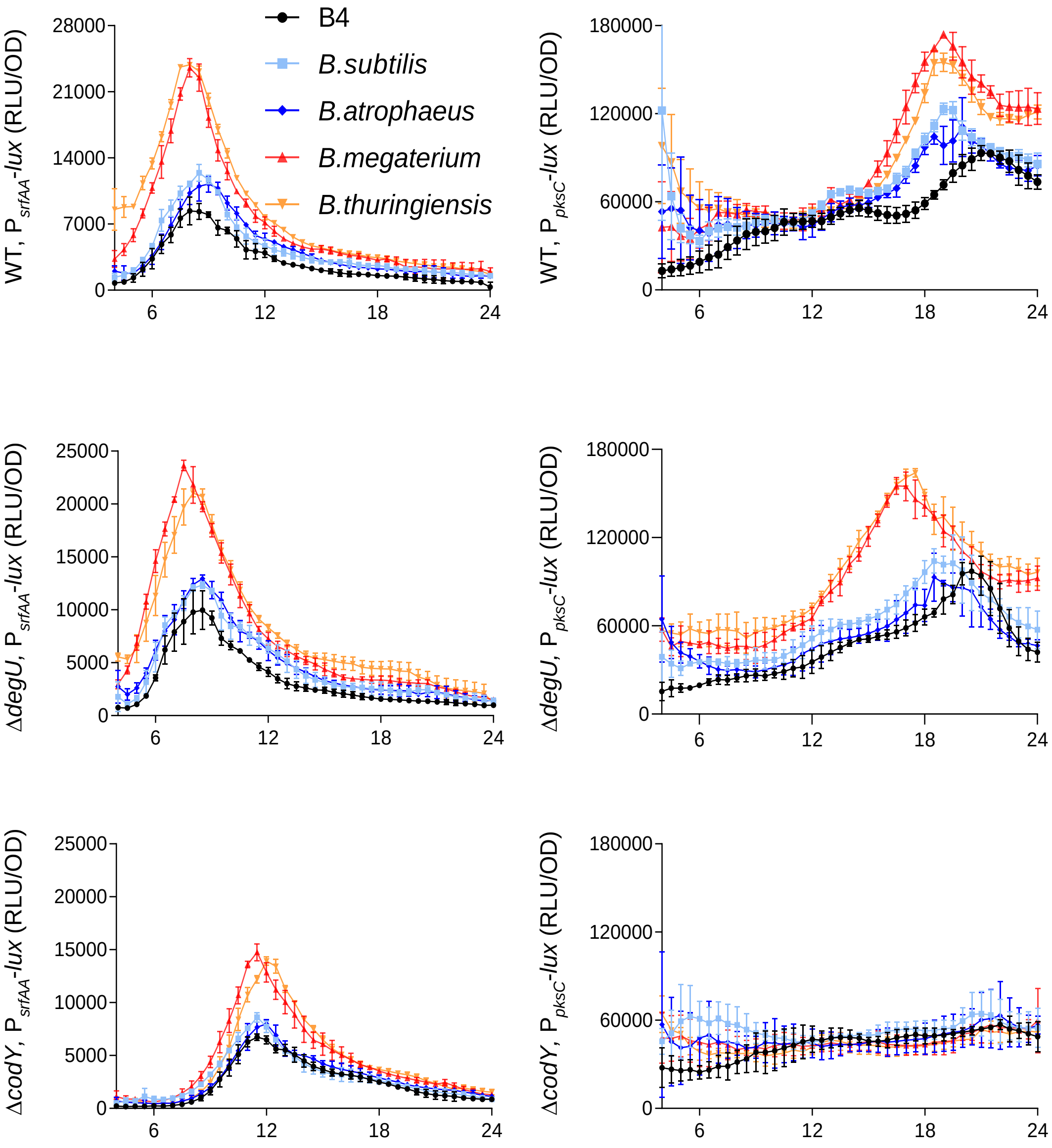

Supplementary Figure 23. Analysis of the luciferase activity in WT *B. subtilis* strain harboring P_{srfAA}-*lux* (surfactin) and P_{pksC}-*lux* (bacillaene) reporters and their indicated mutants. Luminescence was monitored in B4 medium (No Treatment), and B4 medium supplemented with PG (100 ng/µl) from the indicated species. Graphs represent mean ± SD from three independent experiments (n = 9). Statistical analysis was performed using two-way ANOVA followed by Dunnett's multiple comparison test. $P < 0.05$ was considered statistically significant. P values at different time points are shown in the Supplementary Data 1. Source data are provided as a Source Data file.

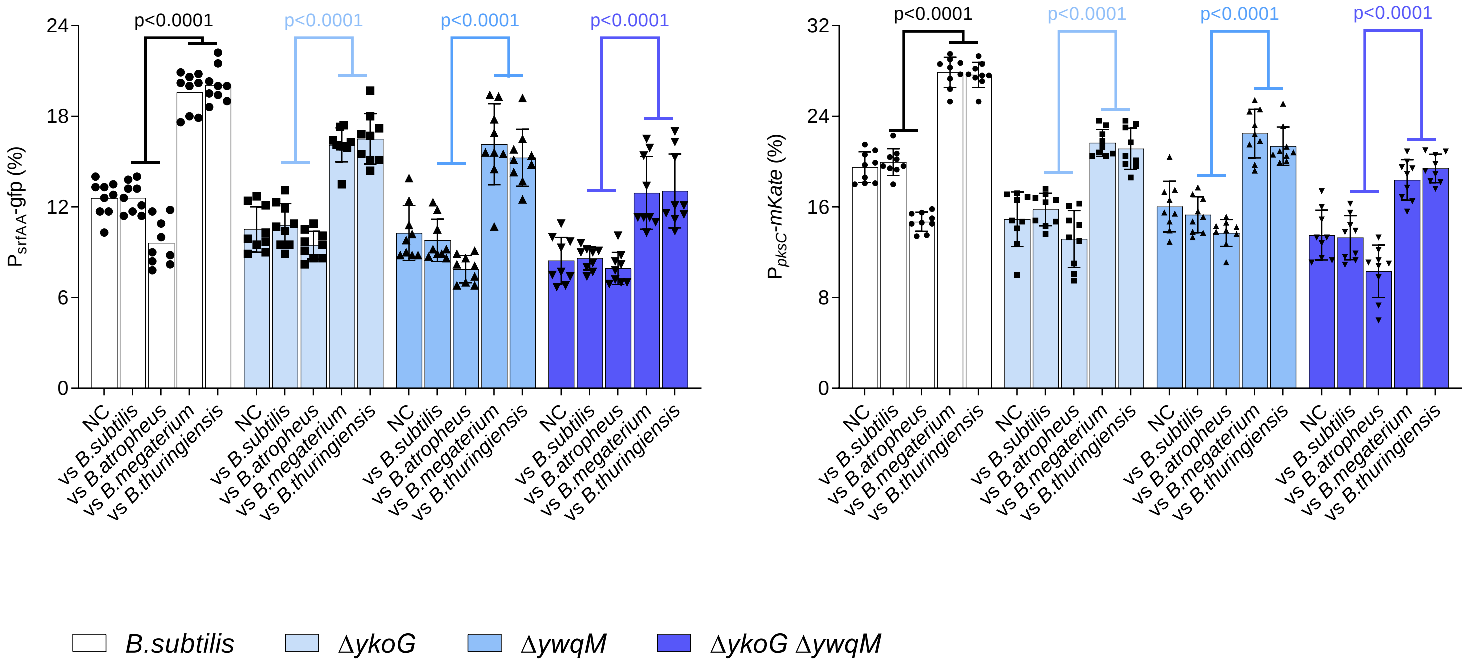

Supplementary Figure 24. A dual reporter WT *B. subtilis* strain harbouring P_{srfAA}-*yfp* (surfactin) and P_{pksC}-*mKate* (bacillaene) reporters and their indicated mutants were analyzed either alone (NC) or in competition against *Bacilli* using flow cytometry. Colonies were grown on B4 medium and incubated at 30°C. Data were collected from 24 h post inoculation; Y-axis represents the % of cells expressing the reporters, 100,000 cells were counted. Graphs represent mean ± SD from three independent experiments (n = 9). Statistical analysis was performed between resistant and sensitive members using Brown-Forsthye and Welch's ANOVA with Dunnett's T3 multiple comparisons test. $P < 0.05$ was considered statistically significant. Source data are provided as a Source Data file.

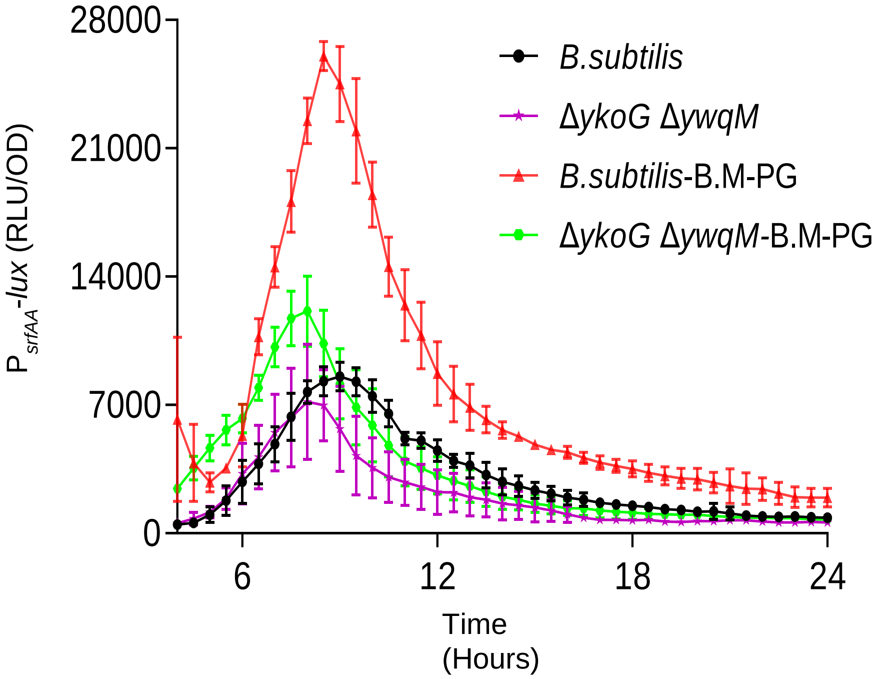

	6 Hours	8 Hours	12 Hours	18 Hours	24 Hours
B.subtilis vs. Δ*ykoG* Δ*ywqM*	0.9471	0.9178	0.0712	0.7822	0.9876
B.subtilis vs. *B.subtilis* -B.M-PG	0.0356	<0.0001	0.0001	0.1278	0.5774
B.subtilis vs. Δ*ykoG* Δ*ywqM*-B.M-PG	0.0023	<0.0001	0.4035	0.9685	0.9993

Supplementary Figure 25. Analysis of the luciferase activity in a WT *B. subtilis* strain harboring P_{srfAA}-*lux* (surfactin) reporter and its dual deletion mutant of Δ*ykoG* Δ*ywqM*. Luminescence was monitored in B4 medium (No Treatment), and B4 medium supplemented with PG (100 ng/µl) from *B.megaterium* (B.M-PG). Graphs represent mean ± SD from three independent experiments (n = 9). Statistical analysis was performed using two-way ANOVA followed by Dunnett's multiple comparison test. $P < 0.05$ was considered statistically significant. P values at different time points are shown in the table. Source data are provided as a Source Data file.

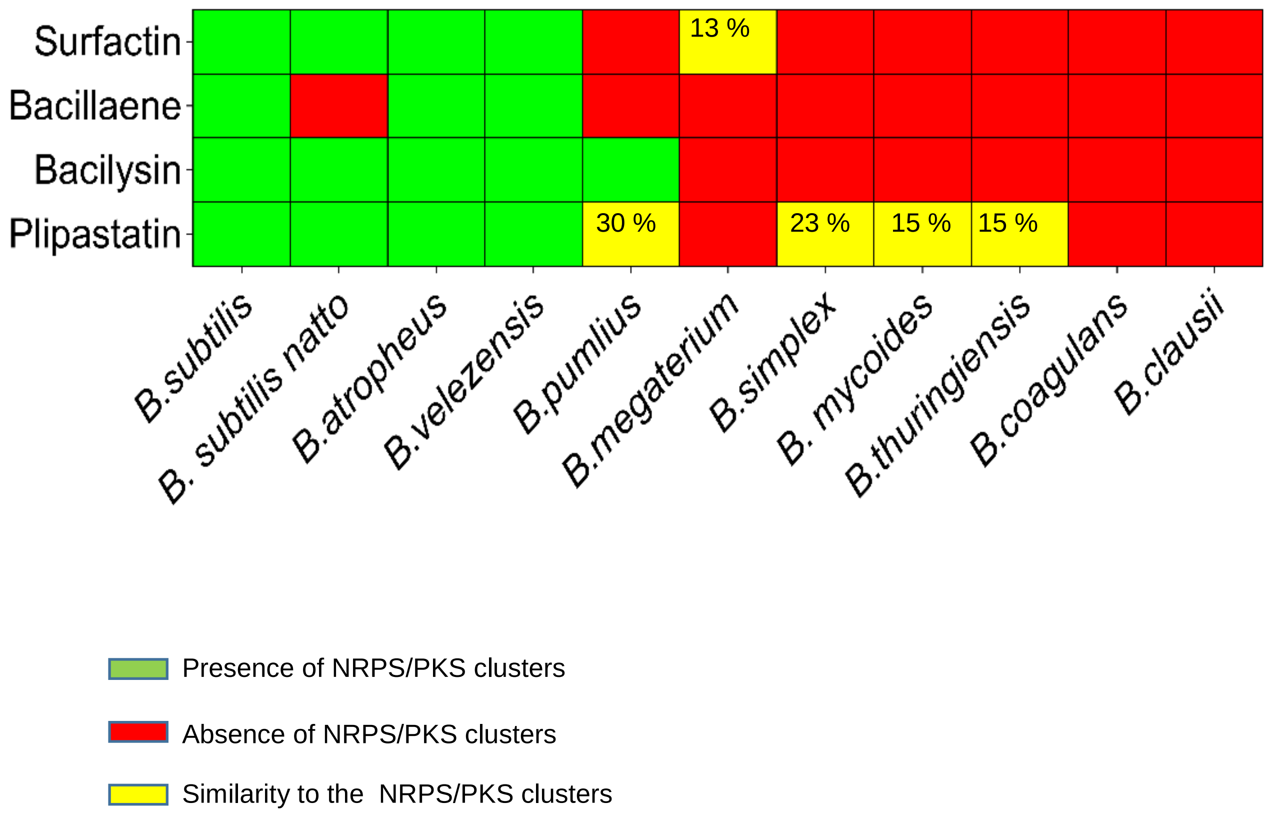

Supplementary Figure 26. Distribution of biosynthetic gene clusters of surfactin, bacillaene, bacilysin and plipastatin in different *Bacilli* used in this study. Analysis was performed using antiSMASH 6.0.1.using default parameters. For strains marked with * full genome sequences were not available, for such strains members with full genome sequences from the same species were used for the analysis (*B.megaterium* Q3, *B.coagulans* DSM 1 = ATCC 7050 and *Bacillus clausii* DSM 8716T).

Flow cytometry gating strategy

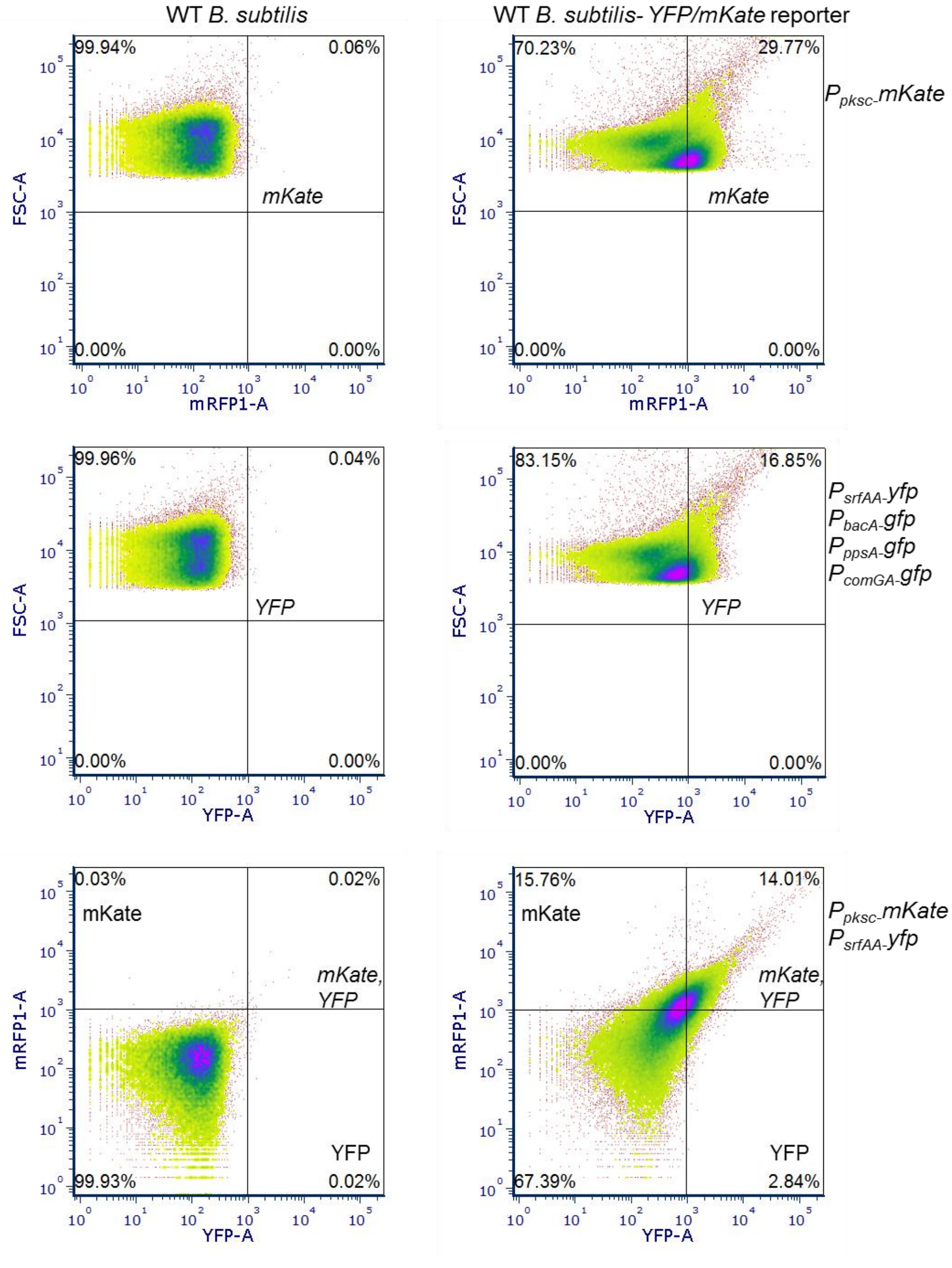

Supplementary Figure 27. Gating strategy for flow cytometry analysis used in the figures 4a, 4b , 4d, 7c, 7el, 7er and Supplementary figures 22 and 24. WT *B. subtilis* was used as a control to separate self-fluorescence from true fluorescent population of the reporters. Quadrants with population (%) of the indicated reporters are mentioned.

Supplementary Table 1

	vs WT	vs Δ*srf*	vs Δ*pks*	vs Δ*bac*	vs Δ*pps*	vs Δ*srf* Δ*pks*	vs Δ*srf* Δ*bac*	vs Δ*srf* Δ*pps*	vsΔ*pks* Δ*bac*	vsΔ*pks* Δ*pps*	vsΔ*bac* Δ*pps*	Δ4
B. subtilis	1.17	1.02	1.01	1.09	1.07	0.98	1.01	0.96	1.17	1.18	1.08	1.03
B. subtilis natto	1.41	1.07	1.21	1.29	1.18	1.10	1.29	1.30	1.07	1.27	1.18	0.98
B. atrophaeus	1.18	1.20	1.04	1.30	1.26	1.07	1.08	1.22	1.17	1.30	1.22	0.99
B. velezensis	0.94	1.02	1.10	0.85	1.00	1.09	1.02	1.03	1.00	1.08	1.08	0.96
B. pumilus	13.25	8.89	7.87	12.78	13.31	4.47	7.53	8.22	8.29	8.12	12.76	1.46 (p=0.018)
B. clausii	13.61	7.61	9.19	12.96	12.77	3.91 (p=0.001)	7.14	8.27	7.66	7.72	12.88	0.92 (p<0.0001)
B. coagulans	20.86	14.40	16.34	19.38	20.49	4.68 (p=0.0003)	10.95	12.80	12.95	12.09	19.42	0.93 (p<0.0001)
B. megaterium	44.22	26.31 (p=0.003)	29.29 (p=0.020)	35.85	41.35	7.89 (p<0.0001)	17.66 (p<0.0001)	22.83 (p=0.0003)	18.00 (p<0.0001)	20.63 (p<0.0001)	37.60	1.19 (p<0.0001)
B. mycoides	49.26	35.87	36.60	43.69	46.63	16.33 (p=0.0014)	31.46	35.00	32.42	35.53	42.24	5.67 (p<0.0001)
B. simplex	55.78	35.55	34.91	47.28	54.06	13.52 (p<0.0001)	32.55	37.46	32.84	36.41	46.54	2.42 (p<0.0001)
B. thuringiensis	66.63	47.34 (p=0.0006)	45.89 (p=0.0002)	57.17	61.11	19.01 (p<0.0001)	36.57 (p<0.0001)	39.41 (p<0.0001)	33.32 (p<0.0001)	37.15 (p<0.0001)	53.26 (p=0.031)	5.08 (p<0.0001)

Supplementary Table 1. Table comparing the toxicity exhibited by WT *B. subtilis* and its NRPs/PKS deletion on the indicated *Bacilli* used in this studies. The data were obtained from Figure 1, 2 and S3. Toxicity values (shown in black) are means from three independent experiments (n = 9). Statistical analysis was performed using one-way ANOVA followed by Tukey's multiple comparison post hoc testing. $P < 0.05$ was considered statistically significant. Significant differences between the toxicity towards indicated *Bacilli* when competed against WT *B. subtilis* and toxicity towards indicated *Bacilli*, when competed against the indicated NRP/PKS mutants are shown by their respective p values (in blue).

Supplementary Table 2

Expected m/z	Observed m/z	Mass error (ppm)	Observed RT (min)	Observed drift (ms)	Ion Formula	Observed CCS (Å^2)	Feature
432.2533	432.2541	1.83	26.29	7.44	C28H34NO3		
251.1430	251.1430	-0.03	26.28	6.89	C18H19O		
563.3479	563.3484	0.7	26.29	7.29	C34H47N2O5		-OH
603.3405	603.3406	0.16	26.29	7.27	C34H48N2O6Na	246.07	+Na
581.3585	581.3582	-0.52	26.29	7.19	C34H49N2O6	244.11	+H

Supplementary Table 2. Bacillaene was putatively identified using adducts, fragmentation, and mass accuracy. Identified mass signals associated with bacillaene are presented in the table.

Supplementary Table 3- Strains

Strain	Description	Source or reference
B. subtilis	Wild type	[1]
B. subtilis PY79	Wild type	[2]
B. subtilis natto	Wild type	Rotem Sorek lab, WIS, Israel
B. atrophaeus 1942	Wild type	Rotem Sorek lab, WIS, Israel
B. velezensis FZB42	Wild type	Rotem Sorek lab, WIS, Israel
B. pumilus ATCC 70161	Wild type	Rotem Sorek lab, WIS, Israel
B. megaterium isolate VS1	Wild type	Ayelet Fishman Lab, Technion, Israel
B. simplex SHB26	Wild type	Lab stock
B. mycoides AH621	Wild type	Rotem Sorek lab, WIS, Israel
B. thuringiensis subspecies kurstaki strain HD73	Wild type	Rotem Sorek lab, WIS, Israel
B. coagulans ATCC 10545	Wild type	BGSC 61A2
B. clausii domuvar	Wild type	BGSC 17A1
Δ*srfAA-srfAD (*Δ*srfAA)*	*B. subtilis* Δ*srfAA*::Em^r deficient in surfactin synthesis	[3]
Δ*pksC-pksR (*Δ*pks)*	*B. subtilis* Δ*pks*::Sp^r deficient in bacillaene synthesis	[4]
ΔbacC (Δ*bac*)	*B. subtilis* Δ*bac*::Kan^r deficient in bacilysin synthesis	This study

Δ*ppsA* (*Δpps*)	*B. subtilis* Δ*pps*::Tc^r deficient in plipastatin synthesis	This study
Δ*srfAA* Δ*pks*	*B. subtilis* Δ*srfAA*::Em ^{r}, Δ*pks*::Sp^r, deficient in surfactin and bacillaene synthesis.<br>DNA was extracted from strain Δ*srfAA*::Em^r and transferred into strain Δ*pks*::Sp^r, as described in materials and methods	This study
Δ*srfAA* Δ*bac*	*B. subtilis* Δ*srfAA*::Em ^{r}, Δ*bac*::Kan ^{r}, deficient in surfactin and bacilysin synthesis<br>DNA was extracted from strain Δ*srfAA*::Em^r and transferred into strain Δ*bac*::kan^r, as described in materials and methods	This study
Δ*srfAA* Δ*pps*	*B. subtilis* Δ*srfAA*::Em^r, Δ*pps*::Tc^r, deficient in surfactin and plipastatin synthesis<br>DNA was extracted from strain Δ*srfAA*::Em^r and transferred into strain Δ*pps*::Tc^r, as described in materials and methods	This study
Δ*pks* Δ*bac*	*B. subtilis* Δ*pks*::Sp^r, Δ*bac*::kan^r ,deficient in bacillaene and bacilysin synthesis<br>DNA was extracted from strain Δ*pks*::Sp^r and transferred into strain Δ*bac*::kan^r, as described in materials and methods	This study
Δ*pks* Δ*pps*	*B. subtilis* Δ*pks*::Sp^r, Δ*pps*::Tc^r, deficient in bacillaene and plipastatin synthesis<br>DNA was extracted from strain Δ*pks*::Sp^r and transferred into strain Δ*pps*::Tc^r, as described in materials and methods	This study
Δ*bac* Δ*pps*	*B. subtilis* Δ*bac*::Kan^r, Δ*pps*::Tc^r, deficient in bacilysin and plipastatin synthesis	This study

	DNA was extracted from strain Δ*bac*::Kan^r and transferred into strain Δ*pps*::Tc^r, as described in materials and methods	
Δ*srfAA* Δ*pks* Δ*bac* Δ*pps* (Δ4)	*B. subtilis* Δ s*rfAA*::Em^r, Δ*pks*::Sp^r, Δ*bac*::kan^r, Δ*pps*::Tc ^{r}, deficient in surfactin, bacillaene, bacilyisn and plipastatin synthesis	This study
P_{srfAA}-*yfp*	*B. subtilis sacA*:: P_{srfAA}-*yfp* (Sp ^{r}), promoter of surfactin operon tagged to the YFP reporter integrated in the neutral *amyE* locus	Avigdor Eldar Lab, TAU, Israel
P_{bacA}-*gfp*	*B. subtilis amyE*:: P_{bacA}-*gfp* (Cm ^{r}), promoter of bacilysin operon tagged to the GFP reporter integrated in the neutral *amyE* locus	This study
P_{ppsA}-*gfp*	*B. subtilis amyE*:: P_{ppsA}-*gfp* (Cm ^{r}), promoter of plipstatin operon tagged to the GFP reporter integrated in the neutral *amyE* locus	This study
P_{srfAA}-*yfp*, P_{pksC}-*mKate*	*B. subtilis sacA*:: P_{srfAA}-*yfp* (Sp ^{r}), *pyrD*:: P_{pksC}-*mKate* (Cm ^{r}), double reporter to monitor expression of surfactin and bacillaene	This study
P_{ComGA}-*gfp*	*B. subtilis amyE*:: P_{comGA}-*gfp* (Cm ^{r}), promoter of bacilysin operon tagged to the GFP reporter integrated in the neutral *amyE* locus	Lab stock
P_{pksC}-*lux*	*B. subtilis* sacA::P_{pksC}-*lux* (Cm r, Sp ^{r}), promoter of bacillaene operon tagged to the luciferase reporter integrated in the neutral *SacA* locus	[5]
P_{srfAA}-*lux*	*B. subtilis* sacA::P_{srfAA}-*lux* (Cm r, Sp ^{r}), promoter of surfactin operon tagged to the	This study

	luciferase reporter integrated in the neutral *sacA* locus	
Δ*murP*	DNA was extracted from *B. subtilis* 168 Δ*murP*, and transferred into *B. subtilis* 3610 as described in materials and methods. Kan ^{r}	[6]
Δ*murP*, P_{srfAA}-*yfp*, P_{pksC}-*mKate*	DNA was extracted from *B. subtilis* Δ*murP*, and transferred into *B. subtilis* P_{srfAA}-*yfp*, P_{pksC}-*mKate* as described in materials and methods.	This study
Δ*nagP*	DNA was extracted from *B. subtilis* 168 Δ*nagP*, and transferred into *B. subtilis* 3610 as described in materials and methods. Kan ^{r}	[6]
Δ*nagP*, P_{srfAA}-*yfp*, P_{pksC}-*mKate*	DNA was extracted from *B. subtilis* Δ*nagP*, and transferred into *B. subtilis* P_{srfAA}-*yfp*, P_{pksC}-*mKate* as described in materials and methods.	This study
Δ*prkC*	DNA was extracted from *B. subtilis* 168 Δ*prkC*, and transferred into *B. subtilis* 3610 as described in materials and methods. Kan ^{r}	[6]
Δ*prkC*, P_{srfAA}-*yfp*, P_{pksC}-*mKate*	DNA was extracted from *B. subtilis* Δ*prkC*, and transferred into *B. subtilis* P_{srfAA}-*yfp*, P_{pksC}-*mKate* as described in materials and methods.	This study
Δ*comA*	DNA was extracted from *B. subtilis* 168 Δ*comA*, and transferred into *B. subtilis* 3610 as described in materials and methods. Kan ^{r}	[6]
Δ*comA*, P_{srfAA}-*lux*	DNA was extracted from *B. subtilis* Δ*comA*, and transferred into *B. subtilis* P_{srfAA}-*lux* as described in materials and methods.	This study
Δ*comA*, P_{pksC}-*lux*	DNA was extracted from *B. subtilis* Δ*comA*, and transferred into *B. subtilis* P_{pksC}-*lux* as described in materials and methods.	This study

Δ*comA,* P_{srfAA}-*yfp,* P_{pksC}-*mKate*	DNA was extracted from *B. subtilis* Δ*comA*, and transferred into *B. subtilis* P_{srfAA}-*yfp,* P_{pksC}-*mKate* as described in materials and methods.	This study
Δ*comA,* P_{bacA}-*gfp*	DNA was extracted from *B. subtilis* Δ*comA*, and transferred into *B. subtilis* P_{bacA}-*gfp* as described in materials and methods.	This study
Δ*comA,* P_{ppsA}-*gfp*	DNA was extracted from *B. subtilis* Δ*comA*, and transferred into *B. subtilis* P_{ppsA}-*gfp* as described in materials and methods.	This study
Δ*spo0A*	*B. subtilis* Δ*spo0A*::Sp^r	[5]
Δ*spo0A,* P_{srfAA}-*lux*	DNA was extracted from *B. subtilis* Δ*spo0A*, and transferred into *B. subtilis* P_{srfAA}-*lux* as described in materials and methods.	This study
Δ*spo0A,* P_{pksC}-*lux*	DNA was extracted from *B. subtilis* Δ*spo0A*, and transferred into *B. subtilis* P_{pksC}-*lux* as described in materials and methods.	This study
Δ*codY*	*B. subtilis* Δ*codY*::Kan^r	[5]
Δ*codY,* P_{srfAA}-*lux*	DNA was extracted from *B. subtilis* Δ*codY*, and transferred into *B. subtilis* P_{srfAA}-*lux* as described in materials and methods.	
Δ*codY,* P_{pksC}-*lux*	DNA was extracted from *B. subtilis* Δ*codY*, and transferred into *B. subtilis* P_{pksC}-*lux* as described in materials and methods.	[5]
Δ*degU*	*B. subtilis* Δ*degU*::Tc^r	[7]
Δ*degU,* P_{srfAA}-*lux*	DNA was extracted from *B. subtilis* Δ*degU*, and transferred into *B. subtilis* P_{srfAA}-*lux* as described in materials and methods.	This study
Δ*degU,* P_{pksC}-*lux*	DNA was extracted from *B. subtilis* Δ*degU*, and transferred into *B. subtilis* P_{pksC}-*lux* as described in materials and methods.	This study

Δ*ykoG*	DNA was extracted from *B. subtilis* 168 Δ*ykoG*, and transferred into *B. subtilis* 3610 as described in materials and methods. Kan ^{r}	6
Δ*ykoG*, P_{srfAA}-*yfp*, P_{pksC}-*mKate*	DNA was extracted from *B. subtilis* Δ*ykoG*, and transferred into *B. subtilis* P_{srfAA}-*yfp*, P_{pksC}-*mKate* as described in materials and methods.	This study
Δ*ywqM*, P_{srfAA}-*yfp*, P_{pksC}-*mKate*	DNA was extracted from *B. subtilis* Δ*ywqM*, and transferred into *B. subtilis* P_{srfAA}-*yfp*, P_{pksC}-*mKate* as described in materials and methods.	This study
Δ*ykoG* Δ*ywqM*, P_{srfAA}-*yfp*, P_{pksC}-*mKate*	DNA was extracted from strain Δ*ywqM*::Tc^{r} and Δ*ykoG*::kan^{r} and transferred into strain P_{srfAA}-*yfp*, P_{pksC}-*mKate* as described in materials and methods	This study
Δ*ykoG* Δ*ywqM*, P_{srfAA}-*lux*	DNA was extracted from strain Δ*ywqM*::Tc^{r} and Δ*ykoG*::kan^{r} and transferred into strain P_{srfAA}-*lux* as described in materials and methods	This study

Gene names and species name are in italics.

Δ- deletion mutant

Kan ^{r} – kanamycin resistance, **Tc** ^{r} – tetracycline resistance, **Cm** ^{r} – chloramphenicol resistance, **Sp** ^{r} – spectinomycin resistance , **Lnc** ^{r} – lincomycin resistance , **Ery** ^{r} – erythomycin resistance

Supplementary Table 4

Plasmids used in the study

Plasmid	Description	Source
pDG780	Cloning vector, kan^r	[8]
pDG1515	Cloning vector, Tc^r	[8]
pYC121	Cloning vector, Cm^r	[9]
pPyr-Cm- mKate	Cloning vector, Cm^r	This study
pBS3C-lux	Cloning vector, Cm^r	[5]

Kan ^{r} – kanamycin resistance, **Tc** ^{r} – tetracycline resistance, **Cm** ^{r} – chloramphenicol resistance

Supplementary Table 5

Primes used in the study

Strains	Primers
Δ*bacC*	Forward up 5'- GCAGCACATACTTTGCCGTCAGCTTCGCAT - '3<br>Reverse up 3'- CAATTCGCCCTATAGTGAGTCG AAGGAGTGTTTTACATATGGAGAGAA - '5<br>Forward dw 5'- CCAGCTTTTGTTCCCTTTAGTGAG GGTTTTATCGGTGAGGTTCATGA - '3<br>Reverse dw 3'- CAGAGACGATATGCTTGATACATCTG - '5
Δ*ppsA*	Forward up 5' - GAGAGCATGGAACAAACACGATTACAATTGAAGGGAGCGT - '3<br>Reverse up 3'- CAATTCGCCCTATAGTGAGTCGTCGGATTCCCTCCAGTTCTCA - '5<br>Forward dw 5'- CCAGCTTTTGTTCCCTTTAGTGAGAGCGGATTAGCGGACAGAGGCCA - '3<br>Reverse dw 3'- CCACGCGTGAAATTCCAAATTTCGTTTATGGG - '5
ΔywqM	Forward up 5' – AGCGATCGGCTGGGTCCAATCG - '3<br>Reverse up 3'- CAATTCGCCCTATAGTGAGTCGTCAGCTGCTTCAATTCCAC- '5<br>Forward dw 5'- CCAGCTTTTGTTCCCTTTAGTGAG AACCTCTCCACAAGGACGAC - '3<br>Reverse dw 3'- CTAGAATCGAGAAGAGAGACTCAC - '5
P_{pksC}-*mKate*	Forward 5' - GGATCCTAGAAGCTTATCAAATCGCCCGGCCATTCGA - '3<br>Reverse 3' - CTTAATCAGCTCGCTCACCATTCTCTCAAAGCCACCCTTC- '5
P_{bacA}-*gfp*	Forward 5' - ATAAGGGTAACTATTGCCGACAAAGTTTCTAAATTCCTAT- '3<br>Reverse 3' - AAGTTCTTCTCCTTTACTCATGAGCACCAACCAATCTTTTA - '5
P_{ppsA}-*gfp*	Forward 5' - AAGGGTAACTATTGCCGACTGTAATAACGCTTTGTC- '3<br>Reverse 3' - GTTCTTCTCCTTTACTCATCGGATTCCCTCCAGTTCTCATAATAA - '5
P_{srfAA}-*lux*	Forward 5' - GGTCGACAGGAGGACTCTCTCGTTGTAAGACGCTCTTCGC- '3<br>Reverse 3' - AAGTTTCCAAATTTCATATTGTCATACCTCCCCTAA - '5

Gene names in italics.

Supplementary Methods

Strain construction

Deletions mutants were constructed using standard methods as described in [10,11]. For polymerase chain reactions, plasmids and primers used in this study are listed in Supplementary Table 4 and 5 respectively. Briefly genomic regions of around 1000 bp upstream and downstream of NRP operon were amplified from *B. subtilis* NCIB 3610 (Wild Type, WT) chromosomal DNA. Deletions were generated using the long-flanking homology (LFH) PCR mutagenesis protocol of [12], replacing an endogenous locus with a resistance gene from either pDG1515 (Δ*pps* and Δ*ywqM*) and pDG780 (Δ*bac*). DNA was extracted with the Wizard Genomic DNA Purification Kit (Promega) transformed into strain *B. subtilis* PY79. Genomic DNA of the recipient strain was transformed into WT *B. subtilis* NCIB 3610 or deletion mutants [13]. All mutant strains were confirmed for accurate integration by PCR. Restriction enzymes and Phusion HF DNA Polymerase were purchased from New England BioLabs.

All cloning experiments were performed with *B. subtilis* NCIB 3610 (Wild Type, WT) and *E. coli* DH5α using restriction free cloning [14]. pYC121 plasmid was used as template for P_{bacA}-*gfp* (bacilysin) and P_{ppsA}-*gfp* (plipastatin) and plasmid pPyr-Cm – mKate for P_{pksC}-*mKate*. Briefly, PCR fragments of promoter regions were amplified from *B. subtilis* chromosomal DNA, using primers listed in Supplementary Table 5 and ligated into their respective plasmids (Supplementary Table 4). The ligated plasmids were then transformed into *E. coli* DH5α. Positive reporters were inserted by using double homologous recombination into neutral integration sites (*amyE*) and (*pyrD*) in the genome of WT *B. subtilis* by inducing natural competence [13] and confirmed by PCR. Selective media for cloning purposes were prepared with LB broth or LB-agar. WT *B. subtilis* P_{srfAA}-*yfp* was a kind gift from the lab of Avigdor Eldar, TAU Israel.

To create double-labelled strain of WT *B. subtilis* P_{pksC}-*mKate,* P_{srfAA}-*yfp,* genomic DNA from WT *B. subtilis* P_{srfAA}-*yfp* was integrated into neutral integration sites (*sacA*) in the genome of WT *B. subtilis* P_{pksC}-*mKate.* Dually labelled strains were confirmed for successful transformation by PCR. Selective media for cloning purposes were prepared with LB broth or LB-agar using antibiotics at the following final concentrations:10 µg/ml chloramphenicol (Amersco) and 10 µg/ml spectinomycin (Tivan biotech).

To create double-deletion or quadruple deletion (Δ4) strains, DNA was extracted with the Wizard Genomic DNA Purification Kit (Promega) transformed into strain *B. subtilis* PY79. Genomic DNA of the recipient was transformed into WT *B. subtilis* NCIB 3610 or deletion mutants in the genome of WT *B. subtilis* by inducing natural competence [13]. All mutant strains were confirmed for accurate integration by PCR. For the generation of the Luminescence reporter P_{srfAA}-*lux* we performed restriction free cloning [14] using the plasmid pBS3C-lux [15], which contains a functional luciferase operon (*luxABCDE*). Briefly, promoter region was amplified from WT *B. subtilis* chromosomal DNA, using primers listed in Supplementary Table 3 and ligated into plasmid. The ligated plasmids were then transformed into *E. coli* DH5α. Positive reporters were inserted by using double homologous recombination into neutral integration sites (*sacA*) in the genome of WT *B. subtilis* by inducing natural competence [13]. Selective media for cloning purposes were prepared with LB broth or LB-agar using antibiotics at the following final concentrations: 10 μg/ml chloramphenicol (Amersco).

Constructing of pPyr-Cm - mKate plasmid

Primer pairs for generating DNA fragments with varying overlapping ends by PCR were designed using SnapGene Viewer program. Briefly, mKate gene was first amplified from pDR183 using primers mKate forward - 5'-GGATCCTAGAAGCTTATCAAATCGCCCGGCCATTCGA - '3, mKate reverse 3'-CTGTCAAACATGAGAATTCGTCATCTGTGCCCCAGTTTGC - '5 , pPyr-Cm backbone was amplified using primers vector forward 5'-GCAAACTGGGGCACAGATGACGAATTCTCATGTTTGACAG - '3, vector reverse 3'-TAATCAGCTCGCTCACCATATAAGCTTCTAGGATCCTGAG - '5. The PCR products were then assembled using NEBuilder® HiFi DNA Assembly Master Mix, based on manufacture protocol and the reaction mix was incubated for 50°C. Following incubation, the Gibson assembly products were then treated with DpnI digestion the transferred into competent *E. coli* DH5α cells. Positive reporters were selected and the insertion of mKate was confirmed by PCR. Selective media for cloning purposes were prepared with LB broth or LB-agar using antibiotics 10 μg/ml chloramphenicol (Amersco)

Extraction of DNA, EPS, PG and Protease Treated Conditioned Medium

DNA was extracted using Wizard® Genomic DNA Purification Kit. Spectrophotometric analysis was performed using NanoDrop spectrophotometer and DNA was stored at −80 °C for further use.

EPS was extracted [16] from the each indicated *Bacilli* biofilms grown at 48 hours at 30°C in B4 medium . Nine biofilm colonies from each *Bacilli* were scrapped and suspended in phosphate-buffered saline (137 mM NaCl, 2.7 mM KCl, 10 mM Na_2HPO_4, 1.8 mM KH_2PO_4), were mildly sonicated, and were then centrifuged at 8,000 × g to remove the cells. The supernatant was collected and mixed with five volumes of ice-cold isopropanol and incubated overnight at 4°C. Samples were then centrifuged at 10,000 × g for 10 min at 4°C. Pellets were suspended in a digestion mix of 0.1 M $MgCl_2$, 0.1 mg/ml of DNase, and 0.1 mg/ml of RNase, and were incubated for 4 h at 37°C. Samples were extracted twice with phenol-chloroform. The aquatic fraction was dialyzed for 48 h with Slide-A-Lyzer dialysis cassettes by Thermo Fisher, 3,500 molecular weight cut-off, against distilled dH_2O. Samples were lyophilized and lyophilized fraction was dissolved in 100 µl of dH_2O. The fractions were stored at −80 °C for further use.

Peptidoglycan (PG) was extracted from the indicated *Bacilli* in the corresponding figures legends [17,18]. *Bacilli* were grown in volume three hundred milliliter of B4 medium for 24 hours. Cells were collected by centrifugation (10,000 × g), washed with 0.8% NaCl, resuspended in hot 4% SDS, boiled for 30 min, and incubated at room temperature (RT) overnight. The suspension was then centrifuged (10,000 × g) to collect the pellet, and washed five times with dH_2O to remove SDS. The pellet was then suspended in 25 mL 100 mM Tris–HCl, pH 7.5. with an addition of 25 µL RNAse solution (10 mg/mL), 25 µL of DNAse solution (10 mg/mL), and 250 µL of 1 M $MgSO_4$ and incubated for 4h at 37 °C, with gentle shaking. This was followed by treatment with 25 µL of trypsin (10 mg/mL) and 250 µL of 1 M $CaCl_2$ and incubated at 37 °C for 16 h, with gentle shaking. The insoluble material was then centrifuged at at 8,000 × g for 10 min at room temperature, washed once with water. The material was again resuspended in 4% SDS, boiled for 30 min, and incubated at room temperature (RT) overnight. The material was washed five times with dH_2O to remove SDS. Pellet containing PG was dried, weighted and dissolved in dH_2O. Material was further stored at −20 °C for further use

.

Protease Treated Conditioned Medium (CM)- Cells were grown to a mid-logarithmic phase of growth (OD=0.6-0.8). Cells were diluted 1:100 in 300 ml of B4 medium and grown at 30°C for 24 h in a shaker incubator (Brunswick™ Innova® 42). Cells were removed by a centrifugation at (8,000 × g) and the growth media was filtered by 0.22μm filter (Corning). For proteinase treatment, proteinase K (Sigma) was added to conditioned medium at a final concentration of 100 μg/ml and incubated for 2 hours at 37 °C, proteinase K was then removed with a 10-kDa cutoff spin filter

Detecting the presence of PG using ELISA

Detection of PG was carried out using Peptidoglycan ELISA kit (Abbexa®, Oxford, UK) according to the manufactures protocol. Briefly, *B. thuringiensis* biofilm colony was grown on B4 agar plates for 48 hours at 30 °C. 1.5 mm agar rings surrounding the colony were cut and scrapped out, and dissolved in of PBS (three agar rings were dissolved in 1 ml of PBS/ per observation) for 2 hours at room temperature by moderate shaking. Positive controls were prepared at 100 ng/ml as described and provided by the manufactures. For negative control Standard Diluted buffers were used as provided by the manufacturer. The suspensions from PBS, positive and negative controls (50μl each) were then transferred to the 96-well ELISA plate, followed by instructions provided by the manufacturer. OD was measured at 450nm using a microplate reader (Synergy 2; BioTek, Winooski, VT, USA).

Supplementary References

1. Branda, S. S., Gonzalez-Pastor, J. E., Ben-Yehuda, S., Losick, R. & Kolter, R. Fruiting body formation by Bacillus subtilis. *Proc. Natl. Acad. Sci.* **98**, 11621–11626 (2001).
2. Schroeder, J. W. & Simmons, L. A. Complete Genome Sequence of Bacillus subtilis Strain PY79. *Genome Announc.* **1**, (2013).
3. López, D., Fischbach, M. A., Chu, F., Losick, R. & Kolter, R. Structurally diverse natural products that cause potassium leakage trigger multicellularity in Bacillus subtilis. *Proc. Natl. Acad. Sci. U. S. A.* **106**, 280–5 (2009).
4. Straight, P. D., Fischbach, M. A., Walsh, C. T., Rudner, D. Z. & Kolter, R. A singular enzymatic megacomplex from Bacillus subtilis. *Proc. Natl. Acad. Sci. U. S. A.* **104**, 305–10 (2007).
5. Ogran, A. *et al.* The plant host induces antibiotic production to select the most-beneficial colonizers. *Appl. Environ. Microbiol.* **85**, (2019).
6. Koo, B. M. *et al.* Construction and Analysis of Two Genome-Scale Deletion Libraries for Bacillus subtilis. *Cell Syst.* **4**, 291-305.e7 (2017).
7. Steinberg, N. *et al. The extracellular matrix protein TasA is a developmental cue that maintains a motile subpopulation within Bacillus subtilis biofilms*. *Sci. Signal* **13**, (2020).
8. Guérout-Fleury, A. M., Shazand, K., Frandsen, N. & Stragier, P. Antibiotic-resistance cassettes for Bacillus subtilis. *Gene* **167**, 335–6 (1995).
9. Chai, Y., Chu, F., Kolter, R. & Losick, R. Bistability and biofilm formation in Bacillus subtilis. *Mol. Microbiol.* **67**, 254–263 (2007).
10. Peleg, Y. & Unger, T. Application of the Restriction-Free (RF) Cloning for Multicomponents Assembly. in *Methods in molecular biology (Clifton, N.J.)* **1116**, 73–87 (2014).
11. Zhang, H. *et al.* Mega primer-mediated molecular cloning strategy for chimaeragenesis and long DNA fragment insertion. *Biosci. Rep.* **37**, (2017).
12. Wach, A. PCR-synthesis of marker cassettes with long flanking homology regions for gene disruptions in S. cerevisiae. *Yeast* **12**, 259–265 (1996).

13. Wilson, G. A. & Bott, K. F. Nutritional factors influencing the development of competence in the Bacillus subtilis transformation system. *J. Bacteriol.* **95**, 1439–49 (1968).

14. You, C., Zhang, X.-Z. & Zhang, Y.-H. P. Simple cloning via direct transformation of PCR product (DNA Multimer) to Escherichia coli and Bacillus subtilis. *Appl. Environ. Microbiol.* **78**, 1593–5 (2012).

15. Radeck, J. *et al.* The Bacillus BioBrick Box: generation and evaluation of essential genetic building blocks for standardized work with Bacillus subtilis. *J. Biol. Eng.* **7**, 29 (2013).

16. Ganin, H. *et al.* Indole derivatives maintain the status quo between beneficial biofilms and their plant hosts. *Mol. Plant-Microbe Interact.* **32**, 1013–1025 (2019).

17. Shah, I. M., Laaberki, M. H., Popham, D. L. & Dworkin, J. A Eukaryotic-like Ser/Thr Kinase Signals Bacteria to Exit Dormancy in Response to Peptidoglycan Fragments. *Cell* **135**, 486–496 (2008).

18. Chateau, A., Schneewind, O. & Missiakas, D. Extraction and purification of wall-bound polymers of gram-positive bacteria. in *Methods in Molecular Biology* **1954**, 47–57 (Humana Press Inc., 2019).
