# Supplementary material for: Resolving the conflict between antibiotic production and rapid growth by recognition of peptidoglycan of susceptible competitors
Source: Nat Commun. 2022 Jan 20;13:431. doi: 10.1038/s41467-021-27904-2 (PMC8776889; doi:10.1038/s41467-021-27904-2)
Supplement: Supplementary file 2 — Description of Additional Supplementary Files [file 41467_2021_27904_MOESM2_ESM.pdf]

**Description of Supplementary Data Files**

**File Name: Supplementary Data 1**

Description:  File showing p- values from main figure 3, 4a, 4b, 4d, 5a, 5b, 5d, 6a, 8a, 8b, 8d and Supplementary figure 23.
