# Supplementary material for: Resolving the conflict between antibiotic production and rapid growth by recognition of peptidoglycan of susceptible competitors
Source: Nat Commun. 2022 Jan 20;13:431. doi: 10.1038/s41467-021-27904-2 (PMC8776889; doi:10.1038/s41467-021-27904-2)
Supplement: Supplementary file 4 — Reporting Summary [file 41467_2021_27904_MOESM4_ESM.pdf]

nature portfolio

Corresponding author(s): IKG Last

updated by author(s): 06122021

# Reporting Summary

Nature Portfolio wishes to improve the reproducibility of the work that we publish. This form provides structure for consistency and transparency in reporting. For further information on Nature Portfolio policies, see our Editorial Policies and the Editorial Policy Checklist.

## Statistics

For all statistical analyses, confirm that the following items are present in the figure legend, table legend, main text, or Methods section.

| n/a | Confirmed | |
|---|---|---|
| ☐ | ☒ | The exact sample size ($n$) for each experimental group/condition, given as a discrete number and unit of measurement |
| ☐ | ☒ | A statement on whether measurements were taken from distinct samples or whether the same sample was measured repeatedly |
| ☐ | ☒ | The statistical test(s) used AND whether they are one- or two-sided<br>*Only common tests should be described solely by name; describe more complex techniques in the Methods section.* |
| ☐ | ☒ | A description of all covariates tested |
| ☐ | ☒ | A description of any assumptions or corrections, such as tests of normality and adjustment for multiple comparisons |
| ☐ | ☒ | A full description of the statistical parameters including central tendency (e.g. means) or other basic estimates (e.g. regression coefficient) AND variation (e.g. standard deviation) or associated estimates of uncertainty (e.g. confidence intervals) |
| ☐ | ☒ | For null hypothesis testing, the test statistic (e.g. $F$, $t$, $r$) with confidence intervals, effect sizes, degrees of freedom and $P$ value noted<br>*Give P values as exact values whenever suitable.* |
| X | ☐ | For Bayesian analysis, information on the choice of priors and Markov chain Monte Carlo settings |
| X | ☐ | For hierarchical and complex designs, identification of the appropriate level for tests and full reporting of outcomes |
| X | ☐ | Estimates of effect sizes (e.g. Cohen's $d$, Pearson's $r$), indicating how they were calculated |

*Our web collection on statistics for biologists contains articles on many of the points above.*

## Software and code

Policy information about availability of computer code

| | |
|---|---|
| Data collection | No software was used for data collection |
| Data analysis | The following software were used for this manuscript: Python, GraphPad prism 9 and FACS DIVA |

For manuscripts utilizing custom algorithms or software that are central to the research but not yet described in published literature, software must be made available to editors and reviewers. We strongly encourage code deposition in a community repository (e.g. GitHub). See the Nature Portfolio guidelines for submitting code & software for further information.

## Data

Policy information about availability of data

All manuscripts must include a data availability statement. This statement should provide the following information, where applicable:

- Accession codes, unique identifiers, or web links for publicly available datasets
- A description of any restrictions on data availability
- For clinical datasets or third party data, please ensure that the statement adheres to our policy

*Provide your data availability statement here.*

# Field-specific reporting

Please select the one below that is the best fit for your research. If you are not sure, read the appropriate sections before making your selection.

☒ Life sciences ☐ Behavioural & social sciences ☐ Ecological, evolutionary & environmental sciences

For a reference copy of the document with all sections, see nature.com/documents/nr-reporting-summary-flat.pdf

# Life sciences study design

All studies must disclose on these points even when the disclosure is negative.

| | |
|---|---|
| Sample size | Sample size was determined as at least three independent repeats performed with technical repeats |
| Data exclusions | No data was excluded |
| Replication | Sample size was determined as at least three independent repeats performed with technical repeats. Statistical analysis was provided to all experiments included in the manuscript. All attempts were successful and single measurements are indicated and included |
| Randomization | NA, the experiments were performed as advised by our statistician |
| Blinding | NA, No experiments in human patients were performed |

# Reporting for specific materials, systems and methods

We require information from authors about some types of materials, experimental systems and methods used in many studies. Here, indicate whether each material, system or method listed is relevant to your study. If you are not sure if a list item applies to your research, read the appropriate section before selecting a response.

## Materials & experimental systems

| n/a | Involved in the study | |
|---|---|---|
| ☒ | ☐ | Antibodies |
| ☒ | ☐ | Eukaryotic cell lines |
| ☒ | ☐ | Palaeontology and archaeology |
| ☒ | ☐ | Animals and other organisms |
| ☒ | ☐ | Human research participants |
| ☒ | ☐ | Clinical data |
| ☒ | ☐ | Dual use research of concern |

## Methods

| n/a | Involved in the study | |
|---|---|---|
| ☒ | ☐ | ChIP-seq |
| ☐ | ☒ | Flow cytometry |
| ☒ | ☐ | MRI-based neuroimaging |

# Flow Cytometry

## Plots

Confirm that:

☒ The axis labels state the marker and fluorochrome used (e.g. CD4-FITC).

☒ The axis scales are clearly visible. Include numbers along axes only for bottom left plot of group (a 'group' is an analysis of identical markers).

☒ All plots are contour plots with outliers or pseudocolor plots.

☒ A numerical value for number of cells or percentage (with statistics) is provided.

## Methodology

| | |
|---|---|
| Sample preparation | Starter cultures of the WT B. subtilis (harbouring no fluorescent reporter), indicated fluorescent reporters were spotted on B4 plates. For competition, assay fluorescent reporters were spotted 0.4 cm apart from their competitors. The plates were then incubated at 30°C and biofilms colonies were harvested at 24 hours post-inoculation by scrapping the entire colony of the reporters and suspending the colony in PBS. This was followed by sonication using a BRANSON digital sonicator, at an amplitude of 10% and a pulse of 5 sec to separate cells without compromising their viability. Harvesting cells at 24 hours allowed us to harvest cells prior to direct contact with their competitors, thus avoiding any potential mixing of reporters and their competitors. |
| Instrument | LSR-II cytometer (Becton Dickinson, San Jose, CA, USA). |

| | |
|---|---|
| Software | flow cytometry analyses was performed using FACS Diva (BD biosciences). |
| Cell population abundance | . A total of 100,000 cells were counted for each sample |
| Gating strategy | To distinguish background fluorescence from the reporters' specific fluorescence, the WT B. subtilis was used as a negative control, and its background fluorescence was gated to separate true fluorescent population (population outside the background gate) from the reporters. |

☒ Tick this box to confirm that a figure exemplifying the gating strategy is provided in the Supplementary Information.
